# Docking-guided rational engineering of a macrolide glycosyltransferase glycodiversifies epothilone B

**DOI:** 10.1038/s42003-022-03047-y

**Published:** 2022-01-27

**Authors:** Peng Zhang, Lijuan Zhang, Xukai Jiang, Xiao-tong Diao, Shuang Li, Dan-dan Li, Zheng Zhang, Junqiang Fang, Ya-jie Tang, Da-lei Wu, Changsheng Wu, Yue-zhong Li

**Affiliations:** 1 State Key Laboratory of Microbial Technology, Institute of Microbial Technology, Shandong University, Qingdao, 266237 P.R. China; 2 National Glycoengineering Research Center, Shandong Provincial Key Laboratory of Glycochemistry and Glycobiology, Shandong University, Qingdao, Shandong 266237 P.R. China

**Keywords:** Biocatalysis, Glycobiology, Transferases

## Abstract

Glycosyltransferases typically display acceptor substrate flexibility but more stringent donor specificity. BsGT-1 is a highly effective glycosyltransferase to glycosylate macrolides, including epothilones, promising antitumor compounds. Here, we show that BsGT-1 has three major regions significantly influencing the glycodiversification of epothilone B based on structural molecular docking, “hot spots” alanine scanning, and site saturation mutagenesis. Mutations in the PSPG-like motif region and the C2 loop region are more likely to expand donor preference; mutations of the flexible N3 loop region located at the mouth of the substrate-binding cavity produce novel epothilone oligosaccharides. These “hot spots” also functioned in homologues of BsGT-1. The glycosides showed significantly enhanced water solubility and decreased cytotoxicity, although the glycosyl appendages of epothilone B also reduced drug permeability and attenuated antitumor efficacy. This study laid a foundation for the rational engineering of other GTs to synthesize valuable small molecules.

## Abstract (web summary)

Zhang et al. identify three hot regions of macrolide glycosyltransferase BsGT-1, that can play a role in epothilone B and sugar donor specificity. Further mutagenesis of BsGT-1 showed that specificity could be engineered and when the mutations were introduced into homologous GTs, promiscuity was improved, setting the stage for further rational glycodiversification of macrolides.

## Introduction

Epothilones are a class of 16-membered antitumor macrolides isolated from *Sorangium cellulosum* strains. As epothilones exhibit potent bioactivities, especially for killing multidrug-resistant tumors^1^, they have attracted a plethora of research interest ever since discovery^2,3^. Epothilones A and B^1^ are the two major members of this family. In comparison to epothilone A, epothilone B (EpoB) contains an additional methyl group at the epoxy ring but shows stronger potency in antitumor activity^4,5^. Ixabepilone, a semisynthetic analog of EpoB, was approved by the FDA in 2007 for the treatment of unresponsive aggressive metastatic or locally advanced breast cancer^6,7^. However, the clinical usage of EpoB analogs is still greatly restricted due to severe side effects and poor bioavailability^8,9^. Therefore, modification(s) appears necessary to overcome the drawbacks of EpoB, whereby glycosylation could potentially come into play, as it is a powerful approach to tune the physicochemical properties and/or biological activities of small molecules^10,11^. Compared to chemical synthesis, glycosylation reactions catalyzed by enzymes have many advantages, such as high efficiency, regio- and/or stereoselectivity, and environmental compatibility.

Glycosyltransferases (GTs) are natural biocatalysts that transfer glycosyls from activated sugar donors to diverse acceptors^12^. Topologically, GTs are predominantly classified into GT-A and GT-B folds. The GT-A fold possesses a central β-sheet surrounded by α-helices and two tightly abutting β/α/β domains, with a highly conserved metal-coordinating DxD (ASP-X-ASP) motif. The GT-B fold consists of two separate and flexibly linked Rossmann-like domains, and the catalytic cleft is located between these two domains. The GT-B fold contains a relatively conserved sugar donor binding motif at the C-terminal domain, whereas the N-terminal domain interacting with acceptors is usually variable, exhibiting greater topological plasticity^11,13^. This peculiarity allows many GT-B folds to show flexible substrate-binding patterns that are known as “enzyme promiscuity”^14,15^. While the GT superfamily can be classified into 114 families, the members in the GT1 family that typically adopt the typical GT-B fold are invariably promiscuous enzymes^16,17^. In contrast, the GT1 family has evolved higher specificity for sugar donors by accepting a narrow range of NDP-sugars (nucleoside diphosphate sugars). A highly conserved motif in the C-terminal domain, namely, the “PSPG (or -like) motif”, found in GTs from plants and bacteria^18^, is known as the major region for the binding of sugar donors. Many studies have focused on the PSPG motif to flexibilize sugar donor specificity; however, site-directed mutagenesis of the PSPG motif often leads to complete loss^19^ or severe damage^20,21^ in enzyme activity. More intriguingly, site-directed mutagenesis outside the PSPG motif of the two GTs also broadened the sugar donor scope, indicating that the PSPG motif is probably not the only determinant in sugar donor recognition^22,23^. With the surge in genome sequencing, a vast number of GTs have been unraveled, but only a very small portion have been characterized confirmatively in function^18^. In particular, investigations into the underlying structural basis of GTs that determines sugar donor specificity remain insufficient, which limits rational engineering of GTs for glycorandomization of bioactive molecules.

We previously characterized the promiscuous enzyme BsGT-1, which displayed highly efficient glycosylation activities toward diverse acceptors^24^, including the macrolides epothilone A and rapamycin^25,26^. In this study, we applied BsGT-1 to the glycosylation of EpoB in an attempt to glycodiversify and better this medicinally important small molecule. Directed by molecular docking analysis in tandem with mutagenesis study, we systematically probed functional regions of BsGT-1 and revealed the “hot spots” in three major regions that determine the catalytic efficiency and/or sugar donor specificity. This enabled us to concisely synthesize and characterize seven previously undescribed EpoB glycosides, differentiating in the sugar category and/or size. The glycosyls appended to EpoB significantly improved the water solubility but reduced the antitumor efficacy and drug permeability into cells. The fact that the “hot spots” of BsGT-1 also existed in its orthologues indicated that our work will lay foundations for the future rational engineering of different GTs for the glycodiversification of natural biomolecules with sheer chemical complexity and pronounced bioactivities.

## Results

### BsGT-1 glycosylated epothilone B using different sugar donors

**Fig. 1:**
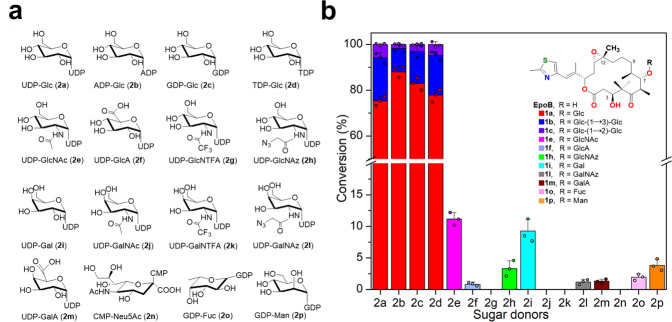
The sugar donor specificities of wild-type BsGT-1. **a** Structures of the 16 tested sugar donors (**2a**−**2p**). **b** Conversion efficiency of wild-type BsGT-1 toward sugar donors **2a**−**2p** after 10 h of incubation. The structures of epothilone B (EpoB) and its glycosides catalyzed by mutants of BsGT-1 are shown, the conversion data (mean ± SD) were obtained from three independent repeat experiments.

We previously characterized that BsGT-1 could efficiently glycosylate epothilone A at the 7-OH position with UDP-glucose (UDP-Glc, **2a**)^25^. Herein, we further tested the glycosylation ability of BsGT-1 toward epothilone B (EpoB). When using **2a** as the sugar donor, HPLC-DAD profiling (249 nm) indicated that EpoB was almost 100% converted by BsGT-1 after 2 h of glycosylation reaction, and the efficiency was congruent with that of epothilone A^25^. Unexpectedly, three glycosylated products **1a**, **1b**, and **1c** were generated for EpoB (Supplementary Fig. 1), in contrast to the single monoglycoside product for epothilone A. Major product **1a** achieved >90% conversion within an hour followed by a gradual decrease, while **1b** and **1c** increased steadily. After purification, compounds **1a**–**1c** were identified as EpoB 7-*O*-β-D glucoside, EpoB 7-*O*-β-D-glucosyl-(1 → 3)-β-D glucoside, and EpoB 7-*O*-β-D-glucosyl-(1 → 2)-β-D glucoside on the basis of HRMS (high-resolution mass spectrometry) and NMR (nuclear magnetic resonance) (Fig. 1 and Supplementary Table 1, Supplementary Figs. 15–17 and Supplementary Figs. 27–41), respectively. The stepwise tandem assembly of two glucoses by a single GT in a non-regiospecific manner (1 → 3 or 1 → 2 connectivity) is remarkable, which was reminiscent of OleD’s triple mutant (A242V/S132F/P67T) that was able to mediate disaccharide formation through iterative glucosylation^27^, and AveBI that can consecutively integrate two molecules of l-oleandrose to avermectin^28^.

We further probed the sugar donor promiscuity of BsGT-1 towards 16 activated forms of monosaccharides (**2a**–**2p**, Fig. 1a) in an attempt to glycodiversify EpoB. After 10 h of incubation, the four NDP-glucoses (**2a**–**2d**) achieved almost 100% turnover of EpoB, and low conversion efficiency (<20%) was observed for other sugar donors, including **2e**, **2f**, **2h**, **2i**, **2l**, **2m**, **2o**, and **2p** (Fig. 1b), whereby the corresponding monoglycosylated products **1e**, **1f**, **1h**, **1i**, **1l**, **1m**, **1o**, and **1p** were confirmed by HRMS/(MS) (Supplementary Figs. 19–26) and/or NMR (Supplementary Table 2 and Supplementary Figs. 48–62). The tolerance of different NDP-sugars by BsGT-1 implied the sugar donor promiscuity of BsGT-1 and its engineering potential for the glycorandomization of EpoB.

### Structure of BsGT-1 and its docking model with substrates

**Fig. 2:**
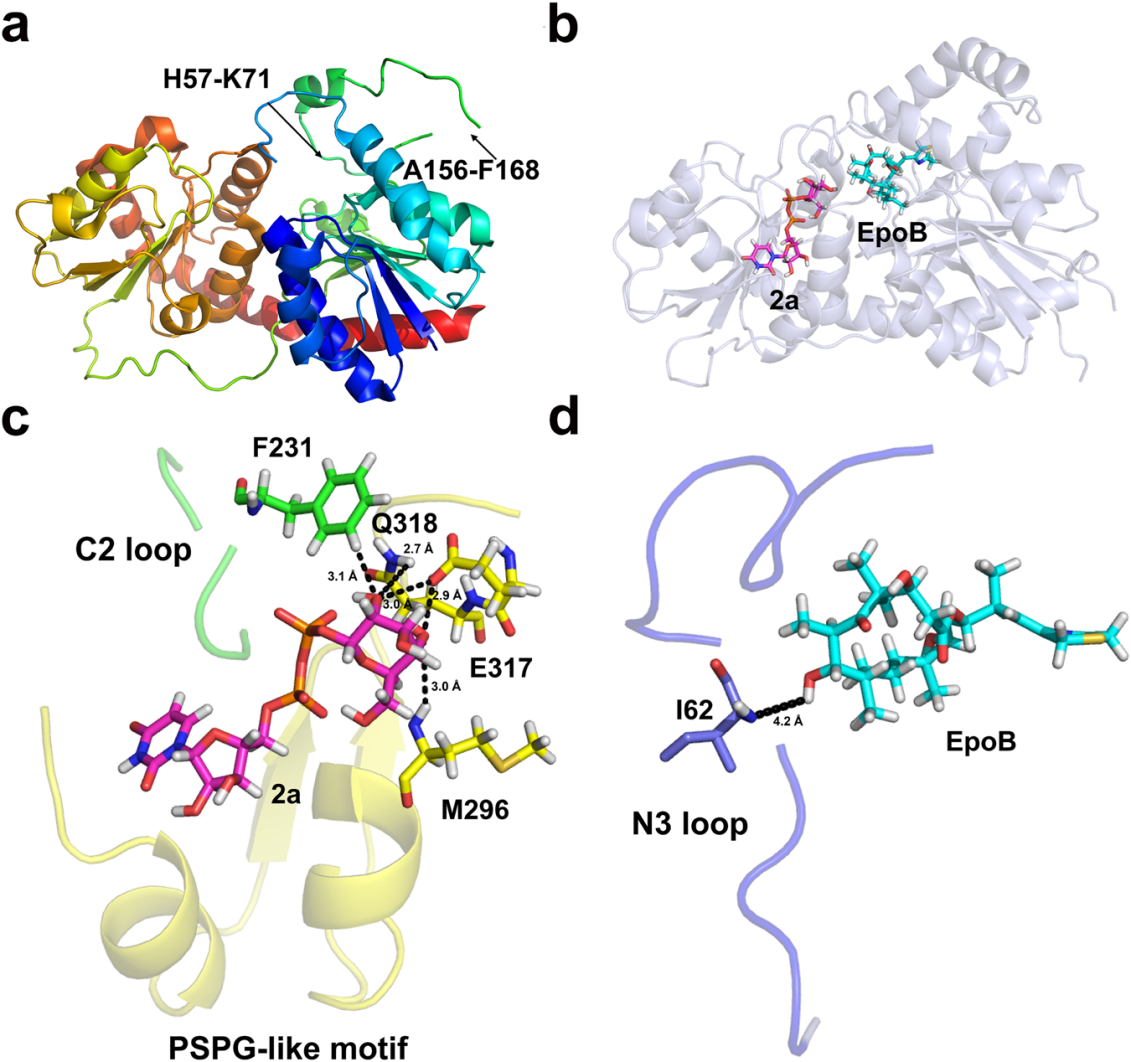
The interactions between the “hot spots” and substrates in the docking model of BsGT-1. **a** The incomplete crystal structure of BsGT-1 with resolution at 2.29 Å (PDB ID: 7BOV). **b** The predicted complete structure of BsGT-1 was docked with the sugar acceptor EpoB and sugar donor UDP-Glc (**2a**). **c** The potential hydrogen bond interactions of M296, E317, and Q318 in the PSPG-like motif (surface, yellow) and F231 in loop C2 toward the glucosyl moiety of **2a**. **d** The positional relationship between loop N3 region and EpoB, whereby the distance between the I62 and EpoB was indicated.

**Fig. 3:**
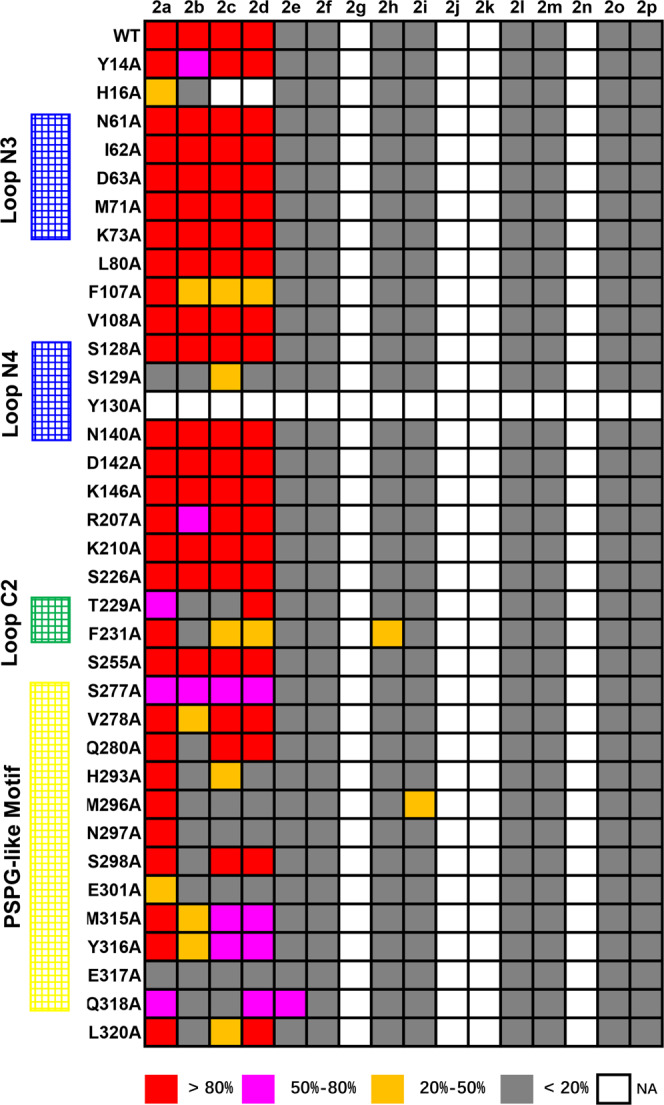
Profiling of the sugar donor specificities of the 35 alanine-scanning mutants. The conversion efficiency of EpoB using 16 different sugar donors after 10 h of incubation was divided into five different ranks (NA, no activity detected). The key functional regions (PSPG-like motif, C2, N3, and N4 loop regions) were labeled.

To gain deeper insight into the catalytic mechanism of BsGT-1, we tried to resolve the structure of wild-type BsGT-1. While we made to obtain a crystal structure with low resolution, a higher quality of crystal structure of BsGT-1 (Bs-YjiC) at 2.29 Å was disclosed by another research group (accession number: 7BOV, Fig. 2a)^29^. BsGT-1 adopts a typical GT-B fold, consisting of two distinct Rossmann-like domains interconnected by a linker region. However, the resolved structure of BsGT-1 (7BOV) is incomplete because two flexible loop regions (residues H57–M71 and A156–F168) with low electron density could not be modeled. To investigate the potential impacts of these two regions on enzyme activity, we deleted residues H57–M71 and A156–F168 respectively from wild-type BsGT-1, and the resulting mutants were both solubly expressed in *E. coli* (Supplementary Fig. 2). As a result, the deletion of A156-F168 had negligible effects on the glycosylation of EpoB, whereas the ΔH57–M71 mutation caused a severe reduction in catalytic efficiency (Supplementary Fig. 3). In view of this, we used the I-TASSER method and crystal structure 7BOV as a template to restore the complete structure of wild-type BsGT-1^30^ and then subjected it to docking analysis using AutoDock Vina and molecular dynamics simulation of GROMACS (Fig. 2b)^31,32^. To exploit the catalytic characteristics of BsGT-1 toward substrates EpoB and **2a**. The free binding energy (ΔG) was used to characterize the maximum energy of reversible binding of ligands with protein (Supplementary Table 3), and the 35 residues that formed hydrogen bonds or van der waal’s force interactions with ligands of EpoB and **2a** were pinpointed (Fig. 2b and Supplementary Table 4). Subsequently, these potential key residues were subjected to alanine scanning mutagenesis to probe their respective functions for EpoB glycosylation towards the 16 different sugar donors **2a**–**2p** displayed in Fig. 1a. The catalytic efficiency of the mutants was classified into five different ranks according to the turnover rate of EpoB (Fig. 3). The mutations at H16 (in Nα1) and S129 (in the N4 loop) that simultaneously interacted with EpoB and **2a** (Supplementary Table 4 and Supplementary Fig. 4) caused a dramatic reduction in catalytic efficiency, while the mutation at Y130 (in the N4 loop) that interacted with EpoB absolutely abrogated glycosylating ability. This was consistent with previous results that the residues in the N4 loop of GTs played important roles in substrate catalysis^17^. Interestingly, many mutations outside the loop N4 region also exhibited evident impacts on either sugar donor recognition or polysaccharification, which compelled us to further investigate the potential “hot spots” in the PSPG-like motif, C2 loop, and N3 loop, as described in the following sections.

### The important role of the PSPG-like motif in the sugar donor promiscuity of BsGT-1

The PSPG motif, known to be closely related to sugar binding and recognition, is conserved in most plant GTs, and a PSPG-like motif is also commonly distributed in bacterial GTs^18,33^. It was reported that mutations of key residues in the PSPG motif usually abolished activity or only caused weak alterations in sugar donor recognition^34^. We performed alanine-scanning mutagenesis in the PSPG-like motif of BsGT-1, and the results showed that glycosylation with **2a** was less affected than the other three NDP-glucoses (**2b**–**2d**), since all the mutants of the PSPG-like motif retained conversion unchanged for **2a**, or at least better than the other three NDP-glucoses (Fig. 3). Notably, the PSPG-like motif usually begins with the residue Trp and ends with the strictly conserved Glu/Asp-Gln cassette that is critical for hydrogen-bonding interaction with the 2-OH of glucosyl moiety^17,35,36^. The uracil base of **2a** contacts the aromatic side chain of W312 in OleI or W290 in OleD, rendering the preference for **2a** rather than other NDP-glucose^17,37^. Distinctively, BsGT-1 begins with serine in the PSPG-like motif (S277) (Supplementary Fig. 4) and can accept all NDP-glucoses irrespective of nucleotides. The S277A mutation in BsGT-1 caused a considerable decline in the activity (Fig. 3). Moreover, most alanine-scanning mutants of the PSPG-like motif normally decreased the activities toward the other three NDP-glucoses rather than UDP-glucose, while mutants E301A and E317A had weak activities toward all four NDP-glucoses.

**Fig. 4:**
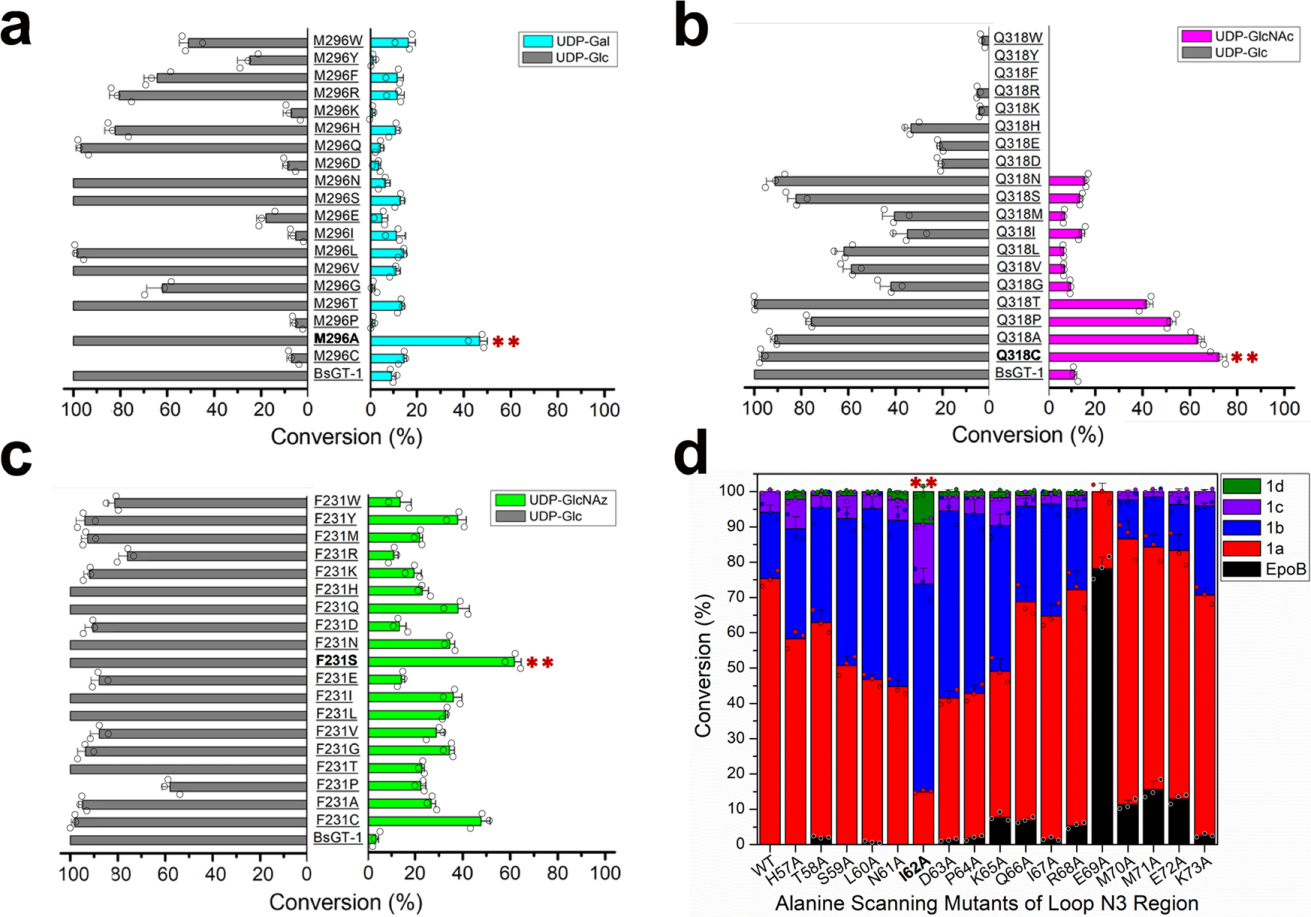
Profiling of the enzymatic activities of saturated mutants of the “hot spots” and functional investigation of the N3 loop. **a** Comparison of the conversion efficiency (mean ± SD) of M296 saturated mutants using UDP-Gal and UDP-Glc as sugar donors, and the M296A mutant with the highest efficiency toward UDP-Gal was highlighted. **b** Comparison of the conversion efficiency of Q318 saturated mutants using UDP-GlcNAc and UDP-Glc as sugar donors, and the Q318C mutant with the highest efficiency toward UDP-GlcNAc was highlighted. **c** Comparison of the conversion efficiency of F231 saturated mutants using UDP-GlcNAz and UDP-Glc as sugar donors, and the F231S mutant with the highest efficiency toward UDP-GlcNAc was highlighted. **d** The change in the proportions of EpoB glucosides **1a**–**1d** caused by alanine scanning mutation in the loop N3 region. The I62A mutant that gave the highest portion of polyglycosides (**1b**–**1d**) was highlighted, the conversion data were obtained from three independent repeat experiments.

Our docking model revealed that E317 had hydrogen bonds with both 2-OH (3.0 Å) and 4-OH (2.9 Å) of glucose. Residue Q318 also formed an interaction (2.7 Å) with the 2-OH of glucose, and M296 potentially formed a hydrogen bond (3.0 Å) with the 4-OH of glucose (Fig. 2c). E317 seemed to be more important than Q318 in capturing the glucosyl moiety because the saturation mutagenesis of E317 almost abolished the conversion activity towards **2a** (Supplementary Fig. 5). Notably, although wild-type BsGT-1 gave a relatively low conversion efficiency toward UDP-Gal (**2i**, 9.3%) and UDP-GlcNAc (**2e**, 11.2%), their conversion efficiencies were significantly improved by M296A and Q318A mutations, respectively (Fig. 3). Further saturation mutagenesis proved that the M296A mutant was most efficient in **2i** catalysis, giving a 10-fold higher catalytic efficiency (*kcat/km* value, Supplementary Table 5) than WT (Fig. 4a). The Q318C mutation achieved >70% conversion of **2e**, in stark contrast to ~10% by wild-type BsGT-1 (Fig. 4b). Taken together, the PSPG-like motif plays an important role in the sugar donor specificity of BsGT-1 and glycodiversification of EpoB.

### The C2 loop is also involved in the sugar donor promiscuity of BsGT-1

In addition to the PSPG-like motif, some C-terminal residues also formed intimate interactions with EpoB and/or UDP-Glc (**2a**). Alanine-scanning mutagenesis showed that the R207A, K210A, S226A, and S255A mutations had little impact on the catalytic efficiency and sugar donor specificity of BsGT-1 (Fig. 3). However, T229 and F231, located within the loop C2 region (Supplementary Fig. 6), were highly related to sugar donor specificity. The T229A mutation had a detrimental effect on the binding of NDP-glucoses, except TDP-glucose. Although the F231A mutation affected the interactions with NDP-glucoses except **2a**, the catalytic activity toward UDP-GlcNAz (**2h**) of this mutant was surprisingly enhanced (Fig. 3). Notably, the aromatic ring of residue F231 had a close interaction with 2-OH (3.1 Å) of glucose in **2a** (Fig. 2c), and the replacement with less bulky alanine might allow the entry of **2h** endowed with a large substituent at 2-OH of glucose. Saturation mutagenesis showed that the conversion of **2h** was further improved by the F231S mutation, reaching >45% (Fig. 4c). The *k*_*cat*_*/Km* value of F231S toward **2h** was 22.5-fold higher than that of WT (Supplementary Table 5). Nevertheless, most of the saturated mutants of F231 retained potent activity toward **2a**, except that the activity of F231P was reduced by half (Fig. 4c). Thus, the disordered C2 loop outside of the PSPG-like motif is also involved in maintaining sugar donor specificity.

### The N3 loop of BsGT-1 is related to the polysaccharification of EpoB

It is well known that the N-domain is responsible for the binding of sugar acceptors, and the C-domain holds the sugar donor in GT-B fold glycosyltransferase^11,18^. We performed alanine-scanning mutagenesis at some residues in the N-domain (from Y4 to I199) that did not exhibit any influence on sugar donor promiscuity (Fig. 3), but the H16A mutation indeed hampered EpoB binding. Interestingly, although the mutants of N61A, I62A, and D63A gave 100% conversion of EpoB as wild type did, the proportion of the generated EpoB glucosides (**1a**, **1b**, **1c**) was substantially changed (Fig. 4d), and especially a new compound **1d** was produced by these three mutants but not by wild type. Based on the combination of HRMS and intensive 2D NMR interpretation (Supplementary Fig. 18 and Supplementary Figs. 42–47), **1d** was identified as an unprecedented linear trisaccharide, epothilone B 7-*O*-β-D-glucosyl-(1 → 2)-β-D-glucosyl-(1 → 4)-β-D glucoside (**1d**, Supplementary Fig. 7). The generation of three types of glycosidic bonds (1 → 3 in **1b**, 1 → 2 in **1c** and **1d**, and 1 → 4 in **1d**) suggested flexibility in terms of the regioselectivity of BsGT-1.

In BsGT-1, the N3 loop region is located at the entrance of the cleft between the N-domain and C-domain, wherein the I62 residue was 4.2 Å away from C-7 of EpoB in the docking model (Fig. 2d). Because residues N61, I62, and D63 are located in the same N3 loop, we proposed that this region determined polysaccharification. To experimentally confirm this, we carried out systematic alanine scanning of the whole N3 loop region (from Y57 to K73), and each mutant was assessed as a catalyst for glucosylation with **2a** as the donor. As a result, polysaccharification peaked at the I62A mutation and then gradually decreased to both sides (Fig. 4d). The I62A mutant dominantly produced diglucosylated derivatives (**1b** and **1c**, accounting for 75%) and a decent amount of **1d** (accounting for 10%). Further saturation mutagenesis at site I62 showed that the substitutions of bulky amino acids, such as I62H, I62K, I62F, and I62W, severely affected the enzymatic activity, while the residues with small side chains, such as I62G and I62A, retained both the conversion efficiency and the iterative glucosylation capability; I62A was still the most advantageous mutation for polysaccharification (Supplementary Fig. 8). Notably, the E69A mutation markedly decreased the conversion of epothilone B.

The catalytic efficiency for EpoB→**1a** conversion by I62A and wild type was comparable, but the *k*_*cat*_*/K*_*m*_ values of **1a** → **1b** and **1a** → **1c** by I62A were 28.3- and 5.3-fold higher than the wild type, respectively (Supplementary Table 5). The comparatively higher conversion rate of **1a** → **1b** than **1a** → **1c** by the I62A mutant could be explanatory to the limited production of **1d**, since it was generated on the basis of **1c**. Taken together, the dramatic fluctuation in the iterative glycosylation represented by the I62A mutation indicated that the N3 loop is very likely associated with the formation of a catalytic cavity for acceptor EpoB.

### The “hot spots” of BsGT-1 also exist in other homologous GTs

**Fig. 5:**
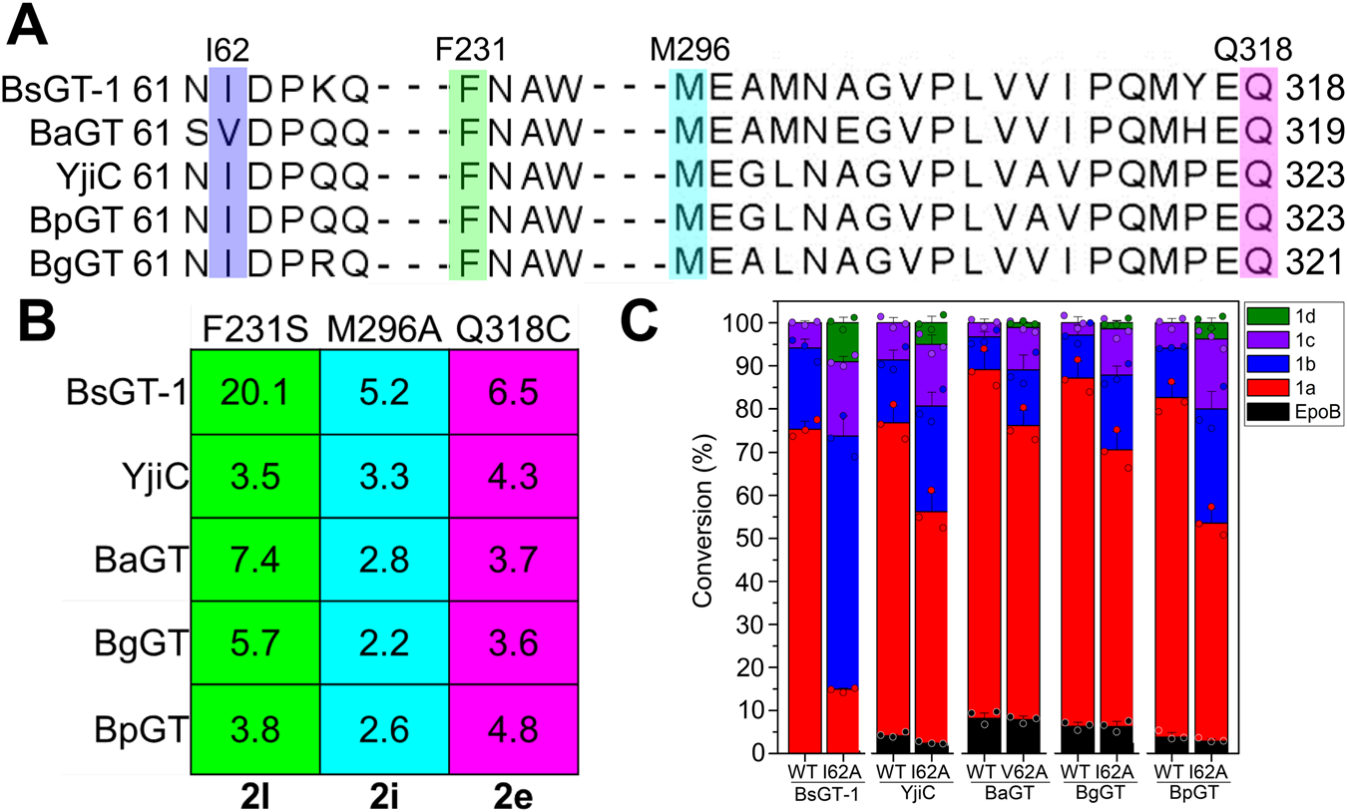
The “hot spots” characterized in BsGT-1 also exist in its homologs. **a** The alignment of BsGT-1 with its four homologs YjiC, BaGT, BgGT, and BpGT, wherein the “hot spots” of I62, F231, M296, and Q318 characterized in BsGT-1 were highlighted. **b** The elevated conversion efficiency towards the sugar donors UDP-GlcNAz (**2h**), UDP-Gal (**2i**), and GlcNAc (**2e**) achieved by the mutations of F231S, M296A, and Q318C, respectively. The displayed values (fold change) are the conversion efficiency of mutants relative to the respective wild type. **c** Comparison of the conversion rate (mean ± SD) of glycosides **1a**–**1d** by the mutants (I62A or V62A) and wild type (WT). The 62^nd^ site in the five GTs was uniformly related to polysaccharification because the proportion of monoglycoside **1a** was reduced and the polyglycosides (**1b**–**1d**) in the mutant were accordingly increased, the conversion data were obtained from three independent repeat experiments, the displayed fold changes were calculated based on the averages of the conversion data.

Sequence alignment of the regions of the PSPG-like motif, N3 loop, and C2 loop in ten related GTs (Supplementary Table 6) revealed that YjiC, BaGT, BgGT, and BpGT were highly conserved at the “hot spots” characterized in BsGT-1 (Supplementary Fig. 6 and Supplementary Fig. 9). These four macrolide GTs were further subjected to TM alignment (https://zhanglab.ccmb.med.umich.edu/TM-align/)^38^ to compare their 3D architectures. The topologies of these enzymes, especially for the PSPG-like motif, C2 loop and N3 loop regions, showed a high degree of similarity to BsGT-1 (Supplementary Fig. 10), probably rendering them similar capabilities for glycosylation of EpoB.^25^ We subsequently performed site-directed mutagenesis of BaGT, YjiC, BpGT, and BgGT at the sites corresponding to the “hot spots” of BsGT-1 to probe their putative roles in EpoB glycosylation. As a result, the conclusions drawn for the four “hot spots” (I62, F231, M296, and Q318) of BsGT-1 were actually applicable to the four enzymes YjiC, BaGT, BgGT, and BpGT (Fig. 5a). Specifically, mutagenesis at the two “hot spots” in the PSPG-like motif of these four enzymes corresponding to M296 and Q318 in BsGT-1 significantly increased the catalytic activities toward UDP-Gal (**2i**) and UDP-GlcNAc (**2e**) by ~2.5- and 5-fold, respectively. Likewise, the site mutagenesis of the four GTs corresponding to F231S in BsGT-1 was favorable for the enhanced acceptance of UDP-GlcNAz (**2h**) as a sugar donor (Fig. 5b). The mutations at position 62 of the four GTs gave rise to an elevated ratio in the polysaccharification compared to the wild type, as represented by the new generation of **1d** (Fig. 5c). Collectively, similar improvements in the promiscuity of sugar donors after mutagenesis at the “hot spots” of the four macrolide GTs implied that the structural knowledge revealed for BsGT-1 in this study has somewhat generality, and would provide guidance for the rational engineering of other GTs to glycodiversify a broad range of pharmaceutically important small molecules.

### Biological properties of epothilone B glycosides

We first tested the water solubility of epothilone B (EpoB) glycosides. Not unexpectedly, the more glycosyl groups attached to EpoB, the better solubility was observed, whereby *mono*-, *di*-, and *tri*-glycosylation evidently improved the water solubility of EpoB to 40-, 400-, 1000-fold, respectively (Table 1). Next, the isolated glycosides were subjected to antitumor and cytotoxicity assays using the liver carcinoma cell line HepG2 and hepatocyte cell line HL-7702. Consistent with previous studies^1,2,39^, EpoB exhibited potent antitumor activity at the nanomolar level toward HepG2 cells (IC_50_ < 0.002 μM) but also caused severe cytotoxicity (IC_50_ < 0.003 μM, Table 1). Both the inhibitory effects and cytotoxicities of all the glycosides decreased >1000-fold, despite different extensions depending on the properties of sugar moieties. In general, the size of the sugar moiety negatively correlated with the biological activity, so the IC_50_ values of EpoB with substituents of *mono*-Glc (**1a**), GlcNAc (**1e**), *di*-Glc (**1c**), and *tri*-Glc (**1d**) showed an ascending trend. Notably, the galactose derivative (**1i)** showed the best antitumor activity (IC_50_, 1.09 μM) among the EpoB glycosides. To understand the reason why 7-*O*-glycosylation had a detrimental effect on the bioactivity of EpoB, we conducted molecular docking analysis of the representative monoglycoside **1a** with tubulin (target protein), whereby the cocrystal structure of β-tubulin subunit-EpoB deposited in the PDB database (7DAE) was used as a template model (Supplementary Fig. 11). In comparison, EpoB intimately interacted with the β-tubulin subunit through hydrogen bonding (T276, Q281, and D226) and hydrophobic interactions (L230, F272, and L275), which were disrupted in the docking model for **1a**. However, although the hydrogen bonding between the key residue D226 and the 7-OH group of **1a** was blocked by the appended glycosyl, a new hydrogen bond was formed with the 6-OH group of sugar, which could be explanatory for the appreciable antitumor activity of **1a** (IC_50_, 9.84 μM).

**Table 1:** Water solubility (25°C, mg/mL), antitumor, and cytotoxicity assays of epothilone and its glycosides.

Compounds	Solubility (mg/mL)	IC_50_	
		HepG2 (μM)	HL-7702 (μM)
EpoB	0.19 ± 0.03	0.00139 ± 0.00030	0.00293 ± 0.00053
1a	1.55 ± 0.09	9.84 ± 0.57	27.63 ± 1.76
1b	86.38 ± 1.45	>100	–
1c	83.03 ± 1.54	34.18 ± 4.16	46.61 ± 0.48
1d	184.83 ± 3.50	38.20 ± 2.41	42.75 ± 1.15
1h	4.34 ± 0.54	30.23 ± 2.91	40.41 ± 1.73
1i	3.37 ± 0.55	1.09 ± 0.26	9.13 ± 1.02
1e	8.22 ± 0.93	19.76 ± 0.88	31.07 ± 3.77

**Fig. 6:**
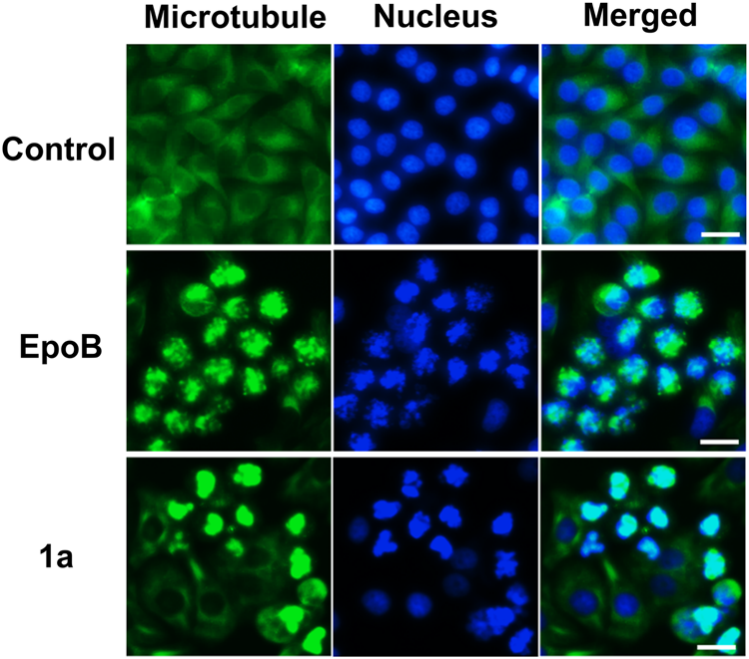
The impacts of glycosylation on the antitumor activities and cellular intake of epothilone B (EpoB). Confocal imaging of HepG2 cells treated with EpoB and **1a** that exhibited effects on tubulin polymerization. Microtubules and DNA were stained green and blue, respectively (scale bar: 20 μm).

As compound penetration into cells plays an important role in their antitumor effects^40^, we further carried out immunofluorescence experiments to compare the cellular intake of EpoB and glycosides **1a** and **1i**. As shown in Figs. 1a, 6 could penetrate into the tumor cells and interact with tubulin, although the amount was less than that of EpoB. Further quantitative analysis showed that the cellular intake rate of glycosides **1a** and **1i** was 12- and 8-fold lower than that of EpoB, which also contributed to the reduced activities of EpoB glycosides (Supplementary Fig. 12).

## Discussion

Glycosylation is an effective way to tune the physicochemical properties and/or bioactivities of various natural products^41^, and glycosyltransferases (GTs) are powerful enzymatic toolkits for this purpose^42^. Wild-type GTs often have limitations in terms of low catalytic efficiency and/or unwanted substrate specificity/selectivity; thus, rational engineering is always needed^43^. Protein structure-guided mutagenesis has been proven to be a rewarding approach to enhance the catalytic activity and/or alter the product specificity of GTs. For instance, the solution of the crystal structure of triterpene glycosyltransferase UGT74AC1 from *Siraitia grosvenorii* facilitated directed evolution to improve enzymatic activity and substrate promiscuity, leading to one variant that exhibited up to 4.17 × 10^4^-fold increase in catalytic efficiency toward the terpenoid mogrol and 1.53 × 10^4^-fold increase for sugar donor UDP-glucose, respectively^44^; γ-Cyclodextrin (γ-CD) is produced from starch by using *Bacillus clarkii* γ‐cyclodextrin glycosyltransferase (CGTase), but this enzymatic process also gives rise to the interfering side products α-CD and β-CD^45^. The site-directed mutagenesis of γ-CGTase significantly improved the product preference toward γ-CD, whereby the mutant Y186W displayed a high selectivity rate of 94.6% versus 77.1% given by the wild type^46^. In recent years, the Ye lab has been productive in solving the 3D structures of plant *O*- and *C*-glycosyltransferases (i.e., TcCGT1^21^, GgCGT^47^, TcOGT4^48^, and Sb3GT1^49^) and accordingly characterized the structural basis that determines the catalytic efficiency and/or substrate specificity, which has greatly promoted the glycodiversification of important flavonoid-class natural products. In this study, we likewise employed structure-guided engineering of the versatile glycosyltransferase BsGT-1^26,29^ to improve its catalytic efficiency towards different sugar donors and guaranteed sufficient yields for the chromatographic purification of seven previously undescribed EpoB glycosides. Hence, the docking-guided engineering of GTs is an effective and economical strategy for the glycosylation of important molecules.

In general, wild-type GTs have a relatively wide spectrum of acceptors but a narrow window of sugar donor selectivity^18^. As diverse sugar moieties can positively fine-tune the physicochemical properties and/or alter the bioactivities of drug acceptors^42,46^, it appears important to understand the structural basis shaping sugar donor preference. The PSPG motif has been regarded as the key region for sugar donor recognition. For instance, the C-glycosyltransferase TcCGT1 from *Trollius chinensis* showed regiospecificity in the *C*-glycosylation of flavones and catalyzed the *O*-glycosylation of diverse phenolics with UDP-Glc. The mutation (E396A) in the PSPG motif of TcCGT1 abolished the catalytic activity^21^. Other cases also reported that mutations in this region lead to a complete loss of enzymatic activity^17,50^, but the single mutation of P361W in the PSPG motif of TcOGT4 switched the sugar donor preference from UDP-Gal to UDP-Glc^48^. Our studies revealed more key residues in this motif that formed hydrogen bonds with the specific hydroxyl groups of sugar moieties, i.e., M296 and Q318 interacted with O4 and O2 of the sugar, respectively. In addition to the PSPG-like motif, the “hot spots” in other motifs could also affect sugar donor selectivity. The triple mutant P67T/I112K/A242V of GT OleD^51^ and the quadruple mutations in S60/V100/T104/I152 of GT MiCGTb^22^ were capable of efficiently catalyzing a set of different sugar donors. Notably, this study also revealed a single “hot spot” (residue F231) in the C2 loop region of BsGT-1, and the untapped C2 loop region is jointly involved in the recognition of sugar donors with PSPG-like motifs. This knowledge will undoubtedly provide more insight into the engineering of GTs for the alteration of sugar donor specificity in the future.

In contrast to the C2 loop, which impacts sugar donor specificity, the N3 loop of BsGT-1 is involved in the recognition of acceptors. Structure-based systematic mutations in the N3 loop indicated that it contributed to the formation of a binding cavity for EpoB (Fig. 4d). This was well reflected by saturated mutagenesis at the I62 site, whereby replacement with the amino acid glycine or alanine with small sider chains was favorable for polysaccharification of EpoB (Supplementary Fig. 8). The polysaccharification capacity of the mutants (I62G and I62A) was very likely attributed to an increase in the volume of the binding pocket to accommodate a larger substrate and to release larger products. Polysaccharification could evidently improve water solubility and is sometimes crucial for the bioactivities of natural products^52^. As loops often undergo a disordered to ordered transition^53^, we further hypothesized that the N3 loop is related to the incredible “substrate promiscuity” of BsGT-1 that accepts various chemical scaffolds, such as macrolides epothilone A^25^ and rapamycin^26^, flavonoids, and coumarins^24^. Presumably, the flexible N3 loop adopts different conformations to adjust a spacious substrate-binding tunnel to accommodate different acceptors. This was reminiscent of loop dynamics of the glycosyltransferase GpgS that underwent multiple conformation changes along the catalytic cycle, wherein the open-to-closed motion of the L loop in GpgS was essential for assembling the reaction center^53^. The conformational plasticity of the N3 loop in BsGT-1 is probably commonplace in GT-B fold GTs. In addition to the evidence presented in Fig. 5c, residue P67 in the N3 loop of OleD has also been proven to be the most advantageous site for expanding its substrate spectrum^54^.

The severe side effects and poor bioavailability of EpoB^8,9^ necessitate chemical modifications, including glycosylation. Regretfully, the cytotoxicity and antitumor activity of the obtained glycosides in this study were dramatically decreased (Table 1). This is because the 7-OH of EpoB is a key functionality that forms hydrogen bonds with target microtubulin, and the bulky substituents at this position caused huge steric hindrances for ligand entry into the binding cavity of the protein (Supplementary Fig. 11). Hence, enzymatic glycosylation at other sites (such as 3-OH) instead of 7-OH should be tried in the future. As the O-glycosyltransferase BsGT-1 could recognize only the two innate hydroxyl groups at C-3 and C-7 of EpoB, characterization of potential C-glycosyltransferases^21,47^ that modify macrolide-class molecules could randomly glycosylate the backbone of EpoB. Alternatively, it is also tempting to develop cascade enzymatic reactions to regioglycodiversify EpoB: first, P450 monooxygenases^55^ are used to introduce extra hydroxyls onto EpoB, and then O-glycosyltransferases are used to install glycosyls. Meanwhile, we noticed that the activity of galactose-modified EpoB (**1i**) was almost 10-fold higher than that of glucose-modified EpoB (**1a**, Table 1), implying that further intensive enzymatic glycosylation studies with more variable sugar donors are still promising to deliver efficacious EpoB derivatives. This hypothesis seems reasonable because most sugar moieties in bioactive macrolides (i.e., erythromycin, avermectin) are uncommon deoxysugars (i.g. desosamine, oleandrose), and they are indeed crucial for interaction with target proteins^56,57^. The availability of unusual sugar donors could be problematic, which could be solved by a synthetic biology approach^58^. On the other hand, we revealed that the decline in the antitumor efficacy of current EpoB glycosides was also related to the lower cellular intake rate. Therefore, the combination of EpoB glycosides with other therapeutics to enhance the penetration of cell membranes will probably elevate the efficacy of EpoB glycosides.

Collectively, through structure-directed engineering (alanine scanning and/or saturated mutagenesis), we systematically explored the structural basis that determines the catalytic efficiency of BsGT-1 and identified the key “hot spots” that are critical for the selectivity of sugar donors and acceptors. This study provides much deeper insights into the catalytic mechanism of promiscuous GT-B fold glycosyltransferases, as represented by BsGT-1, and lays a foundation to make unnatural natural biocatalysts for the concise synthesis of various bioactive glycosides. Furthermore, in view of the obvious shortfalls in the clinical usage of EpoB, the EpoB glycosides obtained in this study differing in the categories and/or iteration pattern of glycosyl(s) have greatly enriched the glycochemistry of this druggable molecule. Although the antitumor activity of EpoB was generally attenuated, the fact that different types of sugar appendages exerted disparate influences on the activity underscored further intensive enzymatic glycosylation with more variable sugar donors to give glycosylated derivatives of EpoB with both appreciable potency and good water solubility.

## Methods

### Reagents, cells, and main media preparation

NDP-glucose (UDPG, ADPG, TDPG, and GDPG), UDP-N-acetylglucosamine (UDP-GlcNAc), UDP-galactose (UDP-Gal), and UDP-glucuronic acid (UDP-GlcA) were purchased from Sigma-Aldrich (St. Louis, MO, USA). UDP-GlcNAz, UDP-GalNAz, UDP-GlcNTFA, UDP-GalNTFA, UDP-GalNAc, UDP-GalA, CMP-Neu5Ac, GDP-Fuc and GDP-Man were kindly provided by Professor Junqiang Fang^59–63^. Epothilone B was purchased from Zhejiang Hisun Pharmaceutical Co., Ltd. The human liver carcinoma cell line HepG2 and human hepatocyte cell line HL-7702 were purchased from Gaining (Gaining Bio. Co., Ltd, Shanghai, China). The cells were cultivated in T-25 flasks in a Thermo Scientific CO_2_ incubator in a humidified environment at 37°C and 5% CO_2_. HepG2 cells were preserved in DMEM (Gibco, Thermo Fisher Scientific, Waltham, MA, USA), while HL-7702 cells were preserved in RPMI-1640 (Gibco, Thermo Fisher Scientific, Waltham, MA, USA). All cultures contained 10% fetal calf serum and 1% penicillin C/streptomycin. All chemicals and reagents are of analytical grade.

### Protein expression, purification, and site-directed or saturated mutagenesis

The recombinant plasmids of BsGT-1, YjiC, BaGT, BgGT, and BpGT were transformed into *E. coli* BL21 (DE3) for protein expression and purification by using previously described protocols^25^. A Fast Mutagenesis Kit V2 (Vazyme Biotech Co., Ltd, Nanjing, China) was used for alanine scanning, deletion of H57–M71 and A156-F168, and saturated mutagenesis of BsGT-1. The primers were listed in Supplementary Tables 7–9. After confirmation by Sanger sequencing, the mutants were subjected to protein expression and purification, followed by glycosylation of epothilone B with the sugar donors displayed in Fig. 1.

### In vitro glycosylation reactions and monitorization

The purified protein and mutants were subjected to glycosylation reactions in vitro. For the analytical scale reaction, a mixture of 50 μl containing 50 mM Tris–HCl buffer, 10 mM MgCl_2_, 2 mM sugar donors, 0.4 mM epothilone B (dissolved in DMSO), and 500 μg/mL of each protein was incubated at 37°C. To determine the conversion curves, an aliquot of 50 μl of the reaction mixture was sampled at different time points (0.2 h, 0.4 h, 0.6 h, 0.8 h, 1 h, 2 h, 4 h, 6 h, 8 h, and 10 h). The reaction was terminated with a 3-fold volume of methanol, and the suspensions were centrifuged at 14,000 rpm for 30 min. The samples were subjected to UPLC-PDA and HR-QTOF ESI-MS instruments equipped with a C_18_ column (Thermo Fisher Scientific, C_18_, 250 mm × 4.6 mm, 5 μm). Products were detected by UV absorbance at 249 nm. The mobile phase was isocratic 35% acetonitrile in water at a flow rate of 1 mL/min for 35 min.

For the preparative scale reaction, a 20 mL system containing BsGT-1 (500 μg/mL), 6 mM sugar donors, 1.2 mM EpoB (dissolved in DMSO), 50 mM Tris–HCl (pH 7.5) buffer, and 10 mM MgCl_2_ was used. The reaction was incubated for 12 h at 37°C and stopped by adding the same volume of chilled methanol. After centrifugation at 14,000 rpm and 4°C for 30 min, the supernatant was concentrated by evaporation and lyophilization. The dry powder was dissolved in methanol and then subsequently separated by reversed-phase semipreparative HPLC using a C_18_ column (YMC CO., LTD, Pack Pro C_18_, 250 × 10 mm, 5 μm) with isocratic 40% acetonitrile at a flow rate of 4 mL/min for 30 min. 1D and 2D NMR spectra were recorded on a Bruker Avance III-600 NMR spectrometer (Bruker, Billerica, MA, USA) equipped with CryoprobeTM.

### Kinetic parameters determination

Kinetic parameters of BsGT-1 and its mutants for the glycosylation of epothilone B were measured in a reaction mixture (50 μl) containing 20 μg/mL protein, saturated sugar donors, different concentrations of epothilone B, and 50 mM Tris–HCl buffer (50 mM Tris–HCl, pH 7.5, 10 mM MgCl_2_) at 37°C for 10 min in triplicate. The kinetic values were determined by fitting the Michaelis–Menten curve to the data using the nonlinear regression method (Supplementary Figs. 13–14).

### Modeling and docking analysis

The GT sequences were retrieved from the CAZy database and aligned using MAFFT (Version 7)^64^. The protein structure was remedied by the I-TASSER method with the high-quality crystal structure of BsGT-1 (PDB accession NO: 7BOV) as a reference, and fragment-guided molecular dynamics (FG-MD) simulations and TM-score assessment were used^30,38,65^. The I-TASSER server first identified structural templates from the PDB database by the multiple threading approach LOMETS, with full-length atomic models constructed by iterative template-based fragment assembly simulations^66^. The program AutoDock Vina was used to dock epothilone B and UDP-D-glucose to the 3D structure of BsGT-1^31^. The optimal docking results with high absolute values of ΔG were selected based on program scoring (Supplementary Table 3).

The cocrystal structure of tubulin (β-tubulin subunit)-EpoB with 2.4 Å resolution (7DAE) was used as the model to predict the glycosylation impacts on the antitumor activities of EpoB. AutoDock Vina was also used to dock the representative epothilone B 7-*O*-β-D glucoside (**1a**) to the target cavity of the β-tubulin subunit of microtubules. The optimal docking results with high absolute values of ΔG are shown in Supplementary Table 10.

### Molecular dynamics simulations

Molecular dynamics (MD) simulations of the BsGT-1-EpoB complex were performed using the GROMACS software package^67^. The protein was solvated using the SPC model^68–71^. A cubic box was constructed to perform MD calculations. Water molecules that overlapped with the protein-heavy atoms were removed. The total number of atoms in different solvent systems was ~74,000. To produce a neutral system with 0.1 mol/L ionic concentration, appropriate amounts of Na^+^ and Cl^−^ were added by replacing water molecules with ions randomly. All MD simulations were performed under periodic boundary conditions. The CHARMM27 force field was used to describe the protein. To eliminate steric interference, the steepest energy minimization was performed for every system to give the maximum force below 1000 kJ mol^−1^ nm^−2^. System equilibration was performed at the NVT and NPT ensembles, during which position restraints were applied to protein-heavy atoms. Complete equilibration was assessed by the convergence of the potential energy and the temperatures of the systems. Finally, 100 ns production MD simulations with three replicas were performed in an isothermal−isobaric ensemble. The LINCS algorithm was used to constrain all bonds to hydrogen atoms in the protein, and the SETTLE algorithm was used for the water molecules^72,73^. The particle mesh Ewald (PME) method was used to evaluate the long-range electrostatic interactions^74^. The nonbonded pair list cut-off was 10.0 Å, and the pair list was updated every 10 fs.

### Water solubility, antitumor, and cytotoxicity assays

The water solubility of EpoB and its glycosides was determined using the minimum shake-flask solubility method^26,75^.

By using the MTT colorimetric assay, the antitumor and cytotoxicity of EpoB and its glucosides were tested against the liver carcinoma cell line HepG2 and hepatocyte cell line HL-7702, respectively. The cells were seeded in a 96-well microtiter plate, and the wall-attached cells were treated with varying concentrations of drugs by dilution from 300 μM to 0.1 μM for EpoB glycosides (dissolved in DMSO). After 48 h of incubation with the test compounds, the cultures were treated with MTT (2 mg/mL) solution, and the cells were incubated for an additional 4 h at 37°C until a purple precipitate was visible. The purple formazan crystals were dissolved in DMSO, and the absorbance of each sample was measured at 492 nm using a microplate reader. All MTT assay experiments were performed in triplicate, and IC_50_ values were calculated using GraphPad Prism software (Version 6.01)^26^.

### Immunofluorescence assays

Freshly trypsinized HepG2 cells (1 × 10^4^−1 × 10^5^) were seeded in a 12-well plate and cultivated for 24 h with DMEM (Gibco, Thermo Fisher Scientific, Waltham, MA, USA). Cells were treated with EpoB and **1a** at different concentrations (50 nM, 500 nM, and 5 μM) according to the IC_50_ values for 24 h. The supernatants were removed, and the cells were washed with PBS buffer 2–3 times. The cells were further fixed with 4% paraformaldehyde for 1 h and then permeabilized with 0.2% Triton X-100 (diluted in PBS buffer) for 10 min. Next, the cells were blocked with bovine serum albumin (BSA) for 30 min and incubated with a primary antibody against β-tubulin (1:50, diluted in 2% BSA, β-tubulin Rabbit Monoclonal Antibody, Beyotime Biotechnology) for 1 h at room temperature. After that, the cells were treated with Alexa Fluor 488 goat anti-rabbit IgG (H + L) (secondary antibody, 1:500, diluted in 2% BSA, Beyotime Biotechnology) for another 1 h. The supernatants were removed, the cells were washed, and the nuclei were labeled with DAPI for 10 min. Images were collected using an Olympus BX81 fluorescence microscope system (Olympus, Tokyo, Japan) and a confocal microscope (UltraVIEW^®^VoX).

### Cellular intake assays

HepG2 cells (1 × 10^5^−1 × 10^6^ cells/mL) were cultivated overnight at 37°C and then treated with 5 μM EpoB and glycosides for 1 h. After removing the supernatant, the cells were sequentially rinsed with PBS buffer, trypsinized, and then washed with DMEM. Next, the cells were suspended in acetone, disrupted by ultrasonication, and centrifuged at 12000 rpm for 10 min to remove the debris. The resulting supernatants were dried with nitrogen and then redissolved in 200 μL of methanol. Finally, the compounds were analyzed by HPLC-DAD at 249 nm. Each experiment was conducted in three replicates. The concentrations of EpoB and its glycosides (cellular intake) were calculated based on the linear fitting regression equations of the HPLC peak areas.

### Statistics and reproducibility

Glycosylation, water solubility, MTT, and cellular intake assays were independently replicated three times and the individual data points are reported for each experiment in the main figures.

### Reporting summary

Further information on research design is available in the Nature Research Reporting Summary linked to this article.

## Supplementary information

Supplemental Material

Description of Additional Supplementary Files

Supplementary Data 1

Reporting Summary

## Data Availability

The protein sequences of glycosyltransferases are deposited in GenBank under the following accession numbers: YjiC (AAU40842), BsGT-1 (CUB50191), BaGT (ADP31706), BgGT (SCA85980), BpGT (ARA85718) Details are presented in the Supplementary Table 6. All source data underlying the graphs and charts presented in the main figures are available in the Supplementary Data 1. All other data that support the findings of this study are available from the corresponding authors upon reasonable request.
