# Supplementary material for: Docking-guided rational engineering of a macrolide glycosyltransferase glycodiversifies epothilone B
Source: Commun Biol. 2022 Jan 27;5:100. doi: 10.1038/s42003-022-03047-y (PMC8795383; doi:10.1038/s42003-022-03047-y)

Docking-guided rational engineering of a macrolide glycosyltransferase glycodiversifies epothilone B

Peng Zhang [1], Lijuan Zhang [1], Xukai Jiang [2], Xiao-tong Diao [1], Shuang Li [2], Dan-dan Li [1], Zheng Zhang [1], Junqiang Fang [2], Ya-jie Tang [1], Da-lei Wu [1], Changsheng Wu [1,*], Yue-zhong Li [1,*]

1. State Key Laboratory of Microbial Technology, Institute of Microbial Technology, Shandong University, Qingdao 266237, P.R. China

2. National Glycoengineering Research Center, Shandong Provincial Key Laboratory of Glycochemistry and Glycobiology, Shandong University, Qingdao, Shandong 266237, P.R. China

Yue-zhong Li, lilab@sdu.edu.cn; Tel. (+86) 532 58631539; Fax. (+86) 532 58631539.

Changsheng Wu, wuchangsheng@sdu.edu.cn. Tel. (+86) 532-58631538.

* The corresponding authors.

List of supplementary tables and figures

Supplementary Tables 1-10

Supplementary Figures 1-14

Supplementary Note

Spectra list of new compounds: Supplementary Figures 15-62

Supplementary Table 1 The assignment of the NMR spectra of **1a**, **1b**, **1c** and **1d**.

	**1a**		**1b**		**1c**		**1d**	
**NO.**	**^{1}H NMR**	**^{13}C NMR**	**^{1}H NMR**	**^{13}C NMR**	**^{1}H NMR**	**^{13}C NMR**	**^{1}H NMR**	**^{13}C NMR**
**1**	—	170.5, C	—	170.6, C	—	170.7, C	—	170.6, C
**2**	2.55 (dd, *J* = 15.2, 8.6, 1H); 2.52 (dd, *J* = 15.2, 5.0, 1H)	38.9, CH_2	2.56 (m, 2H)	38.9, CH_2	2.58 (m, 2H)	38.9, CH_2	2.57 (m, 2H)	38.9, CH_2
**3**	4.40 (dd, *J* = 8.5, 5.0 1H)	71.3, CH	4.39 (dd, J = 8.9, 4.7 Hz, 1H)	71.3, CH	4.35 (t, J = 6.8 Hz, 1H)	71.5, CH	4.36 (dd, J = 8.3, 5.3 Hz, 1H)	71.4, CH
**4**	—	53.3, C	—	53.2, C	—	53.1, C	—	53.1, C
**5**	—	219.3, C	—	219.3, C	—	219.1, C	—	219.1, C
**6**	3.54 (m, 1H)	44.9, CH	3.54 (m, 1H)	45.1, CH	3.55 (m, 1H)	46.0, CH	3.54 (m, 1H)	45.9, CH
**7**	3.83 (m, 1H)	83.4, CH	3.84 (m, 1H)	83.7, CH	3.87 (m, 1H)	83.4, CH	3.88 (m, 1H)	83.0, CH
**8**	1.68 (m, 1H)	37.6, CH	1.67 (m, 1H)	37.5, CH	1.58 (m, 1H)	37.1, CH	1.59 (m, 1H)	37.1, CH
**9**	1.61 (m, 1H); 1.34 (m, 1H)	30.1, CH_2	1.33 (m, 2H)	30.1, CH_2	1.31 (m, 2H)	29.8, CH_2	1.30 (m, 2H)	29.7, CH_2
**10**	1.65 (m, 1H); 1.40 (m, 1H)	23.0, CH_2	1.63 (m, 1H); 1.39 (m, 1H)	23.1, CH_2	1.64 (m, 1H); 1.36 (m, 1H)	23.1, CH_2	1.65 (m, 1H); 1.35 (m, 1H)	22.9, CH2
**11**	1.67 (m, 1H); 1.51 (m, 1H)	31.0, CH_2	1.66 (m, 1H); 1.51 (m, 1H)	31.0, CH_2	1.65 (m, 1H); 1.52 (m, 1H)	31.0, CH_2	1.65 (m, 1H); 1.51 (m, 1H)	30.9, CH_2
**12**	—	61.1, C	—	61.2, C	—	61.3, C	—	61.2, C
**13**	2.91 (t, *J* = 6.0 Hz, 1H)	61.6, CH	2.91 (dd, J = 7.2, 5.4 Hz, 1H)	61.6, CH	2.92 (dd, J = 7.7, 5.1 Hz, 1H)	61.7, CH	2.91 (dd, J = 7.2, 5.4 Hz, 1H)	61.6, CH
**14**	2.07 (m, 2H)	32.1, CH_2	2.08 (m, 2H)	32.1, CH_2	2.08 (m, 2H)	32.2, CH_2	2.08 (m, 2H)	32.1, CH_2
**15**	5.47 (dd, *J* = 6.0, 4.2, 1H)	76.5, CH	5.47 (dd, J = 6.8, 3.3 Hz, 1H)	76.5, CH	5.43 (dd, J = 7.3, 3.2 Hz, 1H)	76.4, CH	5.44 (dd, J = 6.7, 3.6 Hz, 1H)	76.4, CH
**16**	—	137.8, C	—	137.8, C	—	138.0, C	—	137.9, C
**17**	6.64 (s, 1H)	119.0, CH	6.63 (s, 1H)	119.0, CH	6.64 (s, 1H)	118.9, CH	6.64 (s, 1H)	119.0, CH
**18**	—	151.8, C	—	151.8, C	—	151.8, C	—	151.8, C
**19**	7.27 (s, 1H)	116.3, CH	7.27 (s, 1H)	116.4, CH	7.27 (s, 1H)	116.3, CH	7.28 (s, 1H)	116.3, CH
**20**	—	165.6, C	—	165.6, C	—	165.7, C	—	165.7, C
**21**	2.71 (s, 3H)	17.3, CH_3	2.71 (s, 3H)	17.3, CH_3	2.72 (s, 3H)	17.3, CH_3	2.72 (s, 3H)	17.3, CH_3
**22**	1.06 (s, 3H)	19.2, CH_3	1.06 (s, 3H)	19.3, CH_3	1.06 (s, 3H)	19.7, CH_3	1.06 (s, 3H)	19.5, CH_3
**23**	1.30 (s, 3H)	20.5, CH_3	1.31 (s, 3H)	21.0, CH_3	1.33 (s, 3H)	21.0, CH_3	1.32 (s, 3H)	21.0, CH_3
**24**	1.31 (d, *J* = 7.2 Hz, 3H)	15.1, CH_3	1.31 (d, J = 7.2, 3H)	15.1, CH_3	1.35 (d, J = 7.2, 3H)	15.9, CH_3	1.35 (d, J = 7.2, 3H)	15.8, CH_3
**25**	1.07 (d, *J* = 7.2 Hz, 3H)	16.5, CH_3	1.07 (d, J = 7.2, 3H)	16.6, CH3	1.06 (d, J = 7.2, 3H)	16.8, CH_3	1.05 (d, J = 7.2, 3H)	16.7, CH_3
**26**	2.10 (d, *J* = 1.2, 3H)	14.3, CH_3	2.10 (s, 3H)	14.2, CH_3	2.10 (s, 3H)	14.3, CH_3	2.10 (s, 3H)	14.2, CH_3
**27**	1.30 (s, 3H)	21.0, CH_3	1.30 (s, 3H)	20.6, CH_3	1.30 (s, 3H)	20.8, CH_3	1.31 (s, 3H)	20.7, CH_3
**1′**	4.48(d, *J* = 7.8 Hz, 1H)	103.3, CH	4.55 (d, J = 7.8 Hz, 1H)	103.0, CH	4.65 (d, J = 7.8 Hz, 1H)	101.4, CH	4.66 (d, J = 7.2 Hz, 1H)	101.1, CH
**2′**	3.24 (t, *J* = 7.2 Hz, 1H)	74.4, CH	3.43 (t, J = 7.8 Hz, 1H)	73.8, CH	3.50 (t, J = 7.8 Hz, 1H)	82.0, CH	3.47 (t, J = 7.8 Hz, 1H)	82.5, CH
**3′**	3.40 (t, *J* = 9.0, 1H)	76.9, CH	3.59 (t, J = 9.0, 1H)	86.8, CH	3.60 (t, J = 6.8, 1H)	76.9, CH	3.60 (t, J = 9.0, 1H)	76.8, CH
**4′**	3.33 (m, 1H)	70.5, CH	3.46 (t, J = 9.0, 1H)	68.8, CH	3.38 (t, J = 9.0, 1H)	70.4, CH	3.37 (m, 1H)	70.4, CH
**5′**	3.29 (m, 1H)	76.3, CH	3.32 (m, 1H)	76.0, CH	3.30 (m, 1H)	76.8, CH	3.29 (m, 1H)	76.1, CH
**6′**	3.87 (d, *J* = 12.0, 1H); 3.71 (dd, *J* = 12.0, 4.8, 1H)	61.5, CH_2	3.89 (d, J = 11.4, 2.4 Hz, 1H); 3.74 (dd, J = 11.4, 5.4 Hz, 1H)	61.4, CH_2	3.90 (d, J = 11.4, 2.4 Hz, 1H); 3.73 (dd, J = 11.4, 5.4 Hz, 1H)	61.4, CH_2	3.93 (m, 1H); 3.71 (dd, J = 11.7, 5.4 Hz, 1H)	61.4, CH_2
**1′′**			4.60 (d, J = 7.2 Hz, 1H)	103.9, CH	4.62 (d, J = 7.8 Hz, 1H)	104.3, CH	4.64 (d, J = 7.8 Hz, 1H)	104.4, CH
**2′′**			3.29 (t, J = 7.8 Hz, 1H)	70.2, CH	3.27 (t, J = 7.8 Hz, 1H)	74.8, CH	3.33 (m, 1H)	74.6, CH
**3′′**			3.29 (t, J = 7.8 Hz, 1H)	74.2, CH	3.32 (t, J = 7.8 Hz, 1H)	70.3, CH	3.56 (t, J = 8.4 Hz, 1H)	74.8, CH
**4′′**			3.41 (t, J = 9.0 Hz, 1H)	76.4, CH	3.29 (t, J = 9.0 Hz, 1H)	76.1, CH	3.60 (t, J = 9.0 Hz, 1H)	79.6, CH
**5′′**			3.35 (m, 1H)	76.8, CH	3.30 (m, 1H)	76.5, CH	3.44 (m, 1H)	75.5, CH
**6′′**			3.91 (d, J = 11.4, 2.4 Hz, 1H); 3.66 (dd, J = 11.4, 5.4, 1H)	61.3, CH_2	3.90 (d, J = 11.4, 2.4 Hz, 1H); 3.73 (dd, J = 11.4, 5.4 Hz, 1H)	61.6, CH_2	3.93 (m, 1H); 3.88 (m, 1H)	60.9, CH_2
**1′′′**							4.42 (d, J = 7.8 Hz, 1H)	103.3, CH
**2′′′**							3.24 (t, J = 8.4 Hz, 1H)	73.5, CH
**3′′′**							3.33 (t, J = 7.8 Hz, 1H)	74.6, CH
**4′′′**							3.32 (m, 1H)	70.0, CH
**5′′′**							3.34 (m, 1H)	76.7, CH
**6′′′**							3.88 (m, 1H); 3.67 (dd, J = 11.9, 5.6 Hz, 1H)	61.0, CH_2

Supplementary Table 2 The assignment of the NMR spectra of **1e**, **1h** and **1i**.

	**1e**		**1h**		**1i**	
**No.**	**^{1}H NMR**	**^{13}C NMR**	**^{1}H NMR**	**^{13}C NMR**	**^{1}H NMR**	**^{13}C NMR**
**1**	—	170.4, C	—	170.5, C	—	170.5, C
**2**	2.58 (dd, J = 15.2, 8.6, 1H); 2.52 (dd, J = 15.2, 5.0, 1H)	39.0, CH2	2.56 (dd, *J* = 15.2, 9.0, 1H); 2.49 (dd, *J* = 15.2, 5.0, 1H)	39.0, CH2	2.57 (dd, J = 15.2, 8.6, 1H); 2.53 (dd, J = 15.2, 5.0, 1H)	38.9, CH2
**3**	4.45 (dd, J = 8.5, 5.0 1H)	71.3, C	4.42 (dd, *J* = 8.4, 4.8 1H)	71.3, C	4.37 (dd, J = 8.5, 5.0 1H)	71.3, CH
**4**	—	53.2, C	—	53.2, C	—	53.3, C
**5**	—	219.5, C	—	?	—	219.4, C
**6**	3.48 (m, 1H)	43.9, CH	3.46 (m, 1H)	43.7, CH	3.52 (m, 1H)	44.9, CH
**7**	3.81 (dd, J = 3.8, 2.4, 1H)	82.1, CH	3.80(dd, *J* = 4.8, 2.4, 1H)	82.0, CH	3.82 (dd, J = 5.4, 1.8, 1H)	83.6, CH
**8**	1.79 (m, 1H)	38.4, CH	1.81 (m, 1H)	38.5, CH	1.68 (m, 1H)	37.7, CH
**9**	1.53 (m, 1H); 1.34 (m, 1H)	29.7, CH2	1.53 (m, 1H); 1.33 (m, 1H)	29.7, CH2	1.60 (m, 1H); 1.34 (m, 1H)	30.3, CH2
**10**	1.65 (m, 1H); 1.44 (m, 1H)	22.7, CH2	1.64 (m, 1H); 1.44 (m, 1H)	22.8, CH2	1.63 (m, 1H); 1.39 (m, 1H)	23.1, CH2
**11**	1.65 (m, 1H); 1.54 (m, 1H)	30.5, CH2	1.65 (m, 1H); 1.52 (m, 1H)	30.6, CH2	1.65 (m, 1H); 1.52 (m, 1H)	31.1, CH2
**12**	—	60.8, C	—	60.9, C	—	61.2, C
**13**	2.88 (t, J = 6.0 Hz, 1H)	61.1, CH	2.87 (t, *J* = 6.0 Hz, 1H)	61.3, CH	2.91 (t, J = 6.0 Hz, 1H)	61.7, CH
**14**	2.10 (m, 1H); 2.00 (m, 1H)	31.6, CH2	2.04 (m, 2H)	31.7, CH2	2.08 (m, 2H)	32.1, CH2
**15**	5.48 (dd, J = 6.0, 4.2, 1H)	76.4, CH	5.48 (dd, *J* = 6.0, 4.2, 1H)	76.5, CH	5.46 (dd, J = 6.0, 4.2, 1H)	76.5, CH
**16**	—	137.5, C	—	137.6, C	—	137.9, C
**17**	6.63 (s, 1H)	118.9, CH	6.62 (s, 1H)	119.0, CH	6.63 (s, 1H)	119.0, CH
**18**	—	151.7, C	—	151.7, C	—	151.7, C
**19**	7.27 (s, 1H)	116.3, CH	7.26 (s, 1H)	116.3, CH	7.27 (s, 1H)	116.4, CH
**20**	—	165.7, C	—	165.7, C	—	165.7, C
**21**	2.71 (s, 3H)	17.3, CH3	2.70 (s, 3H)	17.3, CH3	2.71 (s, 3H)	17.3, CH3
**22**	1.06 (s, 3H)	19.2, CH3	1.04 (s, 3H)	19.0, CH3	1.04 (s, 3H)	19.2, CH3
**23**	1.30(s, 3H)	20.8, CH3	1.29 (s, 3H)	20.8, CH3	1.31 (s, 3H)	20.7, CH3
**24**	1.18 (d, J = 7.2 Hz, 3H)	14.3, CH3	1.17 (d, *J* = 7.2 Hz, 3H)	14.4, CH3	1.31 (d, J = 7.2 Hz, 3H)	15.1, CH3
**25**	1.01 (d, J = 7.2 Hz, 3H)	16.0, CH3	1.01 (d, *J* = 7.2 Hz, 3H)	15.9, CH3	1.05 (d, J = 7.2 Hz, 3H)	16.5, CH3
**26**	2.09 (d, J = 1.2, 3H)	14.4, CH3	2.08 (d, *J* = 1.2, 3H)	14.4, CH3	2.10 (d, J = 1.2, 3H)	14.2, CH3
**27**	1.29 (s, 3H)	20.8, CH3	1.28 (s, 3H)	20.1, CH3	1.30 (s, 3H)	21.0, CH3
**1’**	4.65 (d, J = 8.4 Hz, 1H)	101.6, CH	4.69 (d, *J* = 8.4 Hz, 1H)	101.4, CH	4.42 (d, J = 7.2 Hz, 1H)	104.1, CH
**2’**	3.72 (m, 1H)	56.4, CH	3.76 (dd, *J* = 9.6, 7.2, 1H)	56.4, CH	3.56 (dd, J = 9.6, 7.2 Hz, 1H)	71.9, CH
**3’**	3.48 (t, J = 8.4, 1H)	75.0, CH	3.54 (dd, *J* = 10.2, 8.4, 1H)	74.7, CH	3.52 (m, 1H)	73.8, CH
**4’**	3.36 (t, J = 9.0, 1H)	70.9, CH	3.36 (t, *J* = 9.0, 1H)	70.9, CH	3.87 (brd, 1H)	68.7, CH
**5’**	3.29 (m, 1H)	76.2, CH	3.29 (m, 1H)	76.3, CH	3.51 (m, 1H)	74.9, CH
**6’**	3.89 (dd, J = 11.8, 2.4, 1H); 3.72 (m, 1H)	61.6, CH2	3.89 (dd, *J* = 11.8, 2.4, 1H); 3.71 (dd, *J* = 12.0, 6.0, 1H)	61.5, CH2	3.78 (dd, J = 15.4, 6.6, 1H); 3.72 (dd, J = 14.8, 6.0, 1H)	60.7, CH2
**7’**	—	172.3, C	—	169.0, C		
**8’**	1.97 (s, 3H)	21.7, CH3	3.90 (d, *J* = 8.4, 2H)	51.9, CH2		

Supplementary Table 3 The docking details of BsGT-1 with two ligands (Epothilone B and UDPG), The docking model with superior parameters was selected.

Model	BsGT-1-Epothilone B			BsGT-1-UDPG		
	ΔG (kcal/mol)	distance from best mode		ΔG (kcal/mol)	distance from best mode	
		rmsd l.b.	rmsd u.b.		rmsd l.b.	rmsd u.b.
1	-8.8	0.000	0.000	-9.8	0.000	0.000
2	-8.8	4.112	8.656	-9.6	2.269	3.026
3	-8.8	4.921	7.785	-9.6	2.444	3.617
4	-8.7	3.979	7.943	-9.6	3.602	9.858
5	-8.7	3.695	7.945	-9.2	3.495	10.627
6	-8.6	4.357	6.989	-9.2	3.496	10.325
7	-8.5	4.180	8.974	-9.1	3.491	10.345
8	-8.1	2.478	3.125	-9.1	3.538	10.526
9	-7.9	2.397	3.042	-8.7	2.680	5.205

Standard error (SE) for ΔG (kcal/mol) of BsGT-1-Epothilone B model and BsGT-1-UDPG model were 0.11 and 0.12

Supplementary Table 4 The predicted residues having hydrogen bonds or Van der Waal's forces interactions with ligands.

Ligands	Hydrogen bonds	Van der Waals forces
Epothilone B (EpoB)	**H16, N140, E317**	N61, I62, D63, M71, K73, L80, F107, V108, **S128**, **S129**, Y130, D142, K146, **K210**, **F231**, **S277**, **E301**, M315, Y316, **Q318**, L320
UDPG (**2a**)	**H16**, N18, **N140**, R207, S226, T229, S255, V278, Q280, H293, M296, N297, S298, **E317**, **Q318**	**S128**, **S129**, **K210**, **F231**, **S277, E301**
Epothilone B / UDPG	**H16, N140, E317, Q318**	**S128, S129, K210, F231, S277**

Supplementary Table 5 The kinetic parameters of wild-type (WT) BsGT-1 and its mutants to produce epothilone B (EpoB) glycosides.

**Enzyme**	**Substrates Conversion**	**Sugar Donors**	***K*m (μM)**	**k_{cat} (min^{-1})**	**k_{cat}/*K*m (min^{-1} mM^{-1})**
WT	EpoB (CNV to **1a**)	**2a**	49.76±2.37	28.23±0.53	567.32
WT	**1a** (CNV to **1b**)	**2a**	260.41±25.65	1.61±0.05	6.18
WT	**1a** (CNV to **1c**)	**2a**	812.18±42.28	0.78±0.06	0.96
WT	EpoB (CNV to **1e**)	**2e**	437.15±39.23	1.52±0.19	3.48
WT	EpoB (CNV to **1h**)	**2h**	692.10±62.41	1.05±0.03	1.52
WT	EpoB (CNV to **1i**)	**2i**	378.01±25.18	0.87±0.02	2.3
I62A	EpoB (CNV to **1a**)	**2a**	43.65±4.16	23.85±1.38	546.39
I62A	**1a** (CNV to **1b**)	**2a**	70.96±6.39	12.42±0.5	175.03
I62A	**1a** (CNV to **1c**)	**2a**	394.51±28.56	1.99±0.04	5.04
I62A	**1c** (CNV to **1d**)	**2a**	449.01±23.52	1.19±0.02	2.65
F231S	EpoB (CNV to **1h)**	**2h**	126.69±12.88	4.34±0.2	34.26
M296A	EpoB (CNV to **1i**)	**2i**	146.97±16.99	3.33±0.18	22.66
Q318C	EpoB (CNV to **1e**)	**2e**	95.88±7.98	9.87±0.22	102.94

CNV to, Convert to

Supplementary Table 6 Basic information about the enzymes in this paper.

Enzyme	Genbank number	Sources of strains	PDB ID
YjiC	AAU40842	*Bacillus licheniformis* DSM 13	-
*BsGT-1	CUB50191	*Bacillus subtilis* JRS11	7BOV
*BaGT	ADP31706	*Bacillus atrophaeus* 1942	-
*BgGT	SCA85980	*Bacillus glycinifermentans* BGLY 2157	-
*BpGT	ARA85718	*Bacillus paralicheniformis* MDJK30	-
OleD	ABA42119	*Streptomyces antibioticus* ATCC 11891 DSM 40868	2IYF
OleI	ABA42118	*Streptomyces antibioticus* DSM 40868 ATCC 11891	2IYA
VvGT1	AAB81683	*Vitis vinifera*	2C1X
UGT85H2	ABE87250	*Medicago truncatula*	2PQ6
UGT71H1	AAW56092	*Medicago truncatula*	2ACV

GTs identity (%)	YjiC	BsGT-1	BgGT	BaGT
BsGT-1	56.1			
BgGT	75.2	57.3		
BaGT	54.3	77.0	55.1	
BpGT	94.4	55.6	76.0	54.6

*BsGT-1：Bacillus subtilis glycosyltransferase-1;

*BaGT：Bacillus atrophaeus glycosyltransferase;

*BgGT：Bacillus glycinifermentans glycosyltransferase;

*BpGT：Bacillus paralicheniformis glycosyltransferase;

Supplementary Table 7 The primers of 35 alanine-scanning mutants

Primers	Sequence (5' to 3')
BsGT-1-H16A-F	ATACGGAgccGTCAATCCTACGCTTGCTTTAGTAGA
BsGT-1-H16A-R	GATTGACggcTCCGTATGCCGGGATATTGATC
BsGT-1-N18A-F	ATGTCgcaCCTACGCTTGCTTTAGTAGAGAAGC
BsGT-1-N18A-R	AAGCGTAGGtgcGACATGTCCGTACGCCGGGA
BsGT-1-N61A-F	TCCTTGgcaATTGATCCTAAGCAAATCAGGGAG
BsGT-1-N61A-R	GGATCAATtgcCAAGGATGTATGATAGATCAATGCTTC
BsGT-1-I62A-F	CCTTGAATgcaGATCCTAAGCAAATCAGGGAGATG
BsGT-1-I62A-R	AGGATCtgcATTCAAGGATGTATGATAGATCAATGC
BsGT-1-D63A-F	gcaCCTAAGCAAATCAGGGAGATGATGGAAAA
BsGT-1-D63A-R	CTGATTTGCTTAGGtgcAATATTCAAGGATGTATGATAGATCAATGC
BsGT-1-M71A-F	GGAGATTgccGAAAAGAATGACGCGCCCCTCA
BsGT-1-M71A-R	TCTTTTCggcAATCTCCCTGATTTGCTTAGGATC
BsGT-1-K73A-F	GATGGAAgcaAATGACGCGCCCCTCAGCCTTT
BsGT-1-K73A-R	CGTCATTtgcTTCCATCATCTCCCTGATTTGC
BsGT-1-L80A-F	CTCAGCgccTTGAAAGAATCACTCAGCATTCTGC
BsGT-1-L80A-R	TCTTTCAAggcGCTGAGGGGCGCGTCATTCTT
BsGT-1-F107A-F	CTATGACgccGTTGCGCTGGCTGGTAAATTGT
BsGT-1-F107A-R	GCGCAACggcGTCATAGATGATCAGATCAGGCTGA
BsGT-1-V108A-F	ATGACTTTgcaGCGCTGGCTGGTAAATTGTTT
BsGT-1-V108A-R	CAGCGCtgcAAAGTCATAGATGATCAGATCAGGCT
BsGT-1-S128A-F	GCTCTGTgcaTCATATGCCCAAAATGAATCCTT
BsGT-1-S128A-R	CATATGAtgcACAGAGCTTAATGACCGGAACATT
BsGT-1-S129A-F	TTCGgccTATGCCCAAAATGAATCCTTTCAGT
BsGT-1-S129A-R	TTTGGGCATAggcCGAACAGAGCTTAATGACCGG
BsGT-1-Y130A-F	TCAgccGCCCAAAATGAATCCTTTCAGTTAGG
BsGT-1-Y130A-R	TCATTTTGGGCggcTGACGAACAGAGCTTAATGACCG
BsGT-1-N140A-F	GTTAGGAgcaGAAGACATGCTGAAAAAAATAAGAGAA
BsGT-1-N140A-R	TGTCTTCtgcTCCTAACTGAAAGGATTCATTTTGG
BsGT-1-D142A-F	GAAATGAAgcaATGCTGAAAAAAATAAGAGAAGCAGA
BsGT-1-D142A-R	CAGCATtgcTTCATTTCCTAACTGAAAGGATTCAT
BsGT-1-K146A-F	GCTGAAAgcaATAAGAGAAGCAGAGGCTGAATTTAA
BsGT-1-K146A-R	CTCTTATtgcTTTCAGCATGTCTTCATTTCCTAAC
BsGT-1-R207A-F	CGGAGAAgcaAAGGAAAAAGAAAGCCTGTTGATT
BsGT-1-R207A-R	TTTCCTTtgcTTCTCCGAGAGAGGGGCCGACA
BsGT-1-K210A-F	GAAGGAAgcaGAAAGCCTGTTGATTGACAAGGA
BsGT-1-K210A-R	GGCTTTCtgcTTCCTTCCGTTCTCCGAGAGAG
BsGT-1-S226A-F	GCTGATTgcaTTGGGTACGGCGTTTAACGCAT
BsGT-1-S226A-R	TACCCAAtgcAATCAGCATAAGCGGGCGATCA
BsGT-1-T229A-F	TTTGGGTgcaGCGTTTAACGCATGGCCGGAAT
BsGT-1-T229A-R	TAAACGCtgcACCCAAAGAAATCAGCATAAGCG
BsGT-1-F231A-F	TACGGCGgcaAACGCATGGCCGGAATTTTACA

BsGT-1-F231A-R	ATGCGTTtgcCGCCGTACCCAAAGAAATCAGC
BsGT-1-S255A-F	GATCATGgcaGTTGGGAAAACGATTGATCCAG
BsGT-1-S255A-R	TCCCAACtgcCATGATCACTTGCCATGAAGAATC
BsGT-1-S277A-F	TTCGCCAAgcaGTGCCGCAGCTTGAGGTGTTA
BsGT-1-S277A-R	CGGCACtgcTTGGCGAATGGTAAAGTTAGCAG
BsGT-1-V278A-F	AAAGTgcaCCGCAGCTTGAGGTGTTAGAGAAA
BsGT-1-V278A-R	AAGCTGCGGtgcACTTTGGCGAATGGTAAAGTTAGC
BsGT-1-Q280A-F	AAAGTGTGCCGgcaCTTGAGGTGTTAGAGAAAGCTGATTT
BsGT-1-Q280A-R	AAGtgcCGGCACACTTTGGCGAATGGTAAAGT
BsGT-1-H293A-F	TGTTCATCTCTgcaGGCGGGATGAACAGTACGATG
BsGT-1-H293A-R	GCCtgcAGAGATGAACAAATCAGCTTTCTCTAA
BsGT-1-M296A-F	GGGgcaAACAGTACGATGGAAGCGATGAACGC
BsGT-1-M296A-R	ATCGTACTGTTtgcCCCGCCATGAGAGATGAACA
BsGT-1-N297A-F	GGGATGgcaAGTACGATGGAAGCGATGAACGC
BsGT-1-N297A-R	ATCGTACTtgcCATCCCGCCATGAGAGATGAA
BsGT-1-S298A-F	GATGAACgcaACGATGGAAGCGATGAACGCAG
BsGT-1-S298A-R	CCATCGTtgcGTTCATCCCGCCATGAGAGATG
BsGT-1-E301A-F	TACGATGgcaGCGATGAACGCAGGTGTGCCGC
BsGT-1-E301A-R	TCATCGCtgcCATCGTACTGTTCATCCCGCCA
BsGT-1-M315A-F	TCCGCAAgcaTATGAGCAAGAGCTCACTGCAAA
BsGT-1-M315A-R	GCTCATAtgcTTGCGGAATGACGACAAGCGGC
BsGT-1-Y316A-F	GCAAATGgcaGAGCAAGAGCTCACTGCAAATCG
BsGT-1-Y316A-R	CTTGCTCtgcCATTTGCGGAATGACGACAAGC
BsGT-1-E317A-F	AATGTATgccCAAGAGCTCACTGCAAATCGGG
BsGT-1-E317A-R	GCTCTTGggcATACATTTGCGGAATGACGACA
BsGT-1-Q318A-F	TGAGnnnGAGCTCACTGCAAATCGGGTTGATG
BsGT-1-Q318A-R	CAGTGAGCTCnnnCTCATACATTTGCGGAATGACG
BsGT-1-L320A-F	GCAAGAGgcaACTGCAAATCGGGTTGATGAAT
BsGT-1-L320A-R	TTGCAGTtgcCTCTTGCTCATACATTTGCGGA
BsGT-1-ΔH57-M71-F	GAAGCATTGATCtatGAAAAGAATGACGCGCCCC
BsGT-1-ΔH57-M71-R	TCataGATCAATGCTTCTCCACCGGCTTGCTG
BsGT-1-ΔA156-F168-F	GGCTGAATTTaaaGAACAGTTAGCTGTGCCGGAA
BsGT-1-ΔA156-F168-R	GTTCtttAAATTCAGCCTCTGCTTCTCTTATT

Supplementary Table 8 The primers of saturated mutants

Primer	Sequence (5' to 3')
BsGT-1-I62SM-F	CCTTGAATnnsGATCCTAAGCAAATCAGGGAGATG
BsGT-1-I62SM-R	AGGATCsnnATTCAAGGATGTATGATAGATCAATGC
BsGT-1-F231SM-F	TACGGCGnnsAACGCATGGCCGGAATTTTACA
BsGT-1-F231SM-R	TACGGCGsnnAACGCATGGCCGGAATTTTACA
BsGT-1-M296SM-F	TGGCGGGnnsAACAGTACGATGGAAGCGATGAA
BsGT-1-M296SM-R	TACTGTTsnnCCCGCCATGAGAGATGAACAAA
BsGT-1-E317SM-F	AATGTATnnsCAAGAGCTCACTGCAAATCGGG
BsGT-1-E317SM-R	GCTCTTGsnnATACATTTGCGGAATGACGACA
BsGT-1-Q318SM-F	TGAGnnsGAGCTCACTGCAAATCGGGTTGATG
BsGT-1-Q318SM-R	CAGTGAGCTCsnnCTCATACATTTGCGGAATGACG

Supplementary Table 9 The primers of site-directed mutants of YjiC, BaGT, BgGT and BpGT

**Primers**	**Sequence (5' to 3')**
YjiC-I62A-F	TAAATgcaGATCCGCAGCAAATTCGGGAGCTG
YjiC-I62A-R	CTGCGGATCtgcATTTAAAGTTGAGCGGTAGTTGAGC
BaGT-V62A-F	TATTTCGgcaGATCCTCAGCAAATTAAAGAATTGAT
BaGT-V62A-R	GAGGATCtgcCGAAATAGTTGTTTCATATAAAAGCGG
BgGT-I62A-F	TAAATgcaGATCCTCGCCAAATTCGGGAAGTG
BgGT-I62A-R	GCGAGGATCtgcATTTAAGGTTGAACGGTATTGAAGC
BpGT-I62A-F	TAAATgcaGATCCGCAGCAAATTCGGGAGCTG
BpGT-I62A-R	CTGCGGATCtgcATTTAAAGTTGAGCGGTAGTTGAGC
YjiC-F236S-F	TAGGGACGGCGtcaAATGCCTGGCCGGAATTT
YjiC-F236S-R	ATTtgaCGCCGTCCCTAAAGATATCAGCATGA
BaGT-F232S-F	AACGGCCtcaAACGCCTGGCCGGAATTCTATC
BaGT-F232S-R	AGGCGTTtgaGGCCGTTCCCAGTGAAATCAGC
BgGT-F234S-F	GACAGCCtcaAATGCCTGGCCTGAGTTCTATG
BgGT-F234S-R	AGGCATTtgaGGCTGTCCCCAGTGAAATCAGC
BpGT-F236S-F	TAGGGACGGCGtcaAATGCCTGGCCGGAATTT
BpGT-F236S-R	ATTtgaCGCCGTCCCTAAAGAAATCAGCATGA
YjiC-M301A-F	TGGGGGTgcaAACAGTACGATGGAAGGGTTGAA
YjiC-M301A-R	TACTGTTtgcACCCCCATGGGTGATGAACAGC
BaGT-M297A-F	TCACGGAGGAgcaAACAGCACGATGGAAGCGAT
BaGT-M297A-R	TGTTtgcTCCTCCGTGAGAAATAAATACATCA
BgGT-M299A-F	GGGAgcaAACAGTACGATGGAAGCATTGAATG
BgGT-M299A-R	TCGTACTGTTtgcTCCCCCGTGGCTGATGAAC
BpGT-M301A-F	TGGGGGTgcaAACAGTACGATGGAAGGATTGAATG
BpGT-M301A-R	TACTGTTtgcACCCCCATGGGTGATGAAAAGC
YjiC-Q323C-F	GCCTGAAtgcGAAATCACTGCCCGCCGCGTCG
YjiC-Q323C-R	TGATTTCgcaTTCAGGCATTTGCGGAACGGCA
BaGT-Q319C-F	GCATGAAtgcGAACTCACGGCCCAGCGTGTTG
BaGT-Q319C-R	TGAGTTCgcaTTCATGCATTTGCGGAATCACT
BgGT-Q321C-F	GCCGGAAtgcGAAATCACGGCCCGCCGCGTGG
BgGT-Q321C-R	TGATTTCgcaTTCCGGCATTTGCGGAATGACG
BpGT-Q323C-F	GCCTGAAtgcGAAATCACTGCCCGCCGCGTCG
BpGT-Q323C-R	TGATTTCgcaTTCAGGCATTTGCGGGACGGCA

Supplementary Table 10 The docking details of Tubulin (β-tubulin subunit) with the representative mono-glucosylated compound, epothilone B 7-O-β-D glucoside (**1a**). The superior docking model was selected.

Model	Tubulin (β-tubulin subunit)-**1a**		
	ΔG (kcal/mol)	distance from best mode	
		rmsd l.b.	rmsd u.b.
1	-6.5	0.000	0.000
2	-6.3	4.647	10.976
3	-6.2	8.798	15.220
4	-6.1	7.141	13.954
5	-6.1	4.761	10.937
6	-5.9	10.824	14.842
7	-5.8	6.974	12.834
8	-5.6	5.114	9.332
9	-5.6	8.219	11.585

Standard error (SE) for ΔG (kcal/mol) of Tubulin (β-tubulin subunit)-1a model was 0.1

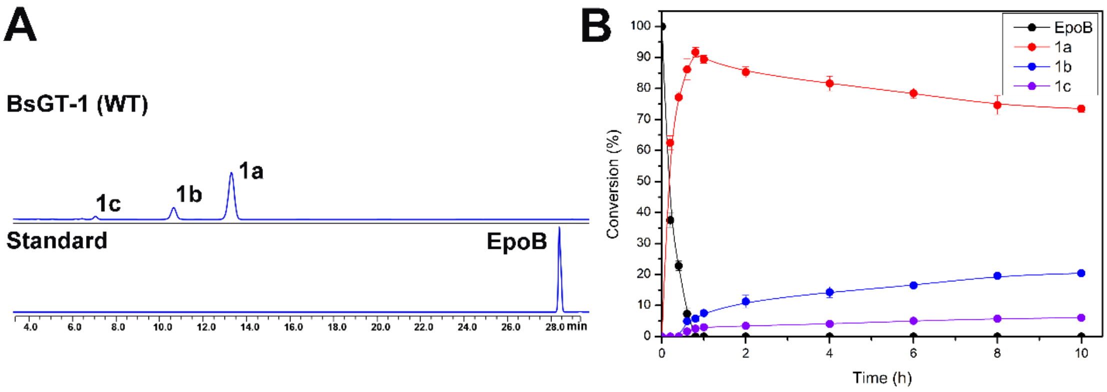

Supplementary Fig. 1 BsGT-1 could glycosylate EpoB using UDPG as sugar donor. (A) The HPLC detection of the conversion from EpoB to its glucosides by wild-type BsGT-1. (B) The time-course production of EpoB glucoside **1a**, **1b** and **1c** by the wild-type enzyme within 10 h of incubation.

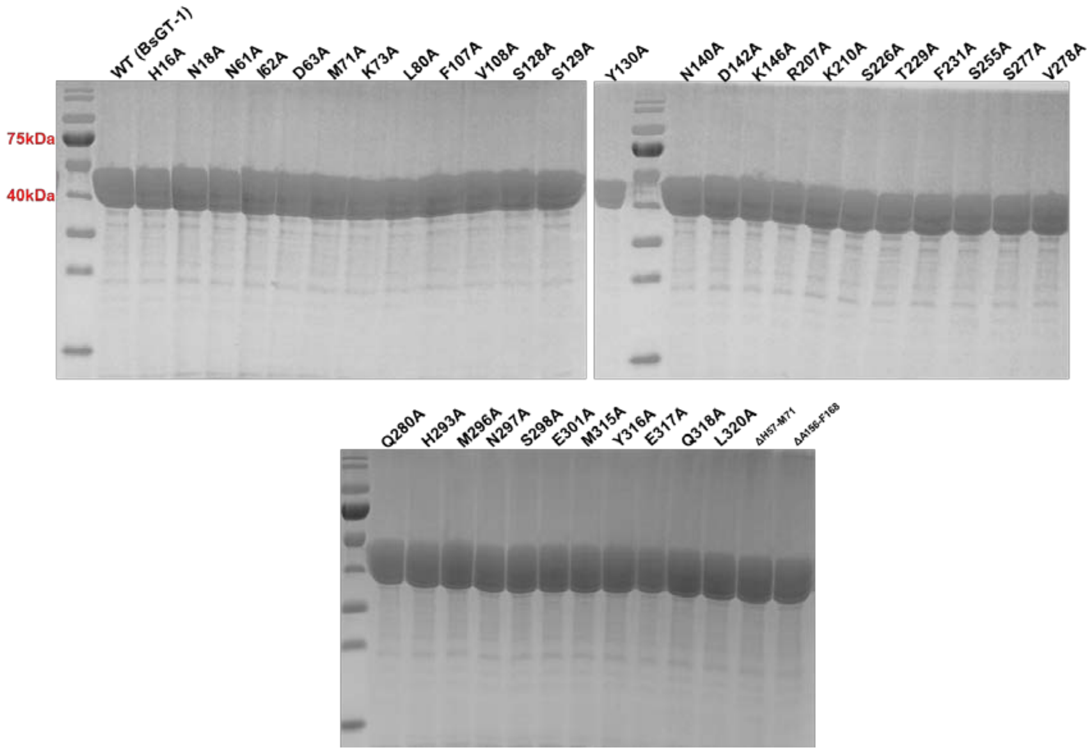

Supplementary Fig. 2 The purified proteins of 35 alanine-scanning mutants and the ΔH57-M71 and ΔA156-F168 mutants of BsGT-1.

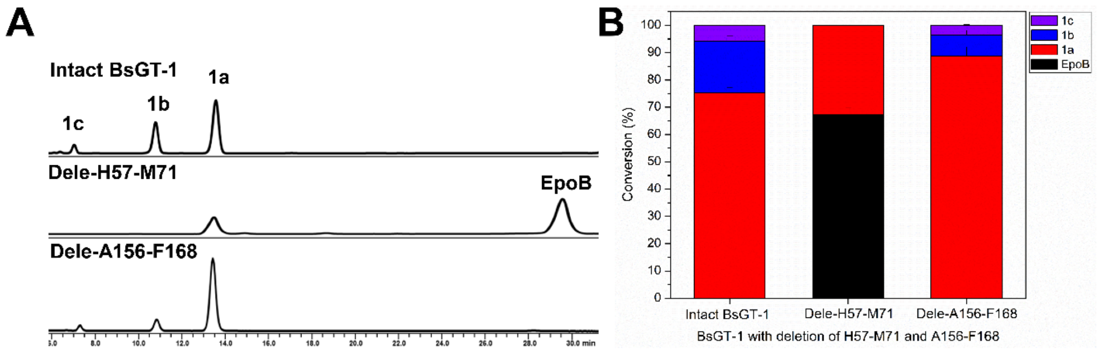

Supplementary Fig. 3 (A). The HPLC detection of the products from the catalysis of complete BsGT-1, and its incomplete proteins with deletion of residues H57-M71 and A156-F168. (B). The conversion comparison (mean ± SD) of complete BsGT-1, and its incomplete proteins with deletion of residues H57-M71 and A156-F168.

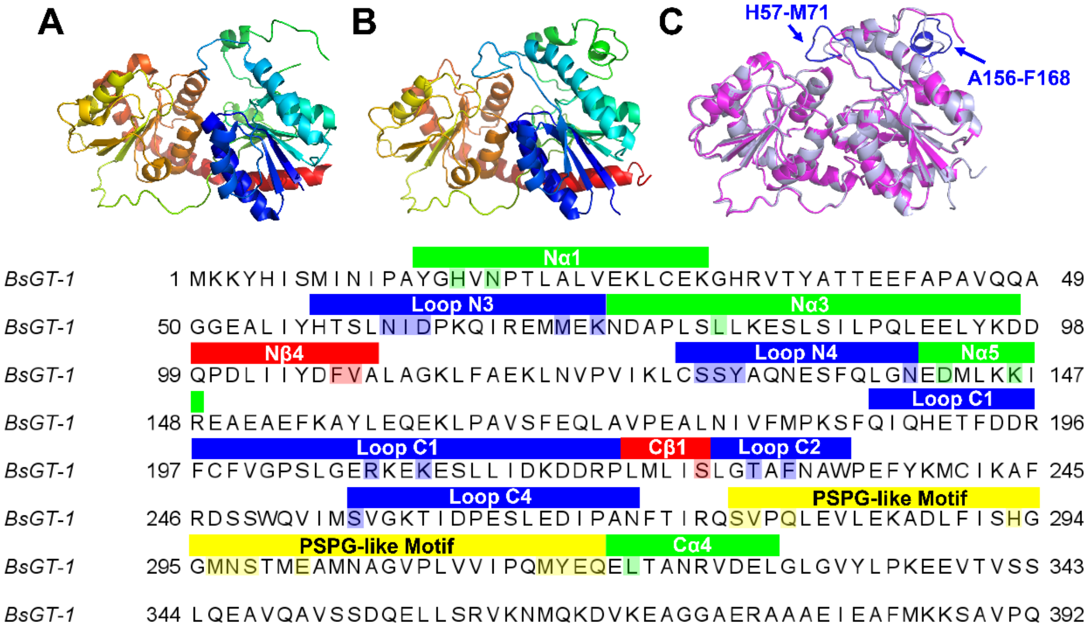

Supplementary Fig. 4 The incomplete crystal structure of BsGT-1 (7BOV) and its complete remedied structure. (A). The incomplete crystal structure of BsGT-1 with resolution at 2.29 Å. (B). The structure of BsGT-1 was remedied using the I-TASSER method (C). An optimal superposition of the 2.29 Å resolution of structure (magenta) and the remedial structure of BsGT-1 (lightblue) showed a high degree of structural identity with remedial secondary elements (H57-M71 and A156-F168) colored blue. (D). The protein sequence of BsGT-1 with 35 residues that formed hydrogen bonds or van der Waal's force interactions with ligands in typical regions were highlighted.

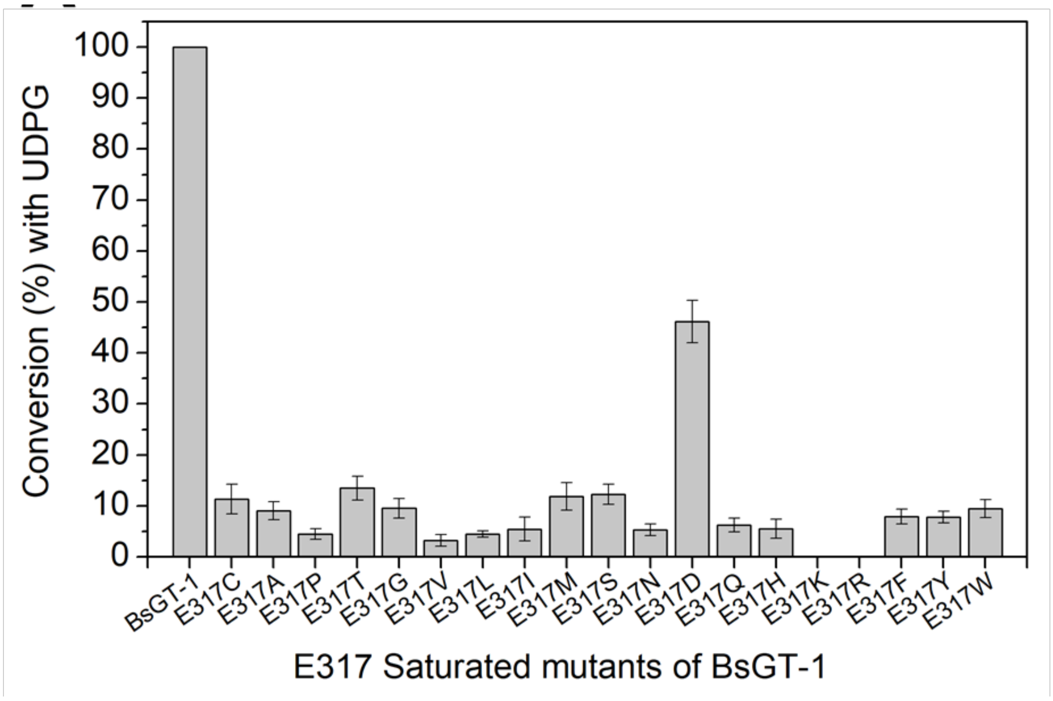

Supplementary Fig. 5 The conversions (mean ± SD) of glutamate (E317) saturated mutants toward UDPG (**2a**) after 10 h of incubation.

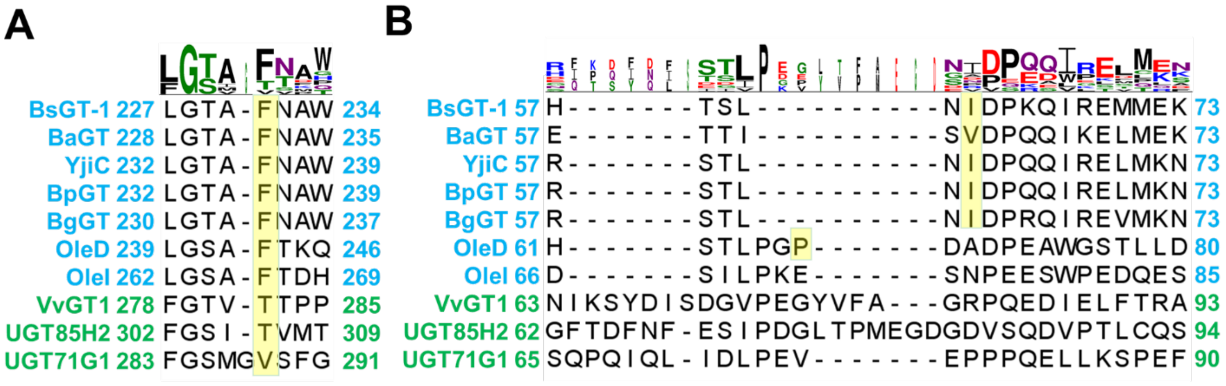

Supplementary Fig. 6 Functional mutations in the loop C2 (A) and N3 (B) regions of GTs. (A). Sequence alignment of the loop C2 region of the macrolide GTs and typical GTs with F231 residue labelled (yellow). (B). Sequence alignment of the loop N3 region of the grouping macrolide GTs (BsGT-1, BaGT, YjiC, BpGT and BgGT) with typical GTs OleD, OleI, VvGT1, UGT85H2 and UGT71G1. The I62 residue of BsGT-1 together with the aligned macrolide GTs and P67 residue of OleD were labelled yellow.

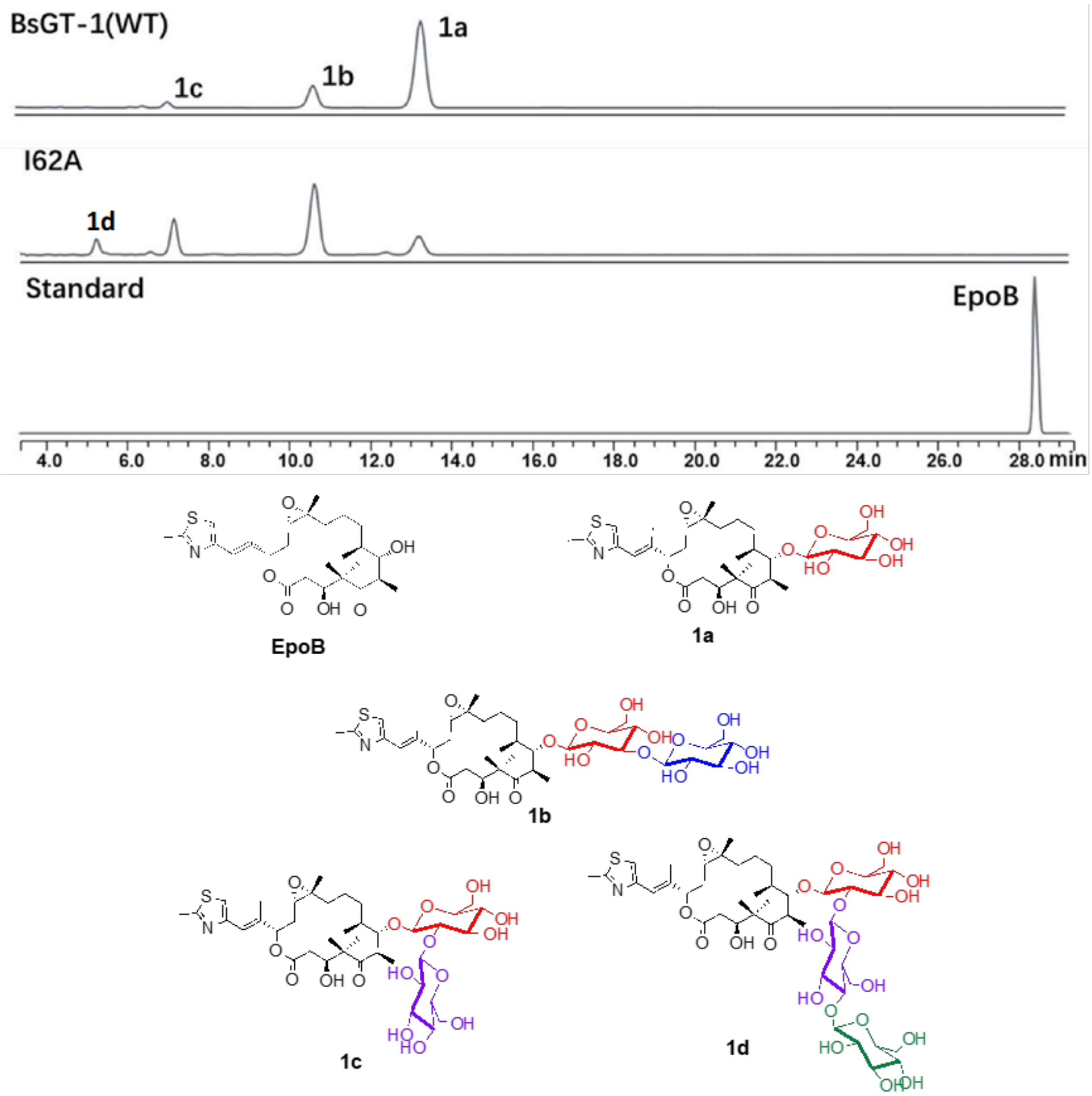

Supplementary Fig. 7 HPLC detection of the products from epothilone B glycosylation by wild-type BsGT-1 and I62A mutants. The control is the standard of epothilone B (EpoB). The reactions were performed for 10 h of incubation.

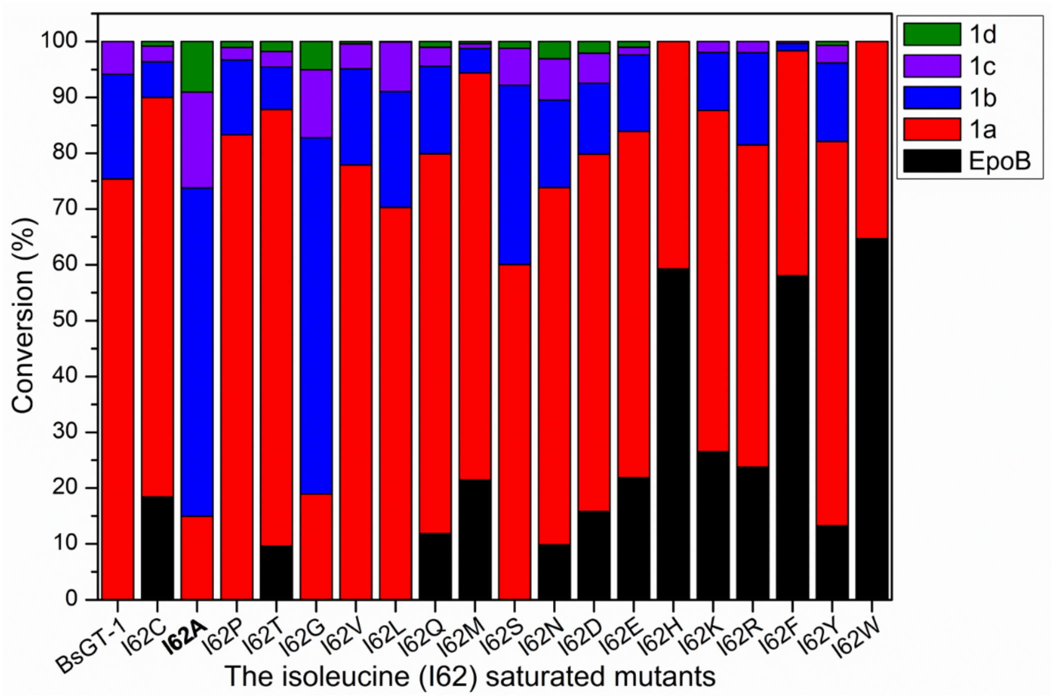

Supplementary Fig. 8 The conversion ratio (mean ± SD) of the glucosides products catalyzed by single residue isoleucine (I62) saturated mutants.

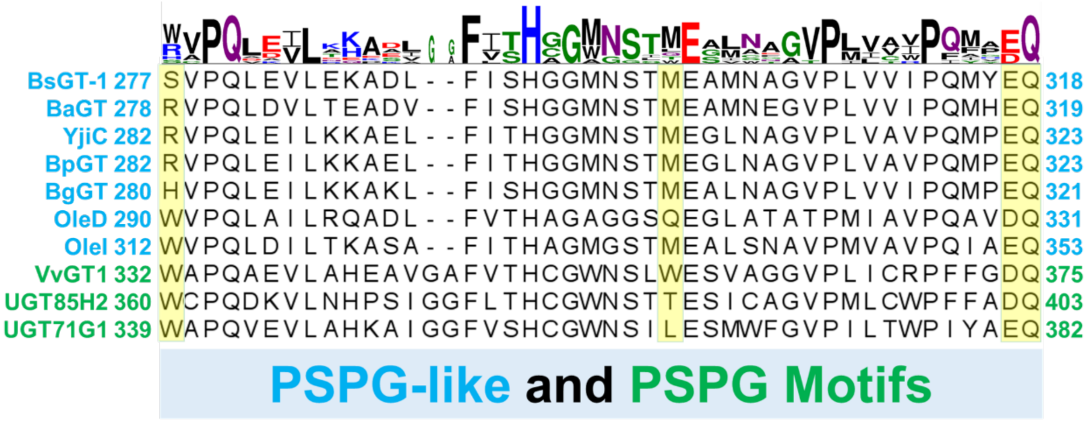

Supplementary Fig. 9 Sequence alignment of PSPG-like motifs of the macrolide GTs (BsGT-1, BaGT, YjiC, BpGT and BgGT) with the PSPG motifs of typical GTs, OleD (Entrez Protein accession code ABA42119, PDB ID 21YF), OleI (Entrez Protein accession code ABA42118, PDB ID 21YA), VvGT1 (Entrez Protein accession code AAB81683, PDB ID), UGT85H2 (Entrez Protein accession code ABE87250, PDB ID 2PQ6), UGT71G1 (Entrez Protein accession code AAW56092, PDB ID 2ACV). The key residues were labelled with yellow.

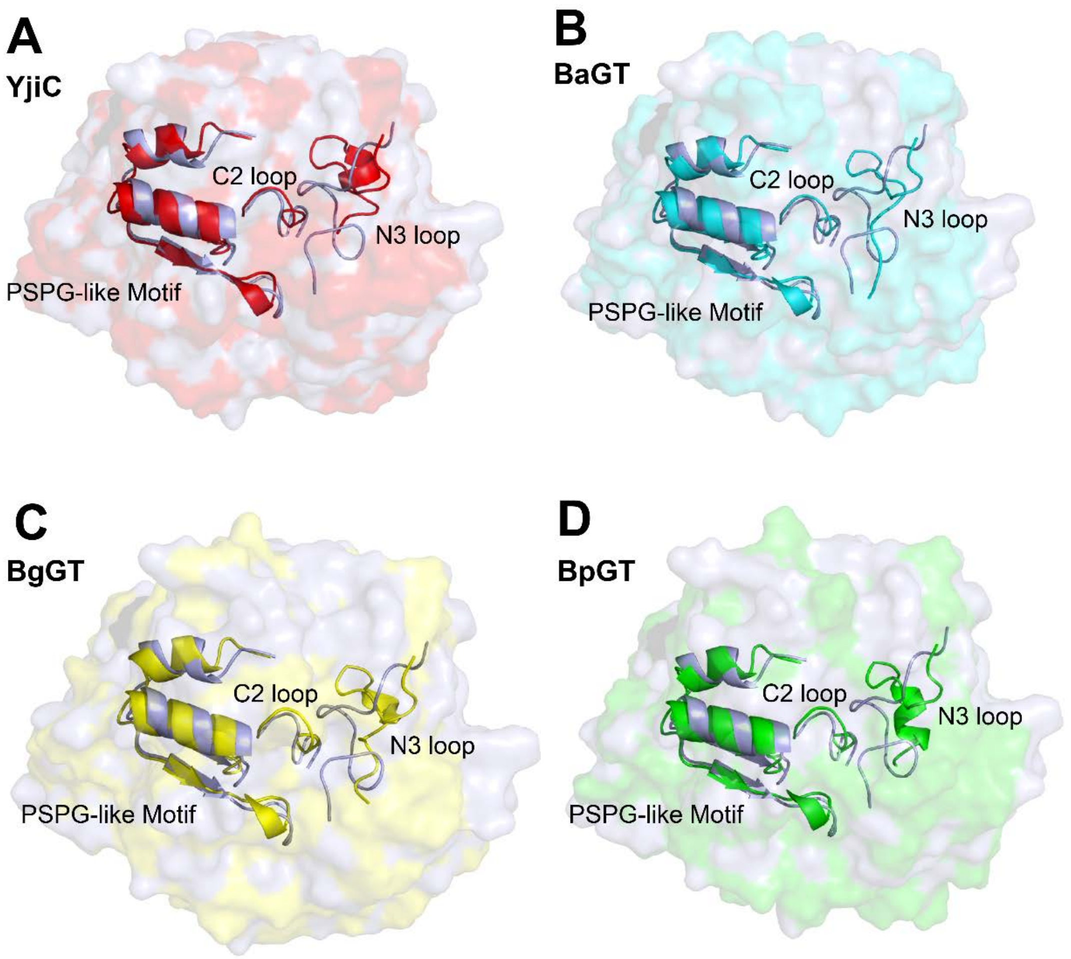

Supplementary Fig. 10 The optimal superposition of the four macrolide GT structures (YjiC, BaGT, BgGT and BpGT) with BsGT-1 were conducted using TM-align. The PSPG-like motif, C2 loop and N3 loop were labelled, respectively.

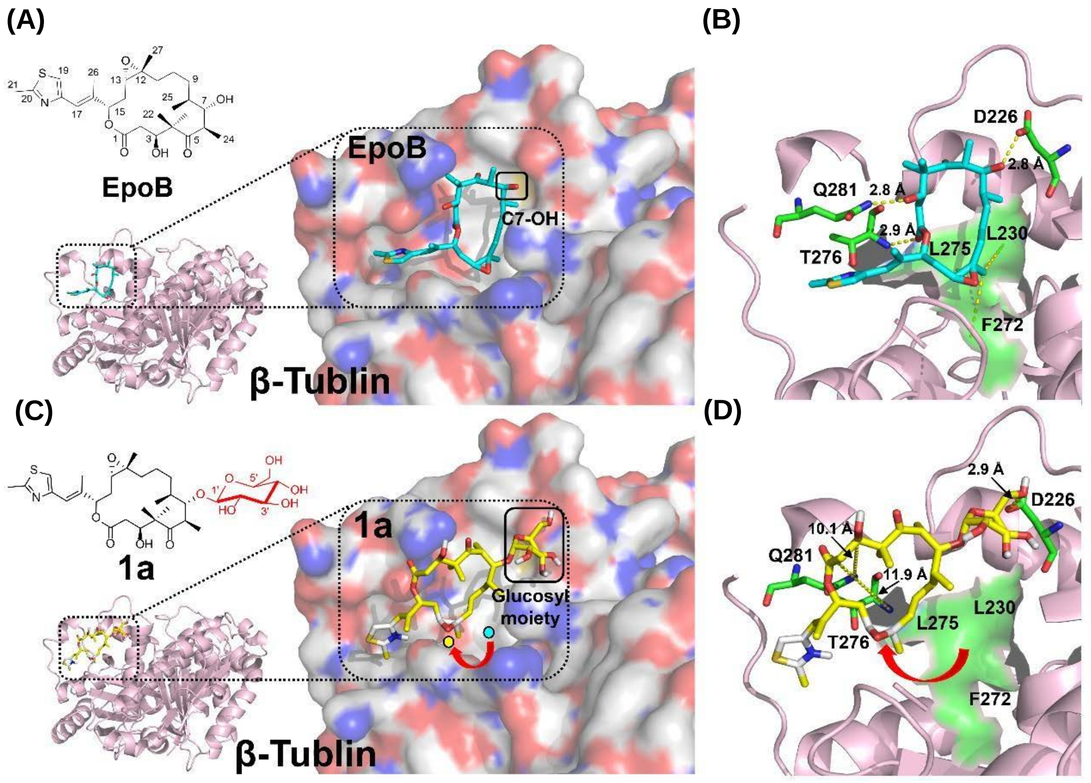

Supplementary Fig. 11 Molecular docking of EpoB and its mono-glycoside **1a** with β-tubulin subunit. The hydrogen bonding between ligand and the key residues of protein were exhibited. (A) The co-crystal structure (7DAE) showed that EpoB can be embedded in the hydrophobic cavity of the β-tubulin subunit; (B) The residues T276 and Q281 had hydrogen bonding interactions with C1-ketone group and C3-hydroxyl group of EpoB, respectively. The cavity formed by three residues L230, F272 and L275 possessed intimately hydrophobic interactions with triatomic oxygen ring (C12-O-C13). Notably, the key residue D226 of β-tubulin subunit formed a hydrogen bond with the C7-hydroxyl group of EpoB; (C) In **1a**, the sugar moiety occupied the C7-hydroxyl group of EpoB, which blocked the hydrogen bonds interaction and caused huge steric hindrances. (D) The residues T276 and Q281 lost the hydrogen bonds interactions with **1a** (> 10 Å), and the triatomic oxygen ring generated an offset with respect to the cavity formed by three residues L230, F272 and L275. D226 cannot make

connections with the occupied C7-hydroxyl group, but formed a new hydrogen bond with the C6-hydroxyl group of the sugar bulk, which may enhance the binding force between glycoside and tubulin.

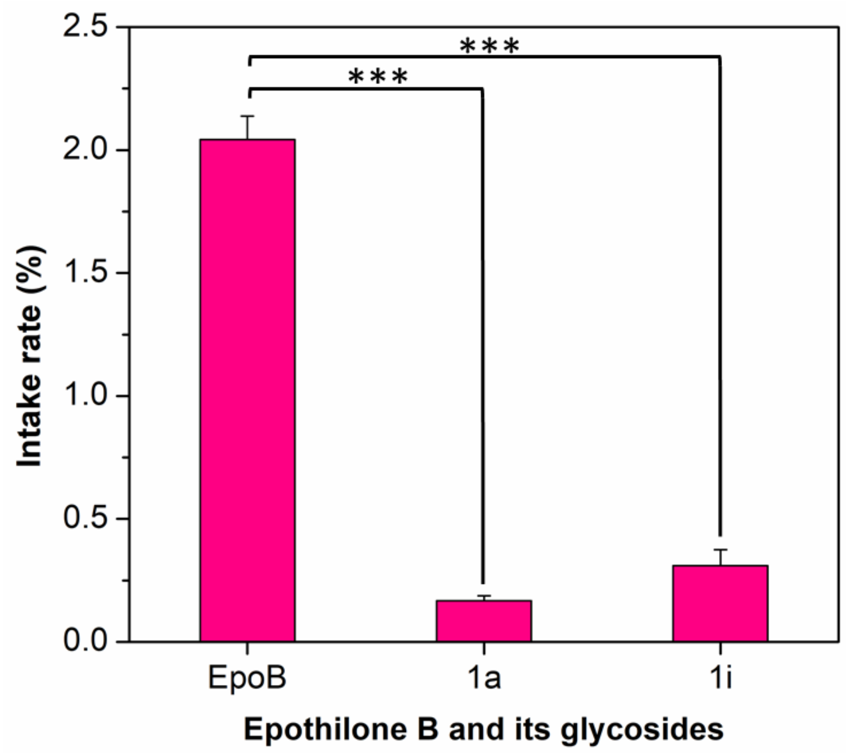

Supplementary Fig. 12 The comparison of cellular intake rate of glycosides **1a** and **1i** with EpoB (mean ± SD, the statistical significance level is **$p < 0.01$, ***$p < 0.001$).

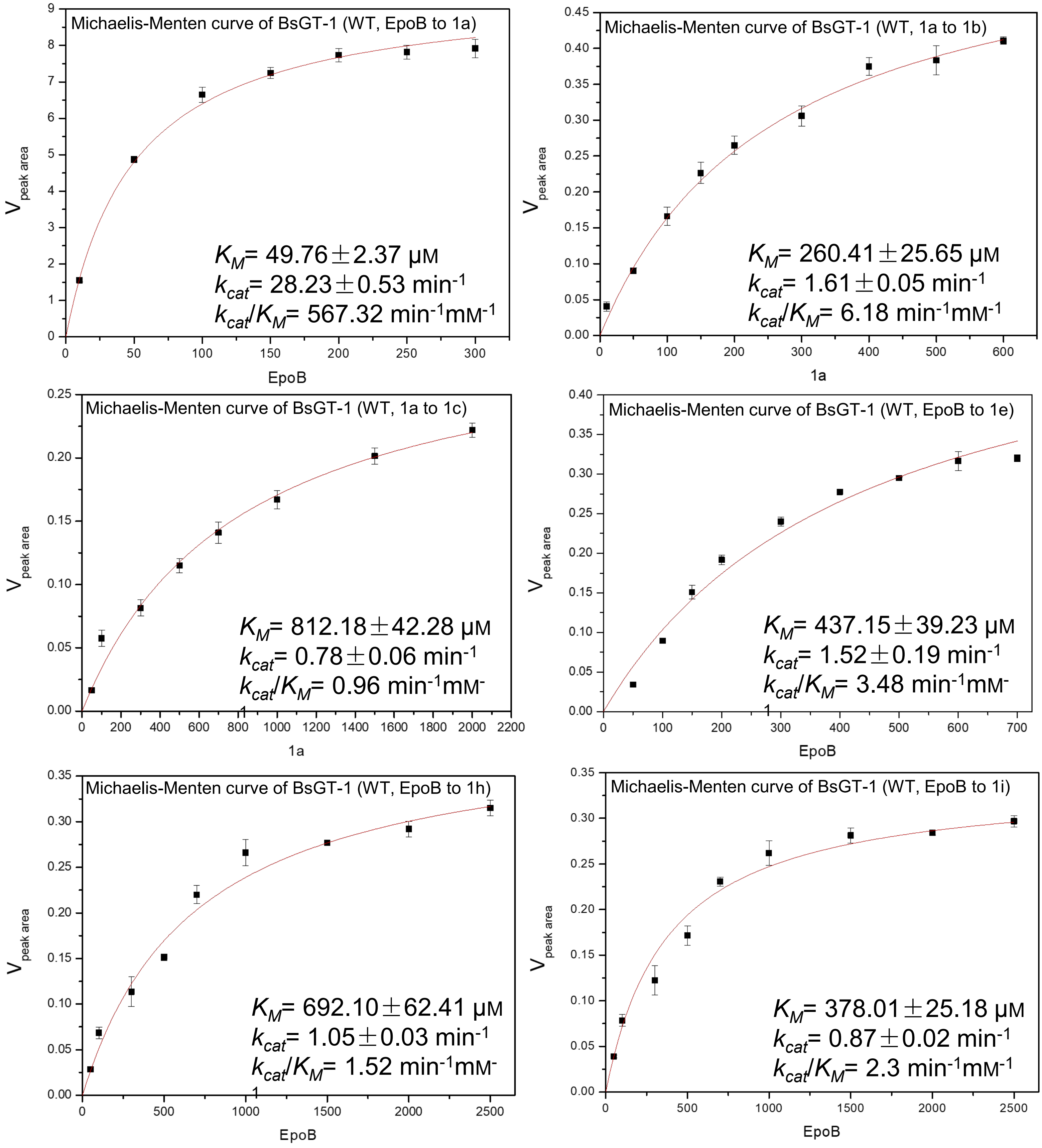

Supplementary Fig. 13 Kinetic parameters and fitting curves analysis of BsGT-1 (WT). Determination of kinetic parameters for **1a**, **1b**, **1c**, **1e**, **1h** and **1i** with saturated sugar donors (**2a**, **2e**, **2h** and **2i**). Enzyme assays were performed in 50 mM Tris-HCl buffer (pH 7.5) containing 20 μg/ml protein and 10 mM $MgCl_2$ at 37°C for 10 min in triplicate.

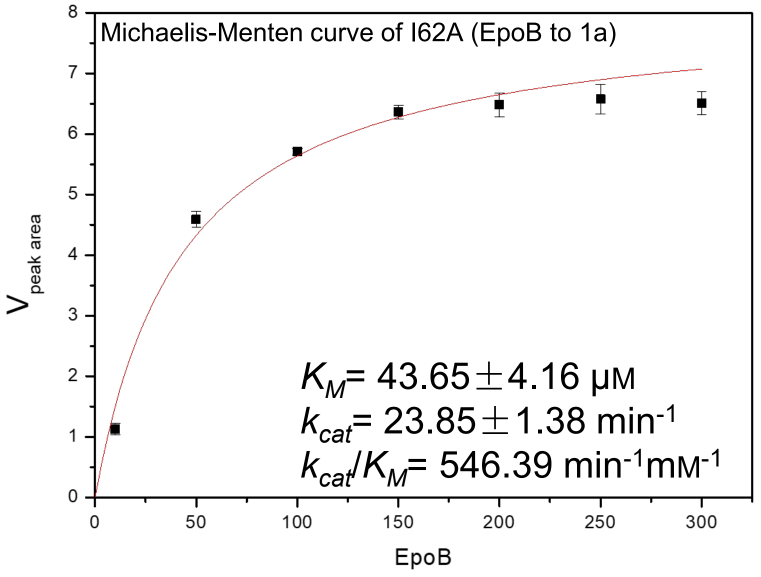

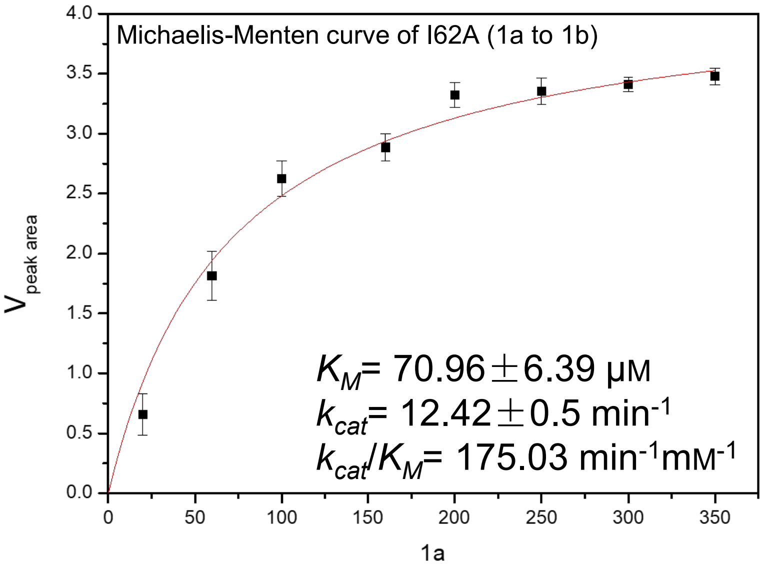

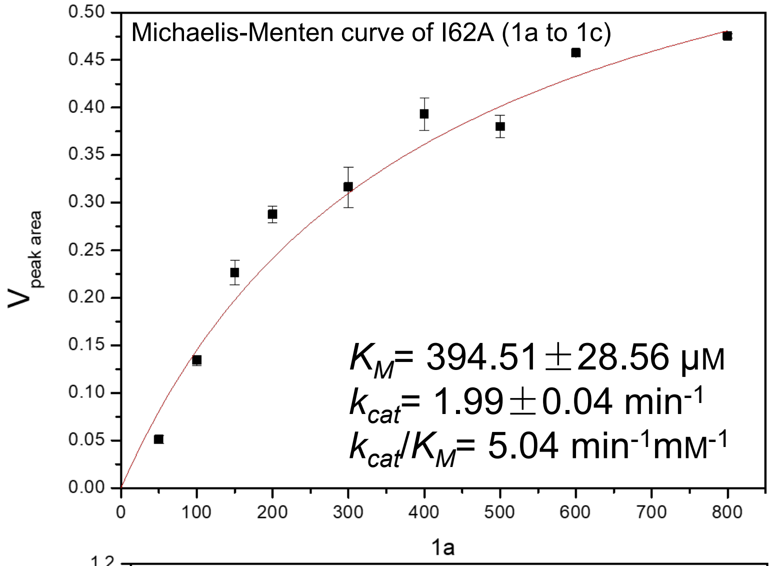

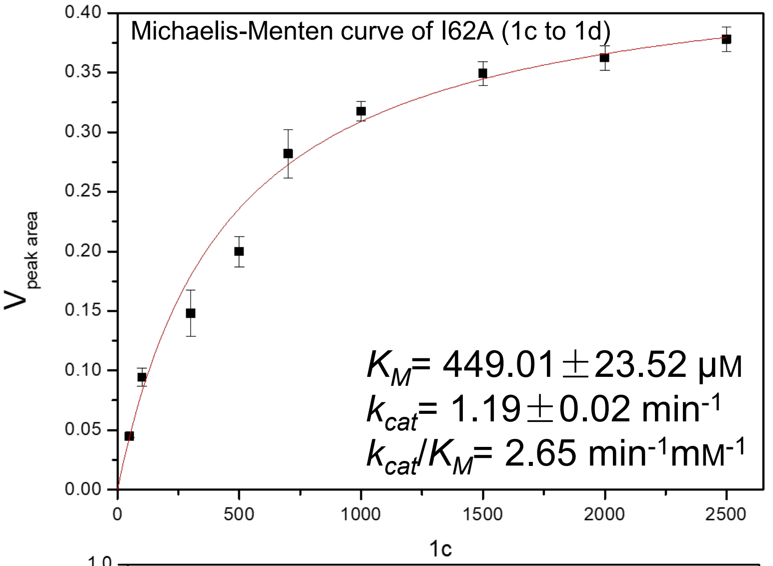

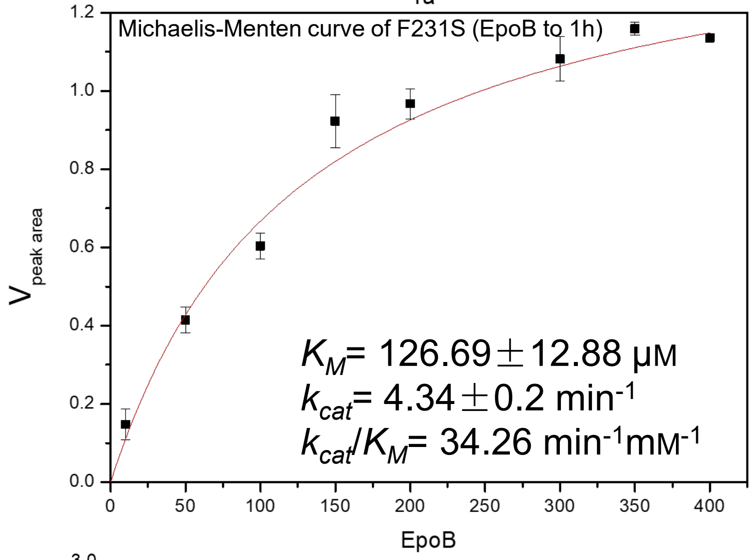

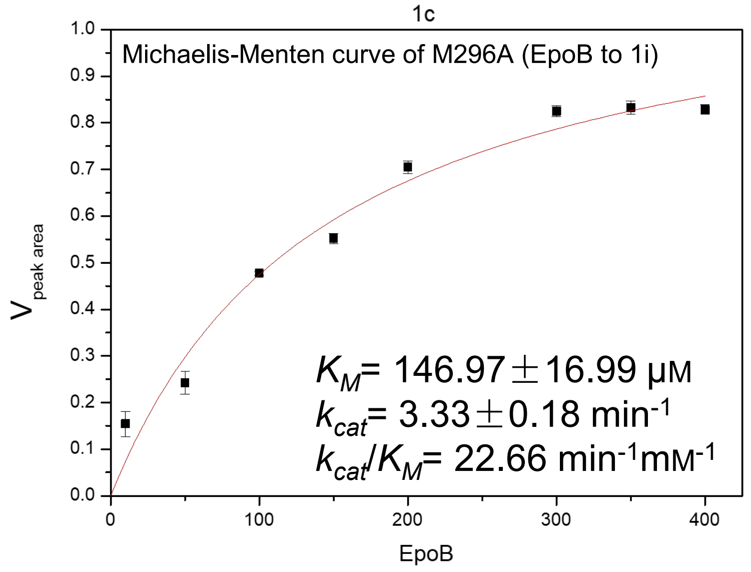

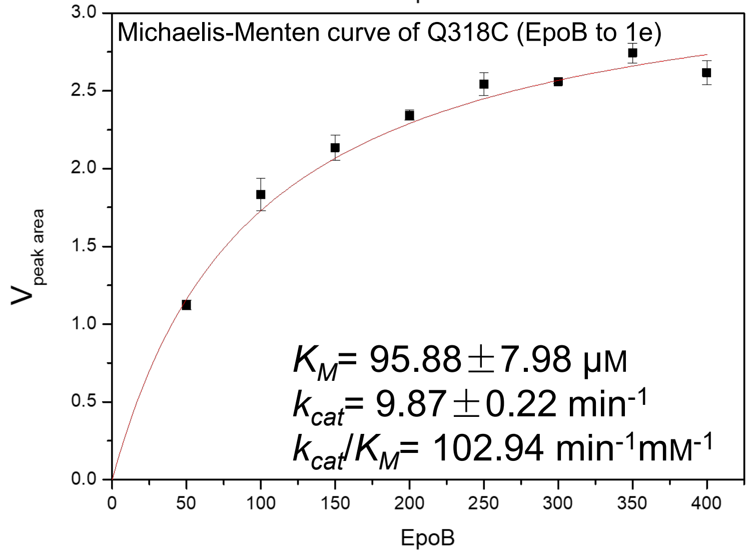

Supplementary Fig. 14 Kinetic parameters and fitting curves analysis of the BsGT-1 mutants. Determination of kinetic parameters for **1a**, **1b**, **1c**, **1e**, **1h** and **1i** with saturated sugar donors (**2a**, **2e**, **2h** and **2i**).

Supplementary Note

Spectra list of new compounds: Supplementary Figures 15-62

Supplementary Fig. 15 High-resolution mass spectrum of **1a**.

Supplementary Fig. 16 High-resolution mass spectrum of **1b**.

Supplementary Fig. 17 High-resolution mass spectrum of **1c**.

Supplementary Fig. 18 High-resolution mass spectrum of **1d**.

Supplementary Fig. 19 High-resolution mass spectrum of **1h**.

Supplementary Fig. 20 High-resolution mass spectrum of **1i**.

Supplementary Fig. 21 High-resolution mass spectrum of **1e**.

Supplementary Fig. 22 High-resolution mass spectrum of **1f**.

Supplementary Fig. 23 High-resolution mass spectrum of **1l**.

Supplementary Fig. 24 High-resolution mass spectrum of **1m**.

Supplementary Fig. 25 High-resolution mass spectrum of **1o**.

Supplementary Fig. 26 High-resolution mass spectrum of **1p**.

Supplementary Fig. 27 ^{1}H NMR spectrum (600 MHz) of **1a** in CD3CD.

Supplementary Fig. 28 ^{13}C NMR spectrum (600 MHz) of **1a** in CD3CD.

Supplementary Fig. 29 HSQC spectrum (600 MHz) of **1a** in CD3CD.

Supplementary Fig. 30 HMBC spectrum (600 MHz) of **1a** in CD3CD.

Supplementary Fig. 31 ^{1}H-^{1}H COSY spectrum (600 MHz) of **1a** in CD3CD.

Supplementary Fig. 32 ^{1}H NMR spectrum (600 MHz) of **1b** in CD3CD.

Supplementary Fig. 33 ^{13}C NMR spectrum (600 MHz) of **1b** in CD3CD.

Supplementary Fig. 34 HSQC spectrum (600 MHz) of **1b** in CD3CD.

Supplementary Fig. 35 HMBC spectrum (600 MHz) of **1b** in CD3CD.

Supplementary Fig. 36 ^{1}H-^{1}H COSY spectrum (600 MHz) of **1b** in CD3CD.

Supplementary Fig. 37 ^{1}H NMR spectrum (600 MHz) of **1c** in CD3CD.

Supplementary Fig. 38 ^{13}C NMR spectrum (600 MHz) of **1c** in CD3CD.

Supplementary Fig. 39 HSQC spectrum (600 MHz) of **1c** in CD3CD.

Supplementary Fig. 40 HMBC spectrum (600 MHz) of **1c** in CD3CD.

Supplementary Fig. 41 ^{1}H-^{1}H COSY spectrum (600 MHz) of **1c** in CD3CD.

Supplementary Fig. 42 ^{1}H NMR spectrum (600 MHz) of **1d** in CD3CD.

Supplementary Fig. 43 ^{13}C NMR spectrum (600 MHz) of **1d** in CD3CD.

Supplementary Fig. 44 HSQC spectrum (600 MHz) of **1d** in CD3CD.

Supplementary Fig. 45 HMBC spectrum (600 MHz) of **1d** in CD3CD.

Supplementary Fig. 46 ^{1}H-^{1}H COSY spectrum (600 MHz) of **1d** in CD3CD.

Supplementary Fig. 47 ^{1}H-^{1}H TOCSY spectrum (600 MHz) of **1d** in CD3CD.

Supplementary Fig. 48 ^{1}H NMR spectrum (600 MHz) of **1h** in CD3CD.

Supplementary Fig. 49 ^{13}C NMR spectrum (600 MHz) of **1h** in CD3CD.

Supplementary Fig. 50 HSQC spectrum (600 MHz) of **1h** in CD3CD.

Supplementary Fig. 51 HMBC spectrum (600 MHz) of **1h** in CD3CD.

Supplementary Fig. 52 ^{1}H-^{1}H COSY spectrum (600 MHz) of **1h** in CD3CD.

Supplementary Fig. 53 ^{1}H NMR spectrum (600 MHz) of **1i** in CD3CD.

Supplementary Fig. 54 ^{13}C NMR spectrum (600 MHz) of **1i** in CD3CD.

Supplementary Fig. 55 HSQC spectrum (600 MHz) of **1i** in CD3CD.

Supplementary Fig. 56 HMBC spectrum (600 MHz) of **1i** in CD3CD.

Supplementary Fig. 57 ^{1}H-^{1}H COSY spectrum (600 MHz) of **1i** in CD3CD.

Supplementary Fig. 58 ^{1}H NMR spectrum (600 MHz) of **1e** in CD3CD.

Supplementary Fig. 59 ^{13}C NMR spectrum (600 MHz) of **1e** in CD3CD.

Supplementary Fig. 60 HSQC spectrum (600 MHz) of **1e** in CD3CD.

Supplementary Fig. 61 HMBC spectrum (600 MHz) of **1e** in CD3CD.

Supplementary Fig. 62 ^{1}H-^{1}H COSY spectrum (600 MHz) of **1e** in CD3CD.

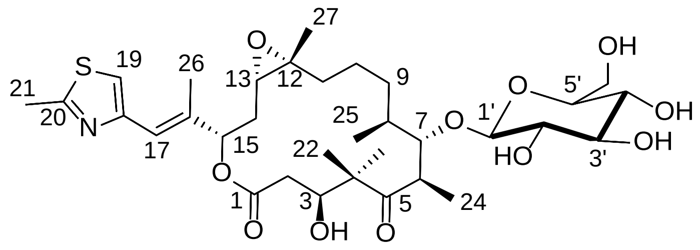

1a: Epothilone B 7-*O*-β-D glucoside

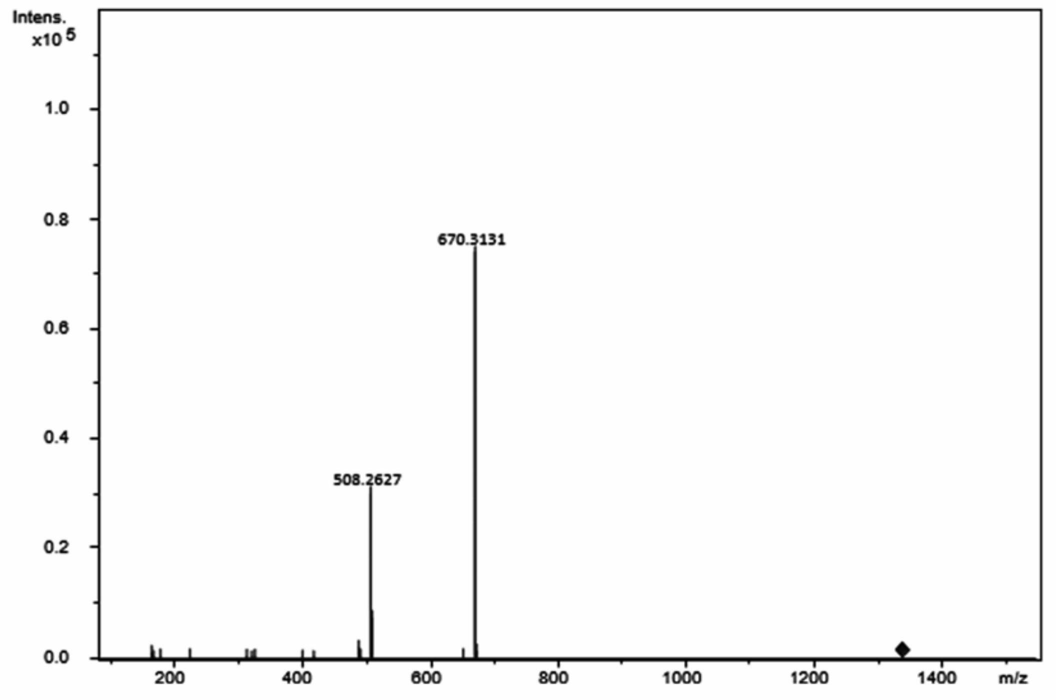

Supplementary Fig. 15 ESI-MS identification of product **1a** using epothilone B as an acceptor and UDPG as a sugar donor.

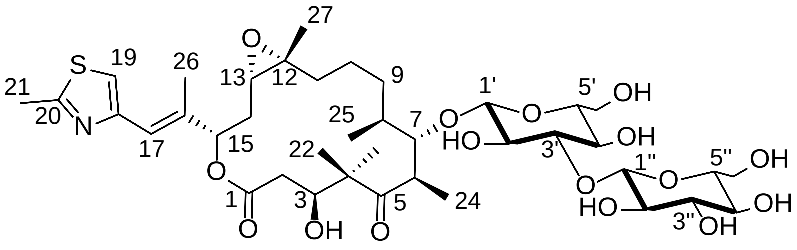

1b: Epothilone B 7-*O*-β-D-glucosyl-(1→3)-β-D glucoside

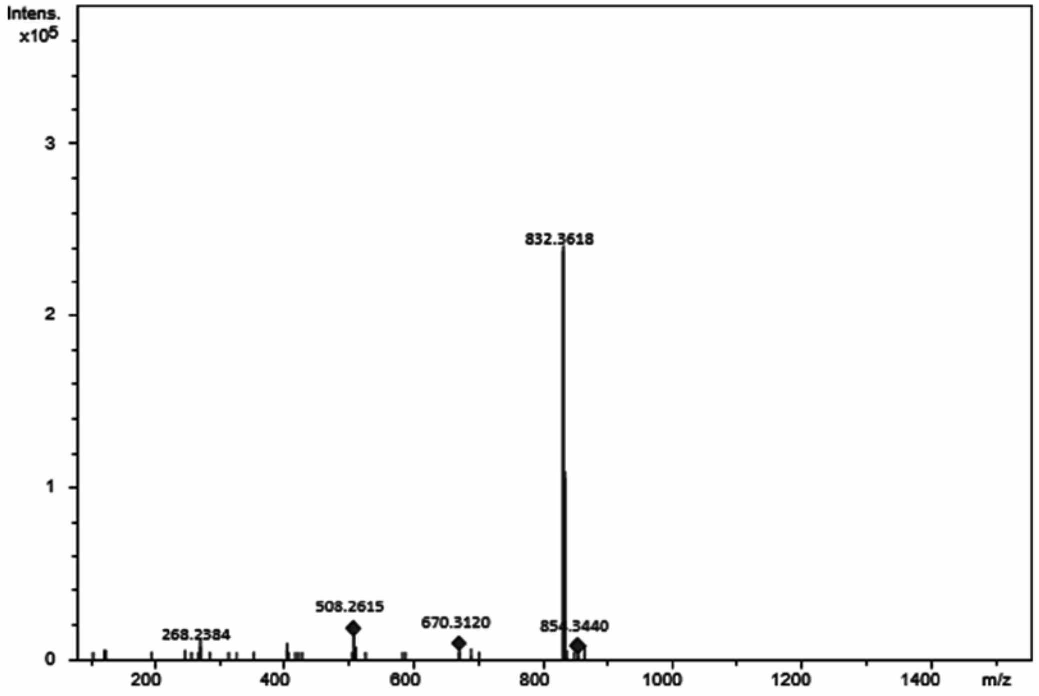

Supplementary Fig. 16 ESI-MS identification of product **1b** using epothilone B as an acceptor and UDPG as a sugar donor.

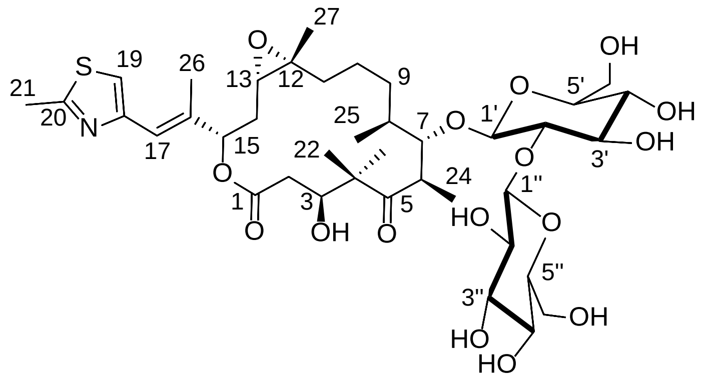

1c: Epothilone B 7-*O*-β-D-glucosyl-(1→2)-β-D glucoside

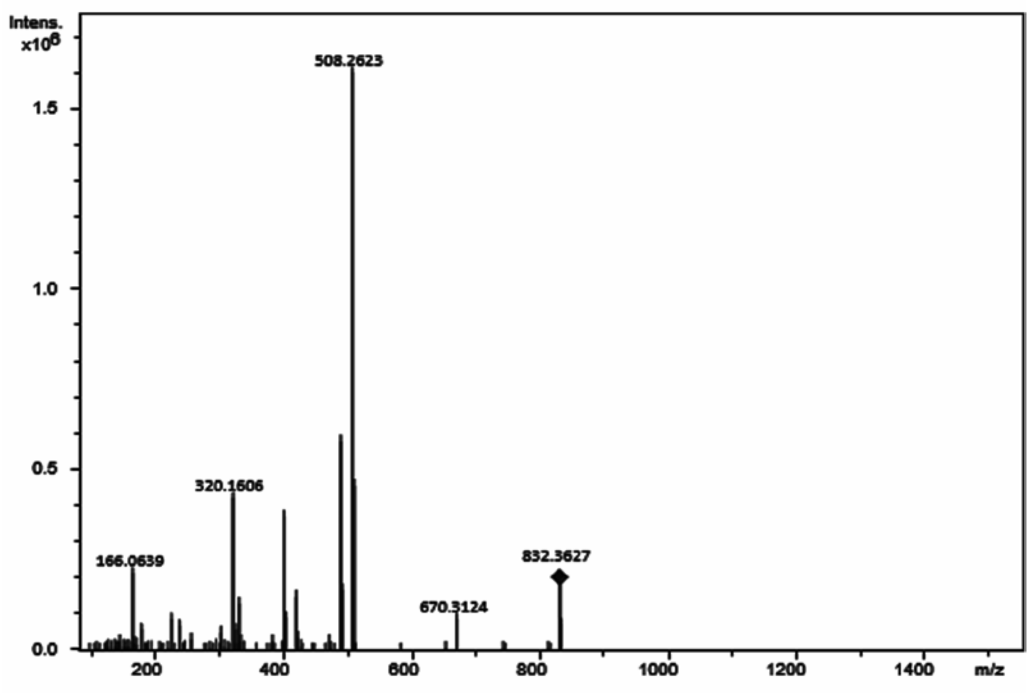

Supplementary Fig. 17 ESI-MS identification of product **1c** using epothilone B as an acceptor and UDPG as a sugar donor.

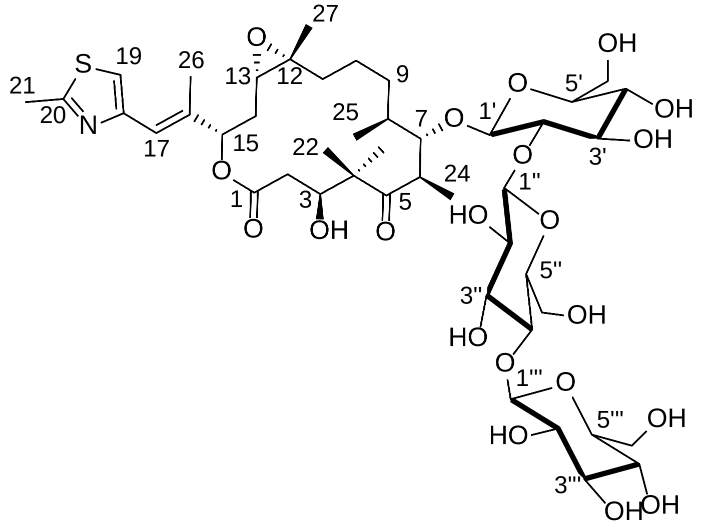

1d: Epothilone B 7-*O*-β-D-glucosyl-(1→2)-β-D-glucosyl-(1→4)-β-D glucoside

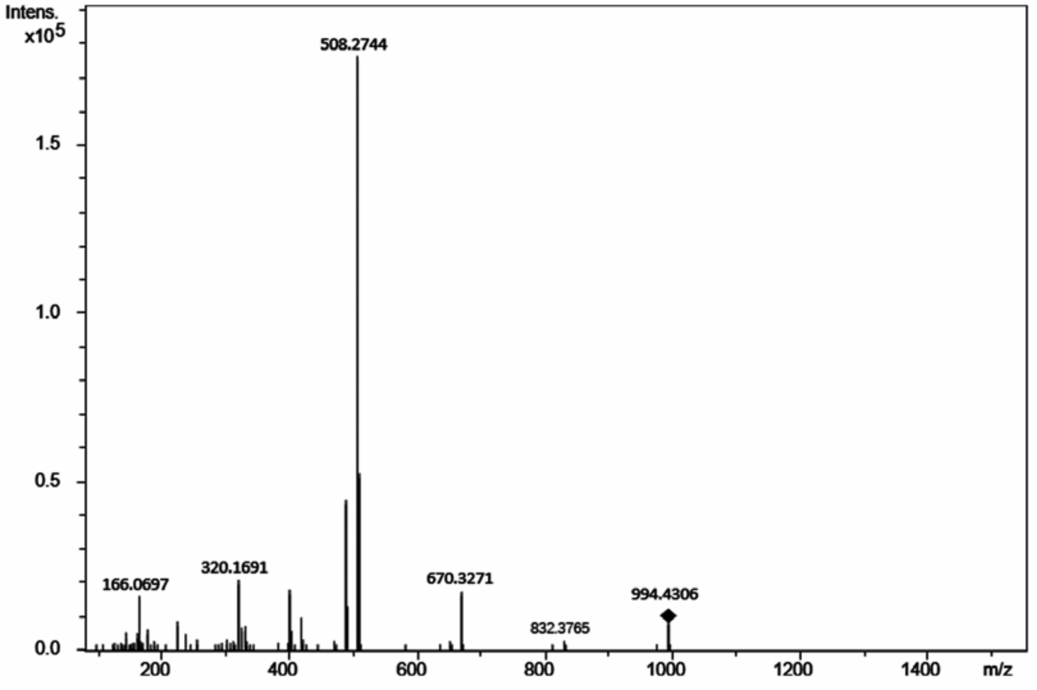

Supplementary Fig. 18 ESI-MS identification of product **1d** using epothilone B as an acceptor and UDPG as a sugar donor.

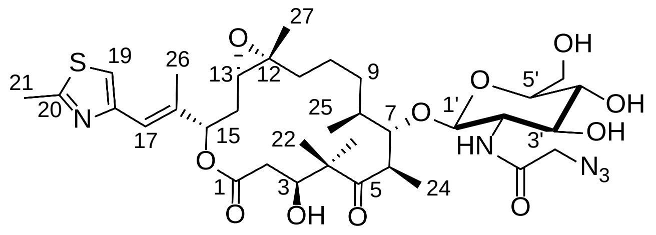

1h: Epothilone B 7-*O*-β-D GlcNAz

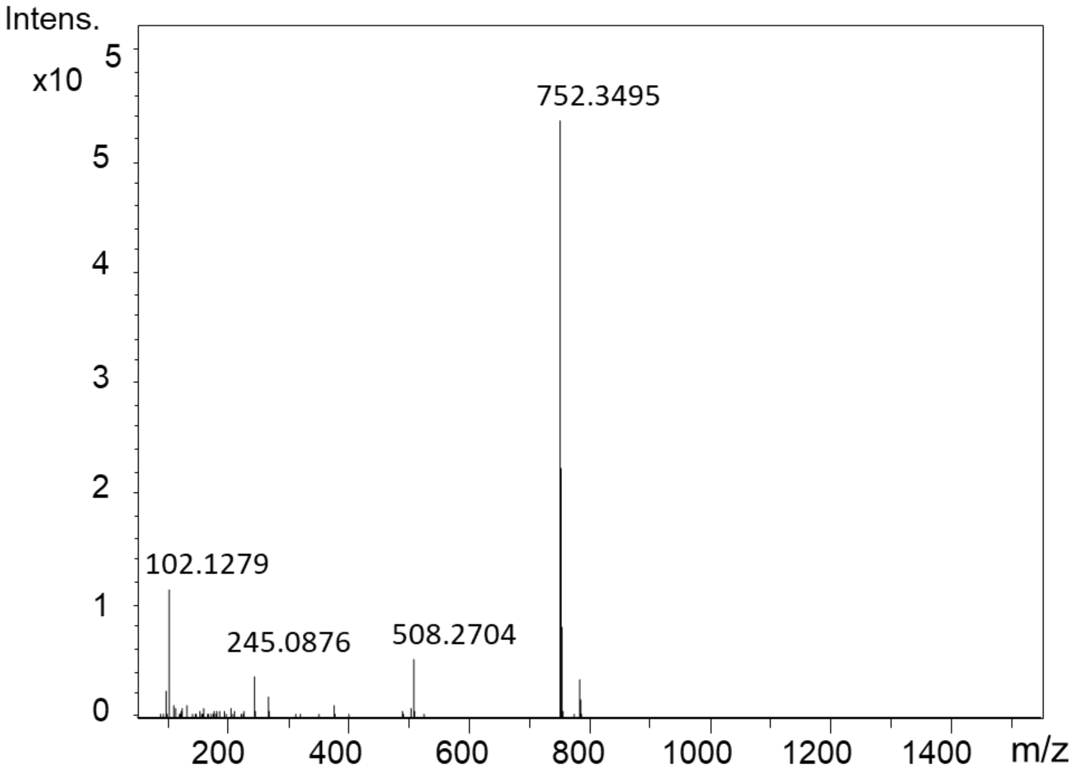

Supplementary Fig. 19 ESI-MS identification of product **1h** using epothilone B as an acceptor and UDP-GlcNAz as a sugar donor.

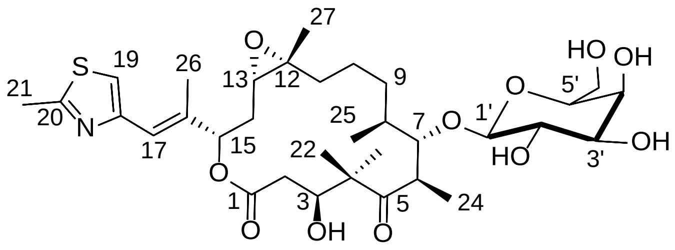

1i: Epothilone B 7-*O*-β-D galactoside

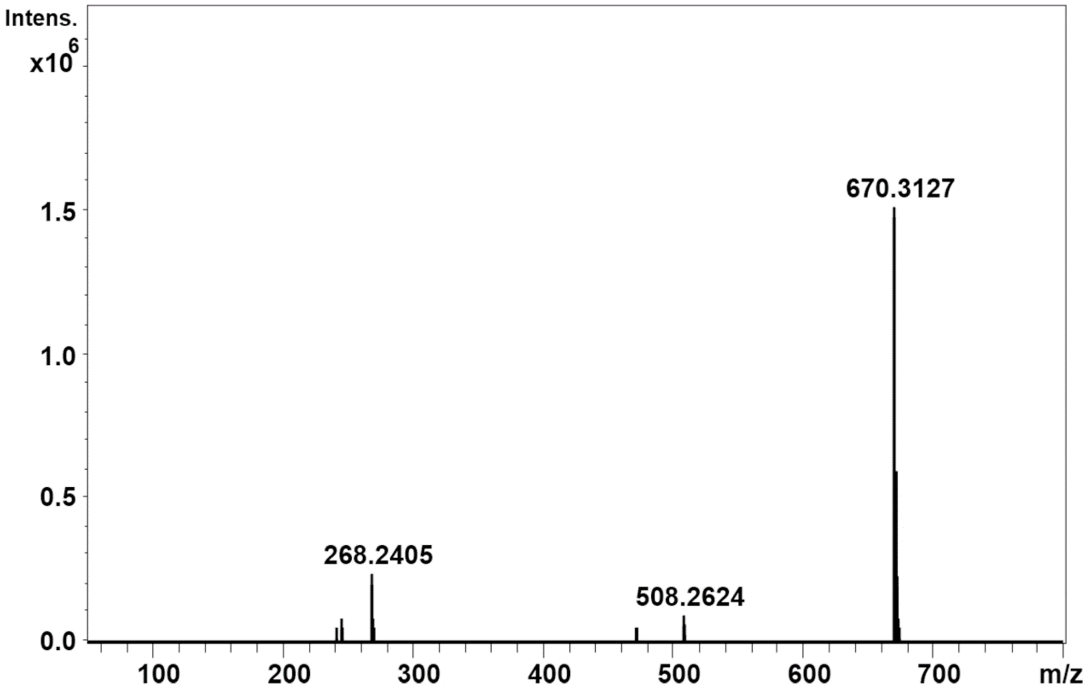

Supplementary Fig. 20 ESI-MS identification of product **1i** using epothilone B as an acceptor and UDP-Galactose as a sugar donor.

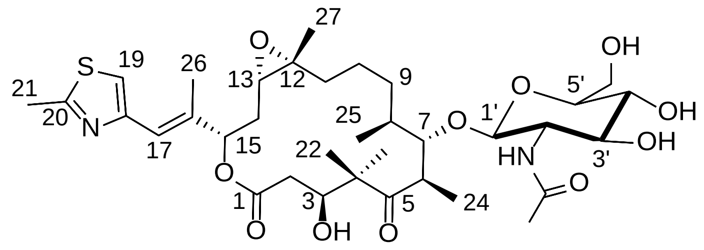

1e: Epothilone B 7-*O*-β-D GlcNAc

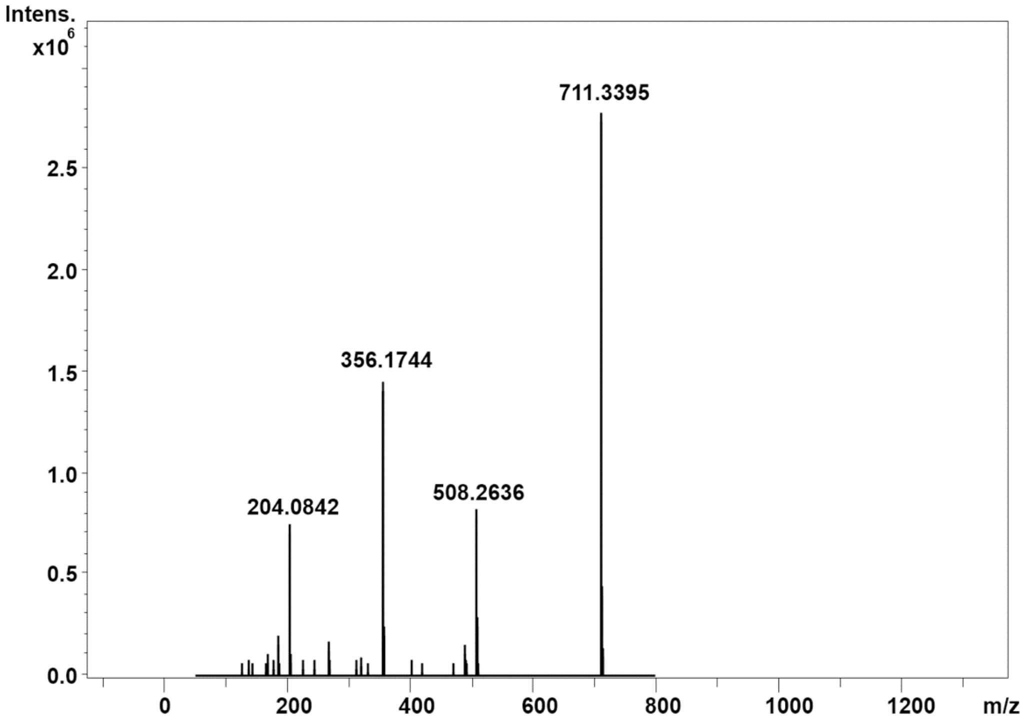

Supplementary Fig. 21 ESI-MS identification of product **1e** using epothilone B as an acceptor and UDP-GlcNAc as a sugar donor.

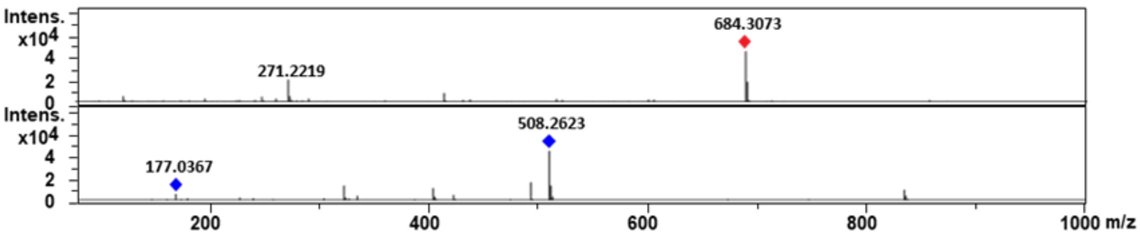

Supplementary Fig. 22 ESI-MS/MS identification of product **1f** using epothilone B as an acceptor and UDP-GlcA as a sugar donor.

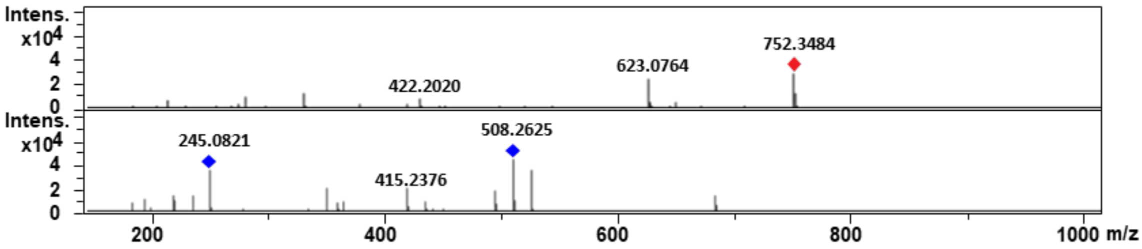

Supplementary Fig. 23 ESI-MS/MS identification of product **1l** using epothilone B as an acceptor and UDP-GalNAz as a sugar donor.

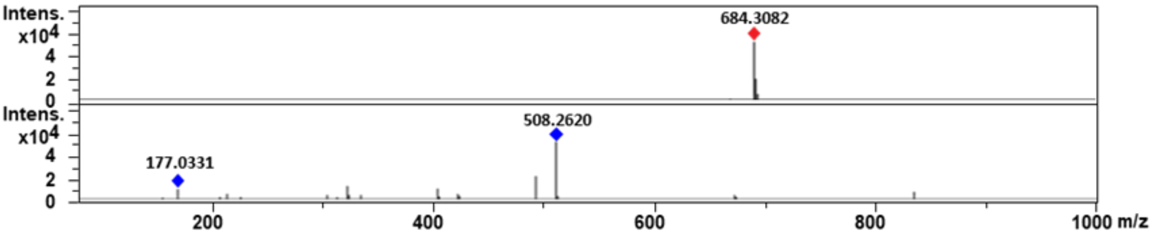

Supplementary Fig. 24 ESI-MS/MS identification of product **1m** using epothilone B as an acceptor and UDP-GalA as a sugar donor.

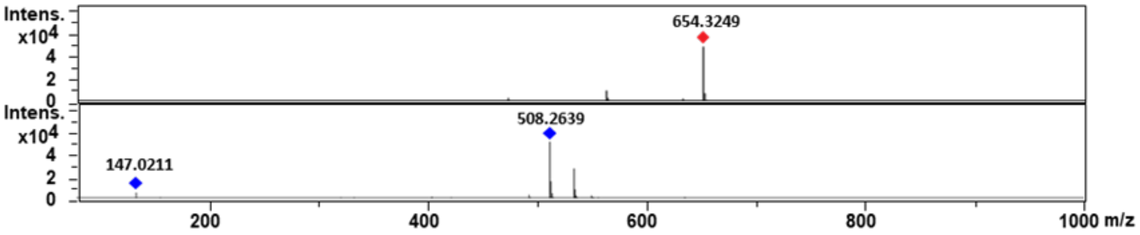

Supplementary Fig. 25 ESI-MS/MS identification of product **1o** using epothilone B as an acceptor and GDP-Fuc as a sugar donor.

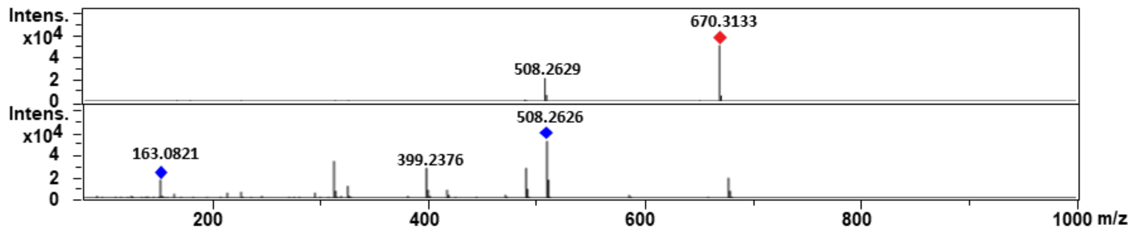

Supplementary Fig. 26 ESI-MS/MS identification of product **1p** using epothilone B as an acceptor and GDP-Man as a sugar donor.

Supplementary Fig. 27

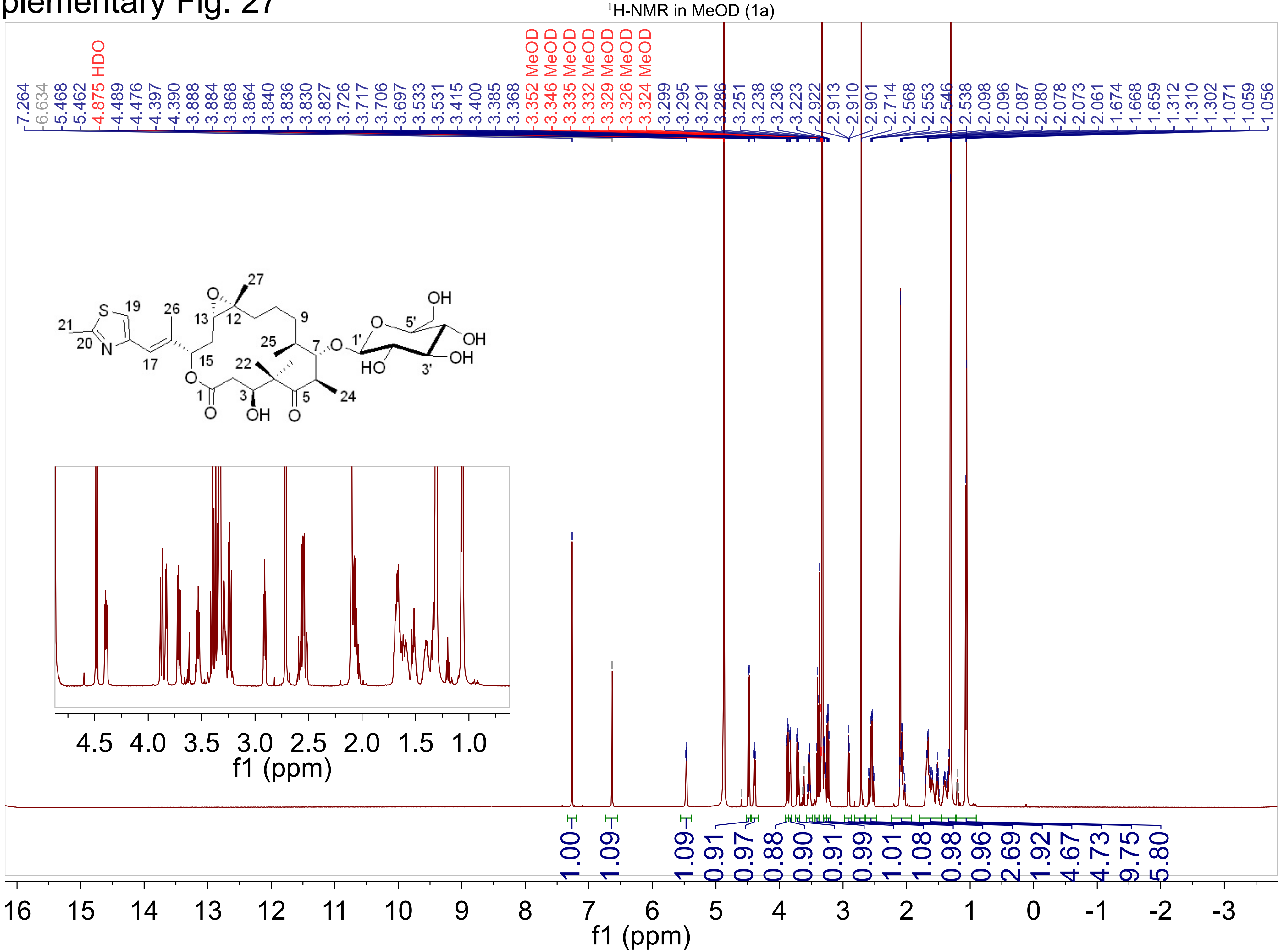

Supplementary Fig. 28

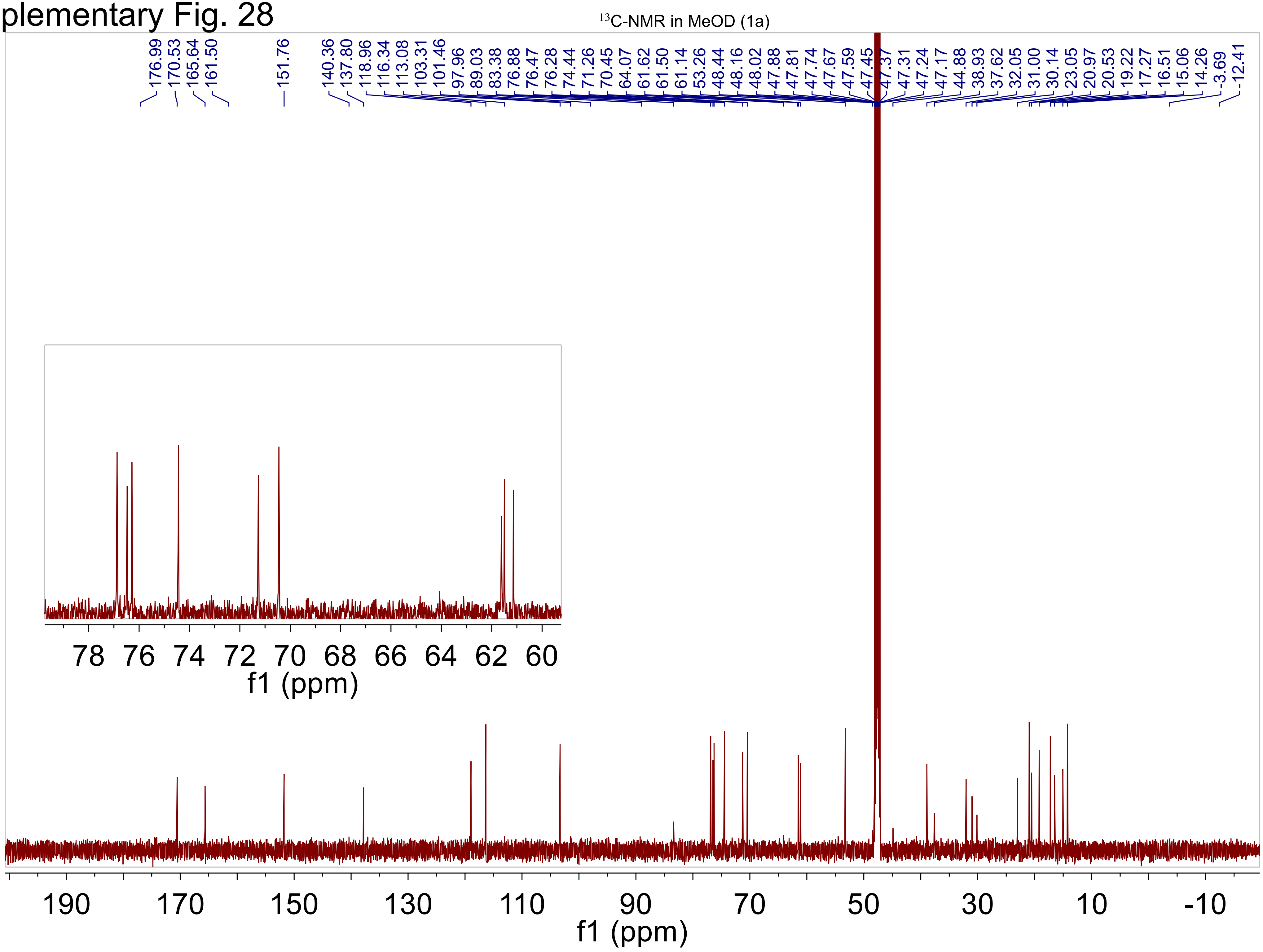

Supplementary Fig. 29

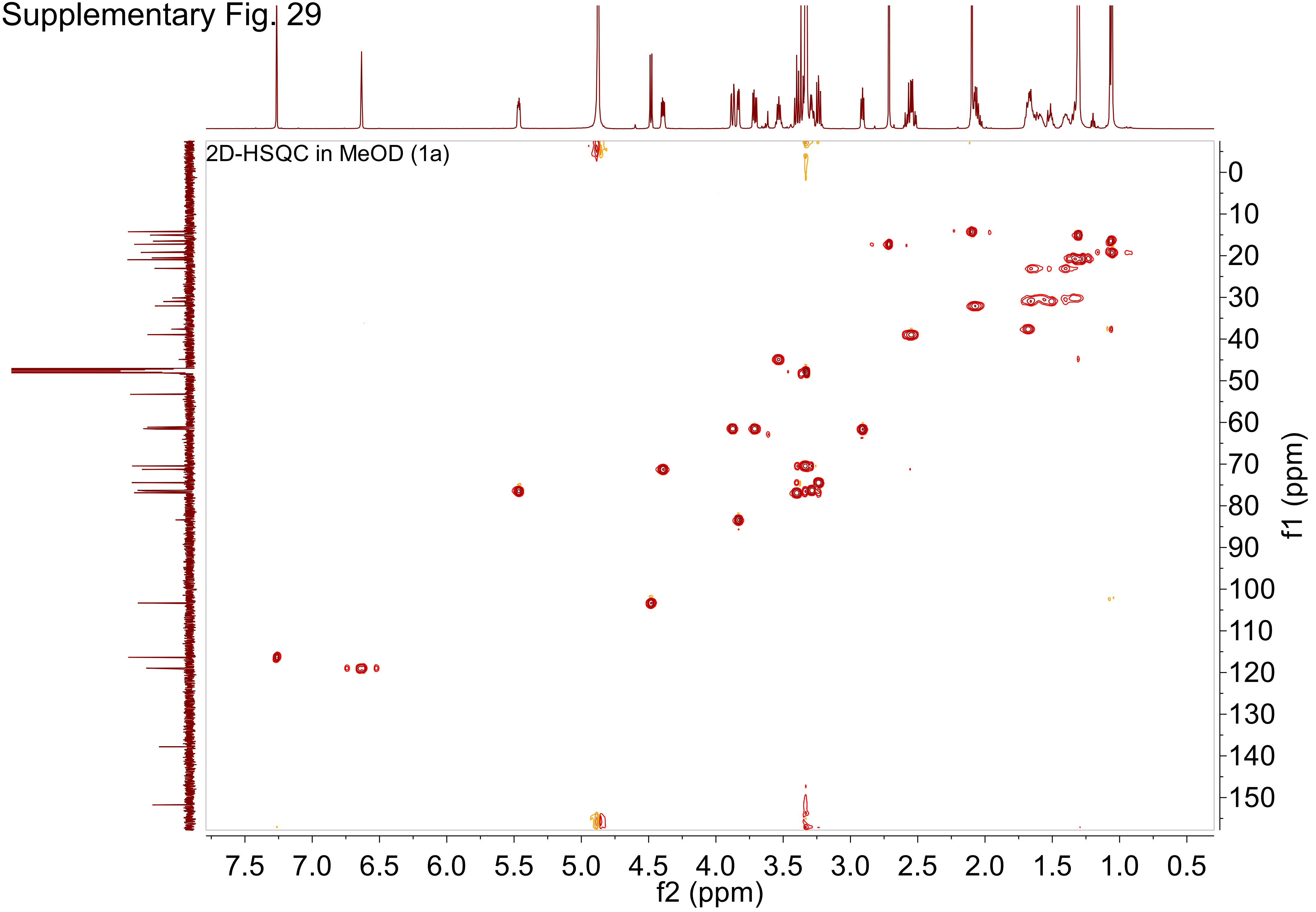

Supplementary Fig. 30

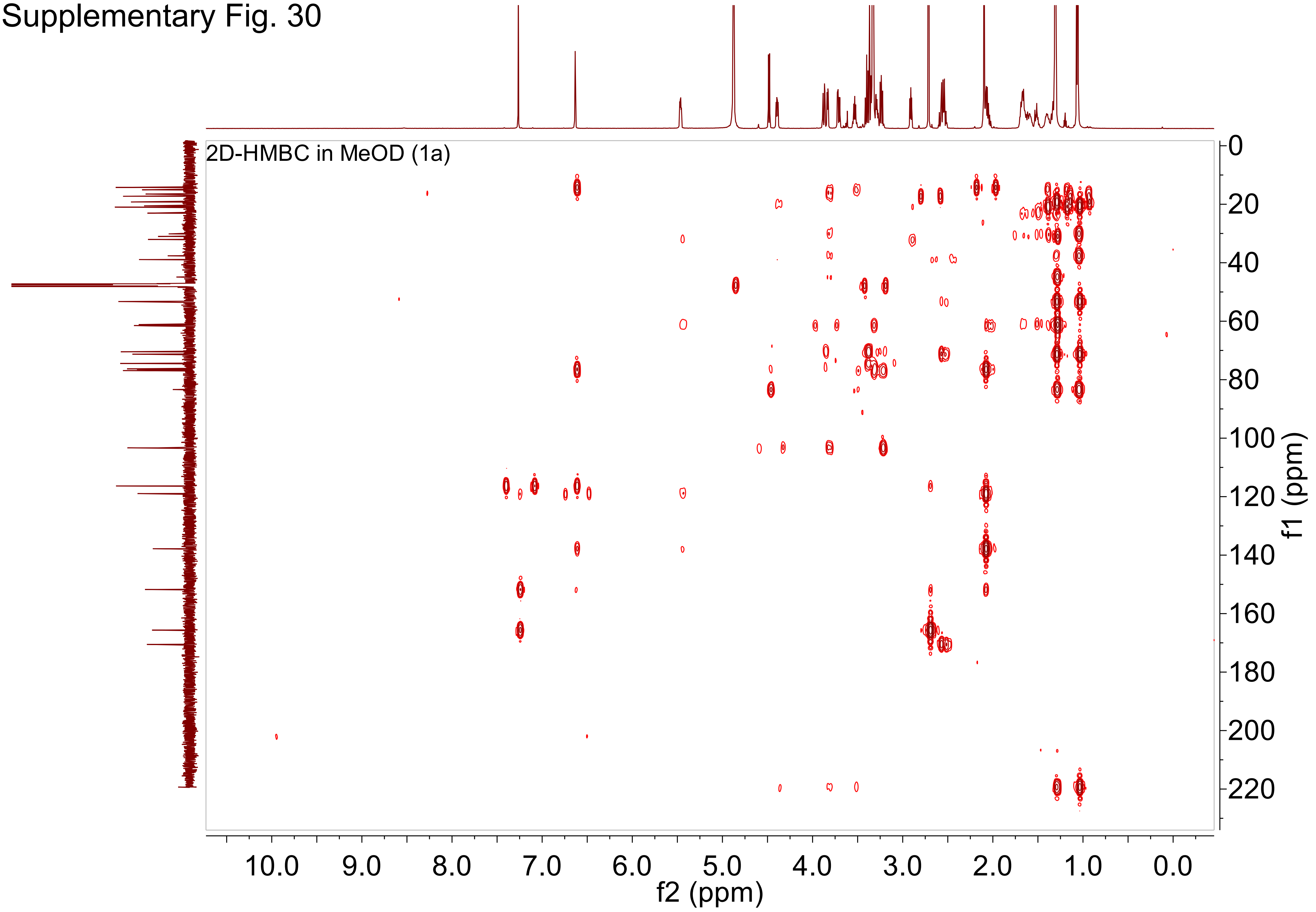

Supplementary Fig. 31

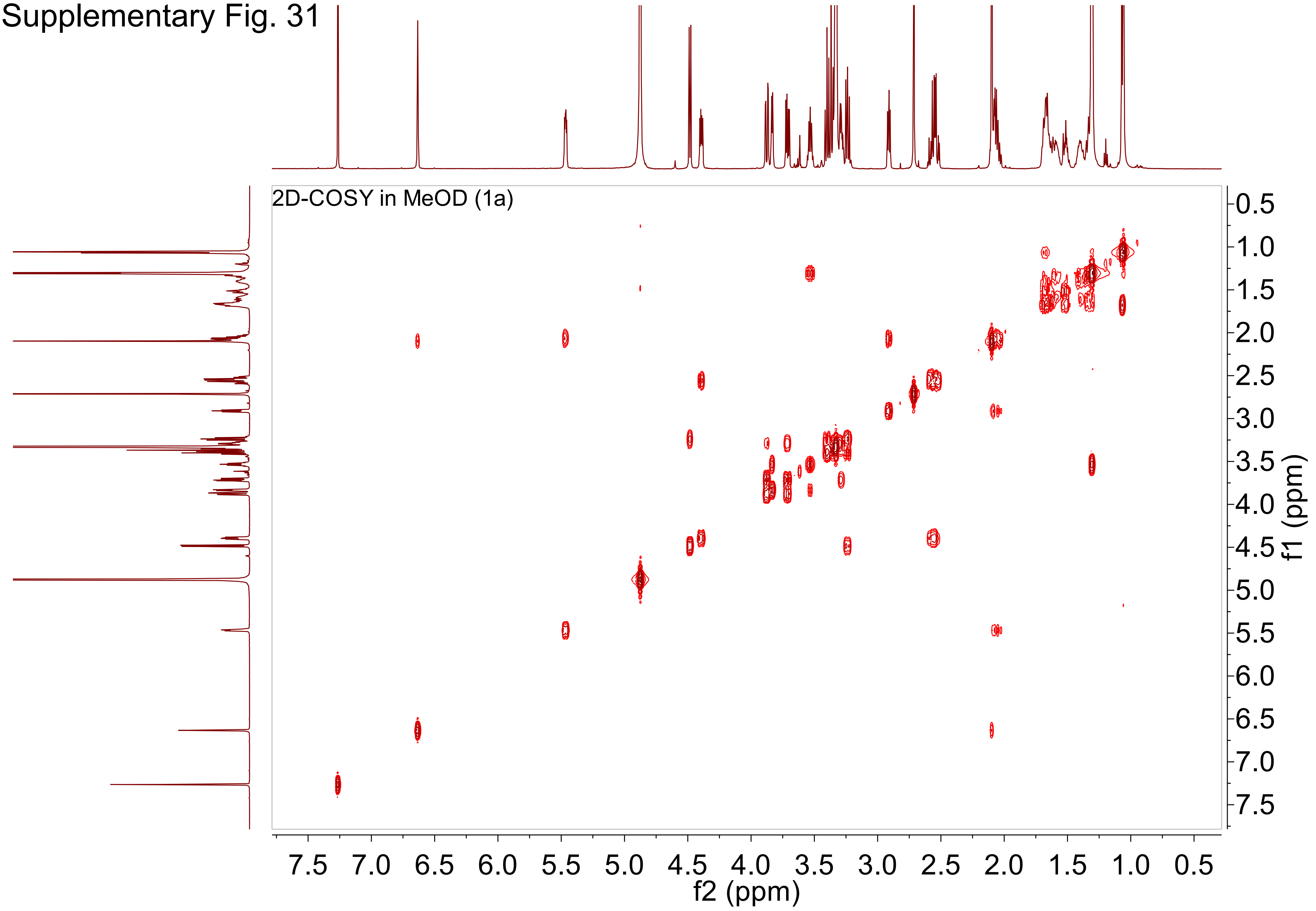

Supplementary Fig. 32

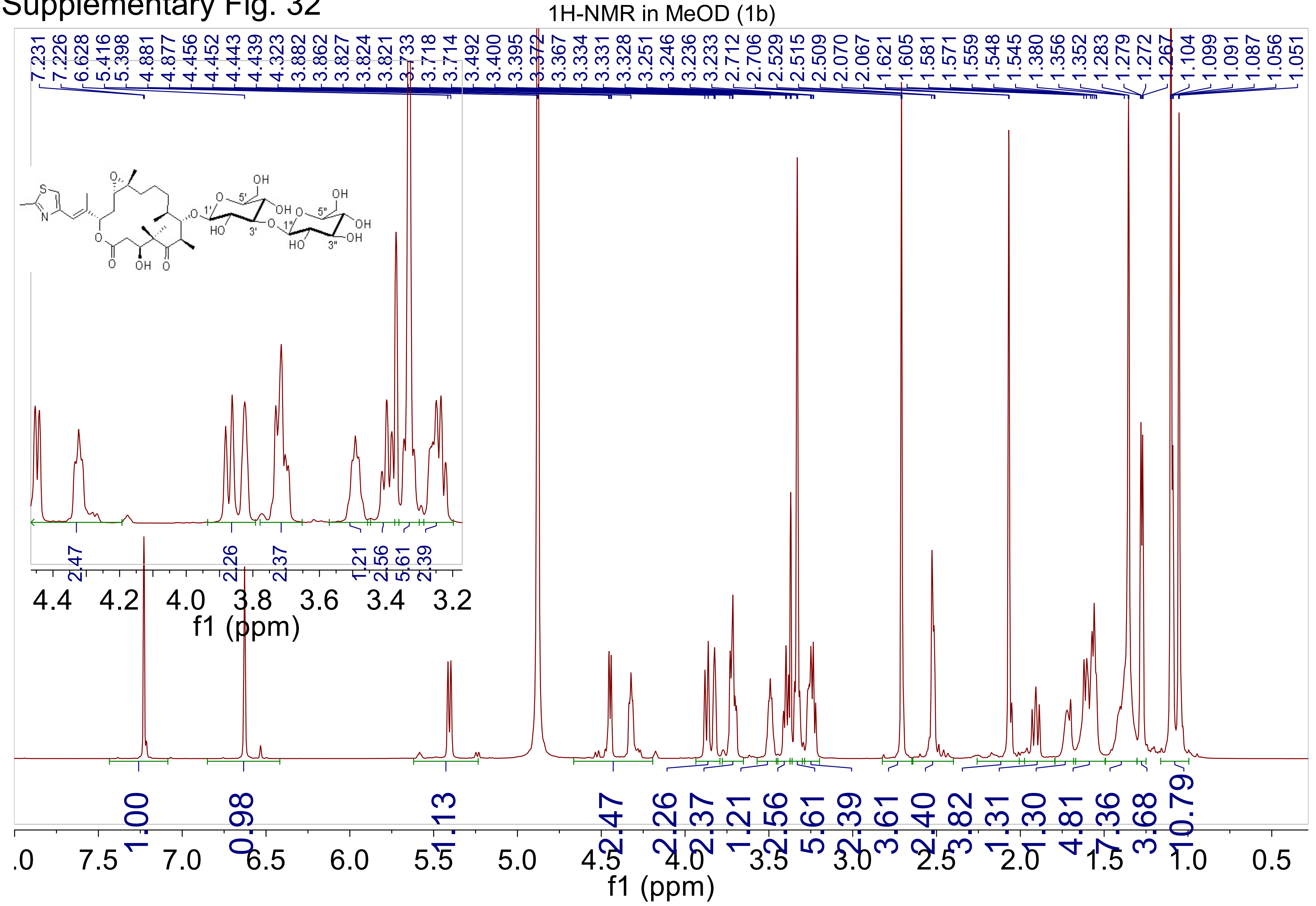

Supplementary Fig. 33

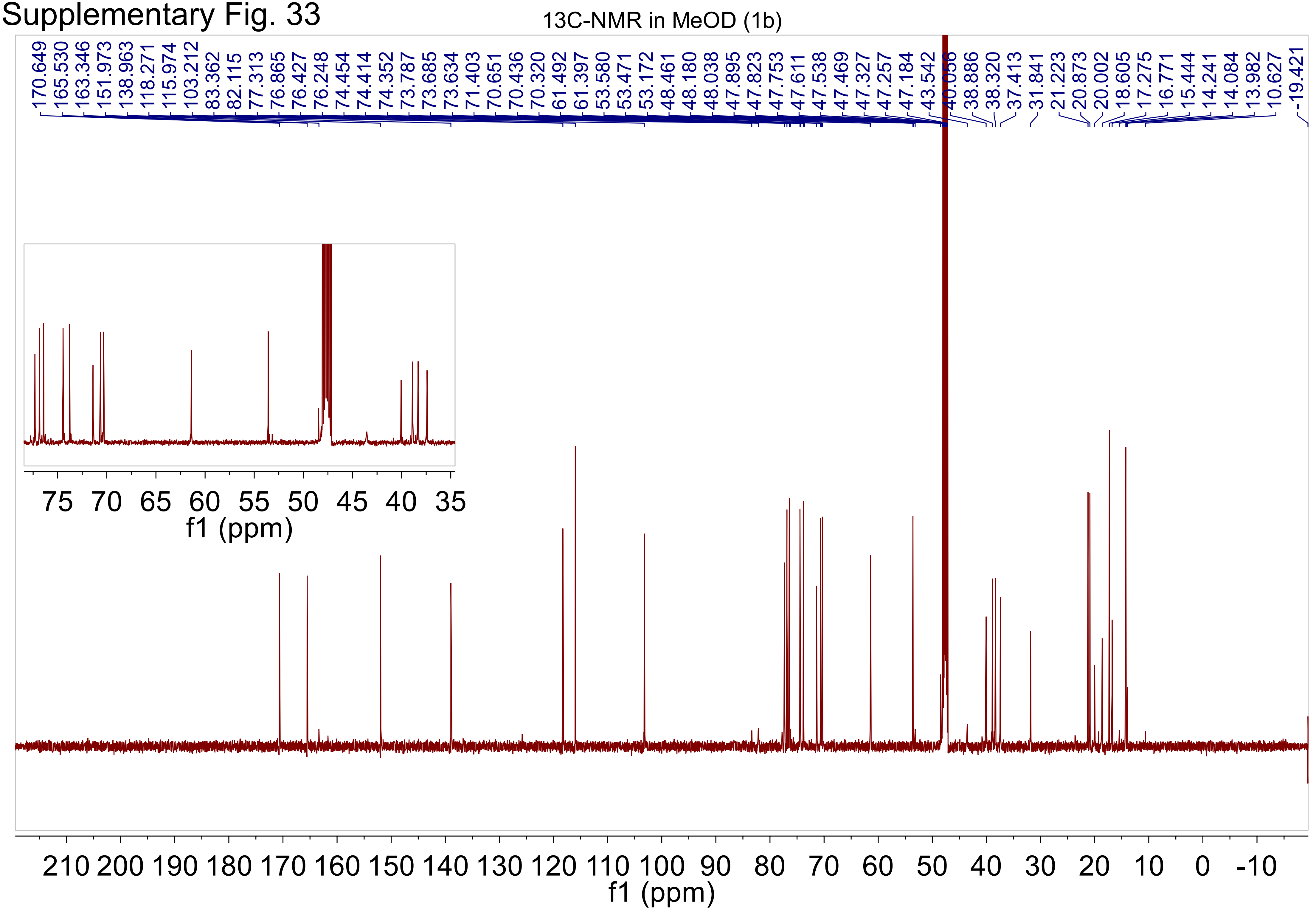

Supplementary Fig. 34

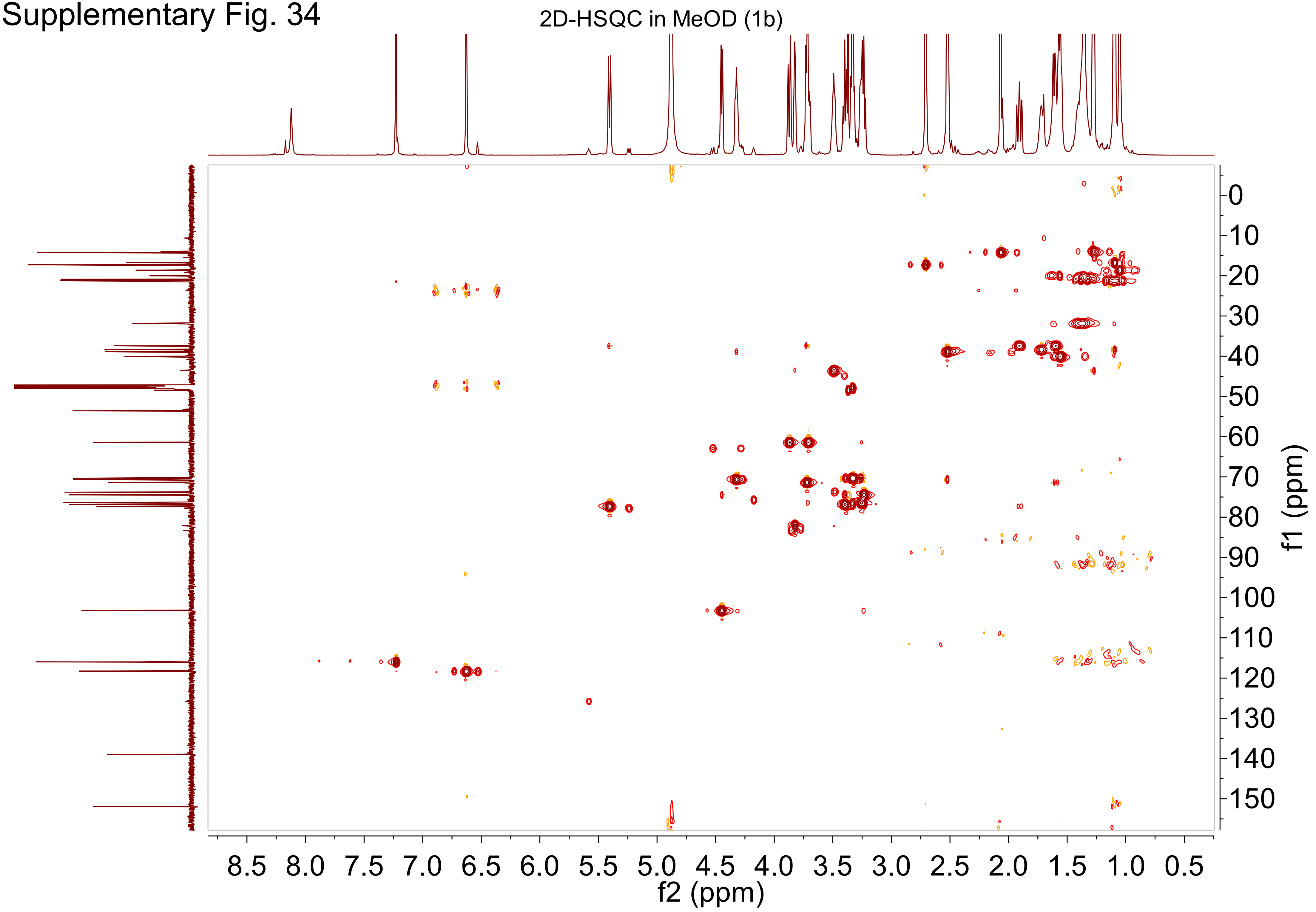

Supplementary Fig. 35

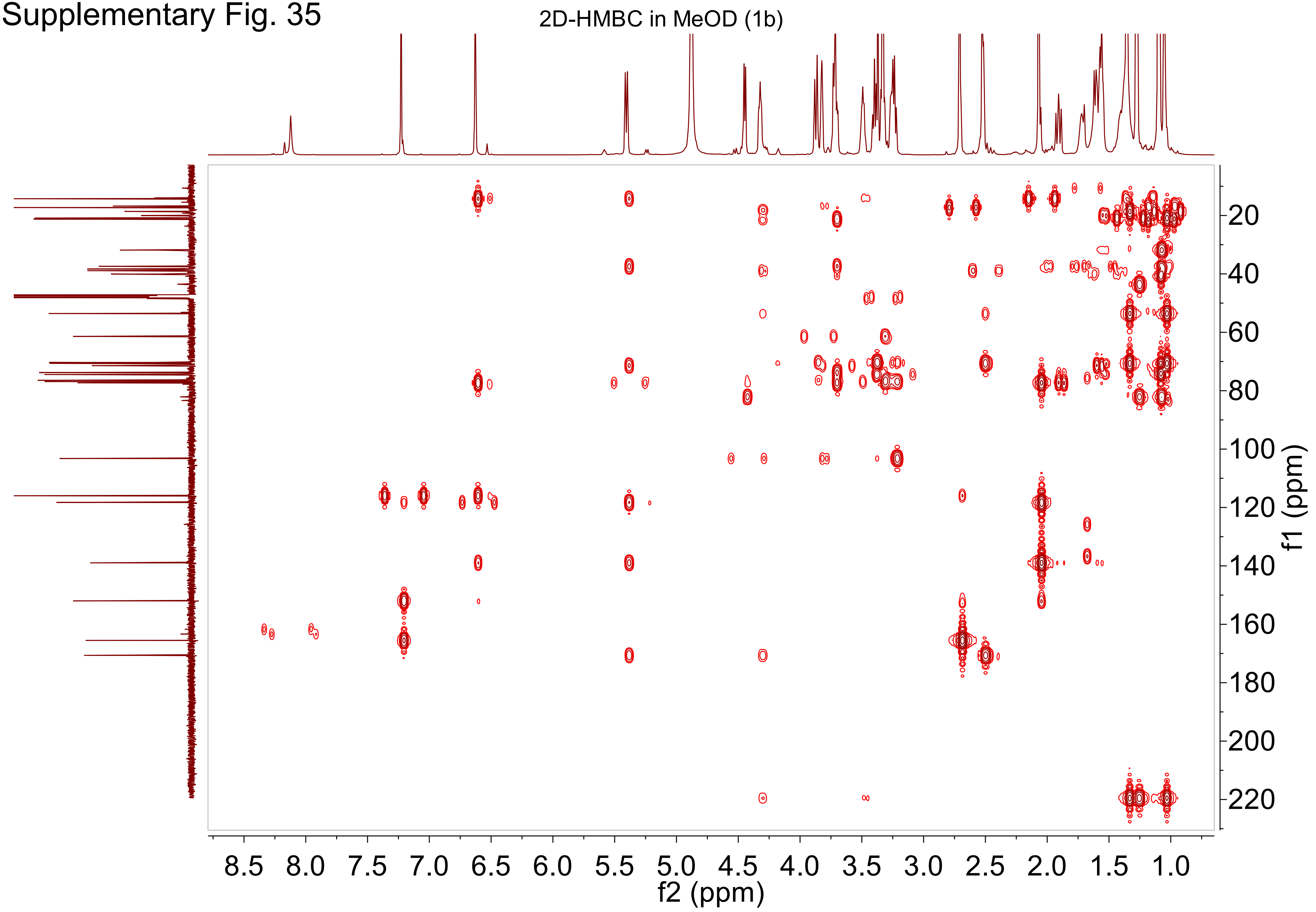

Supplementary Fig. 36

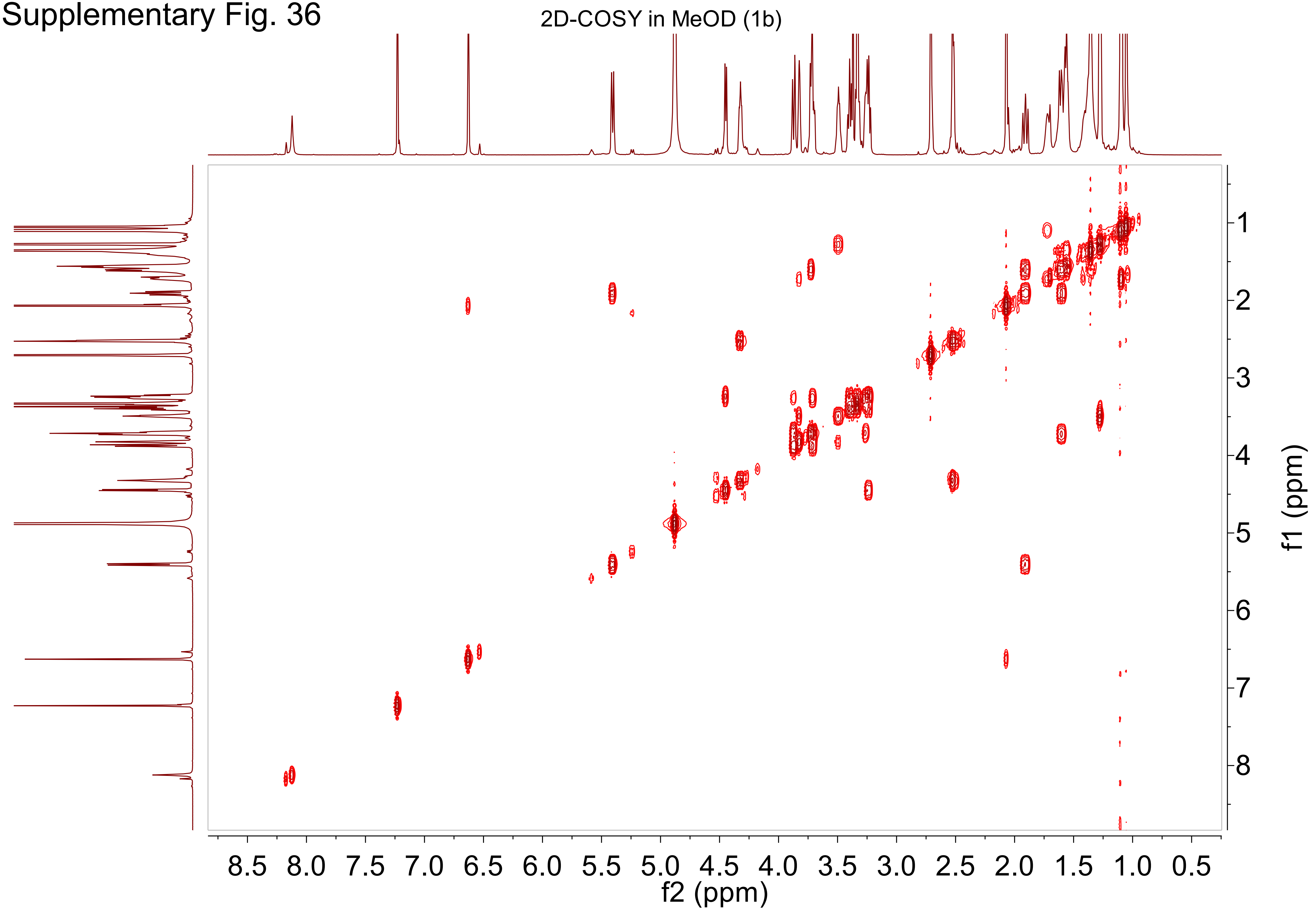

Supplementary Fig. 37

1H-NMR in MeOD (1c)

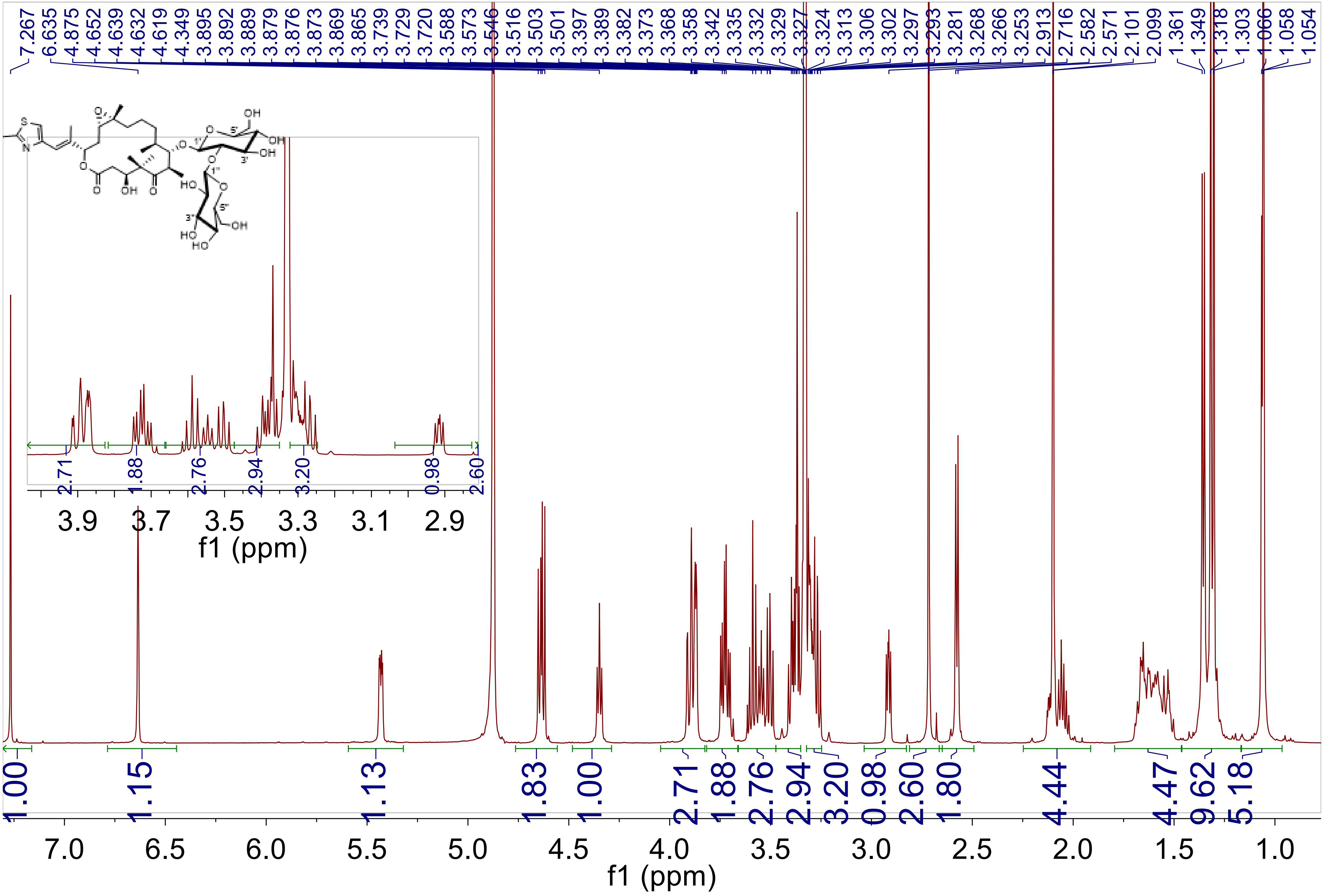

Supplementary Fig. 38

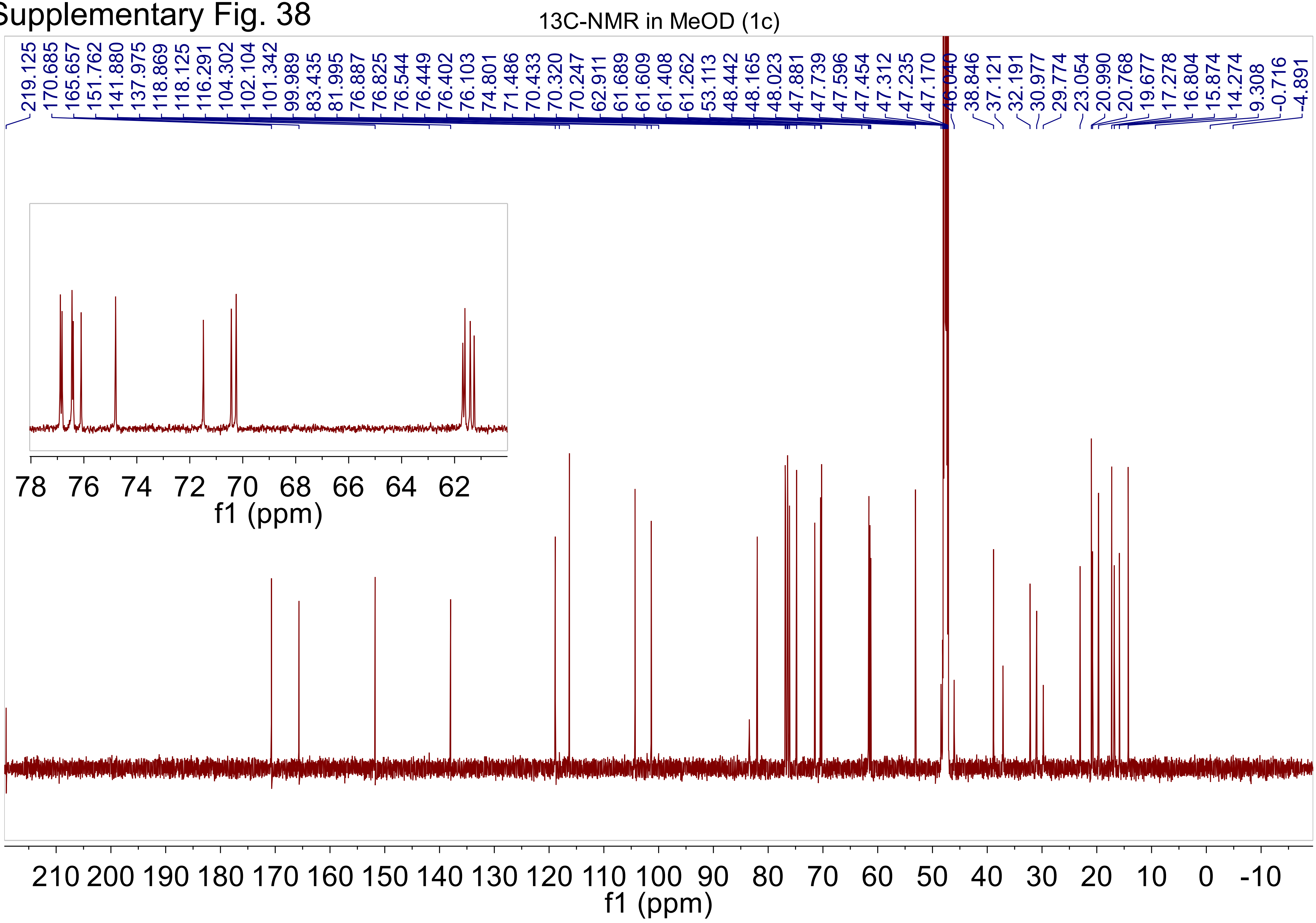

Supplementary Fig. 39

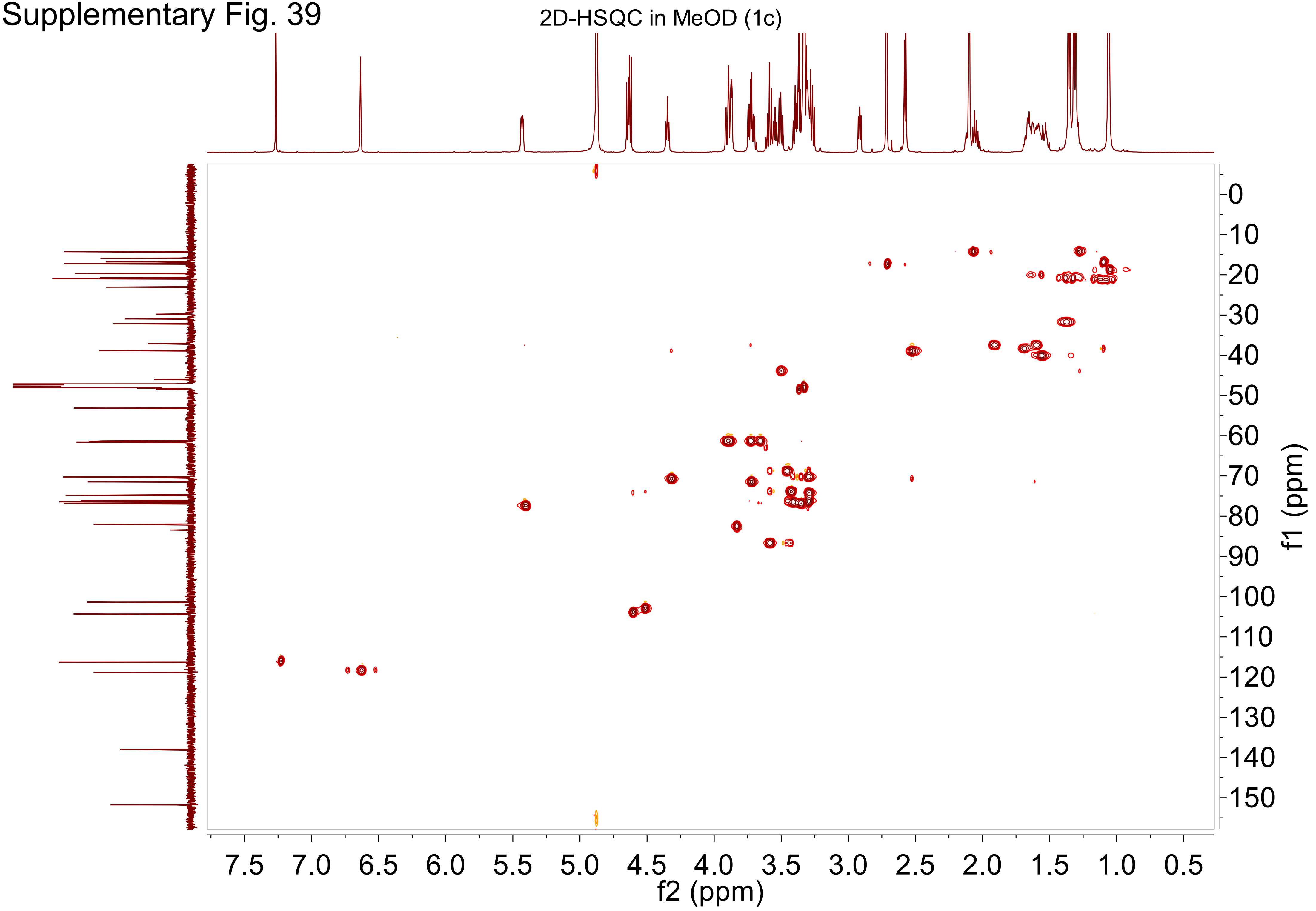

Supplementary Fig. 40

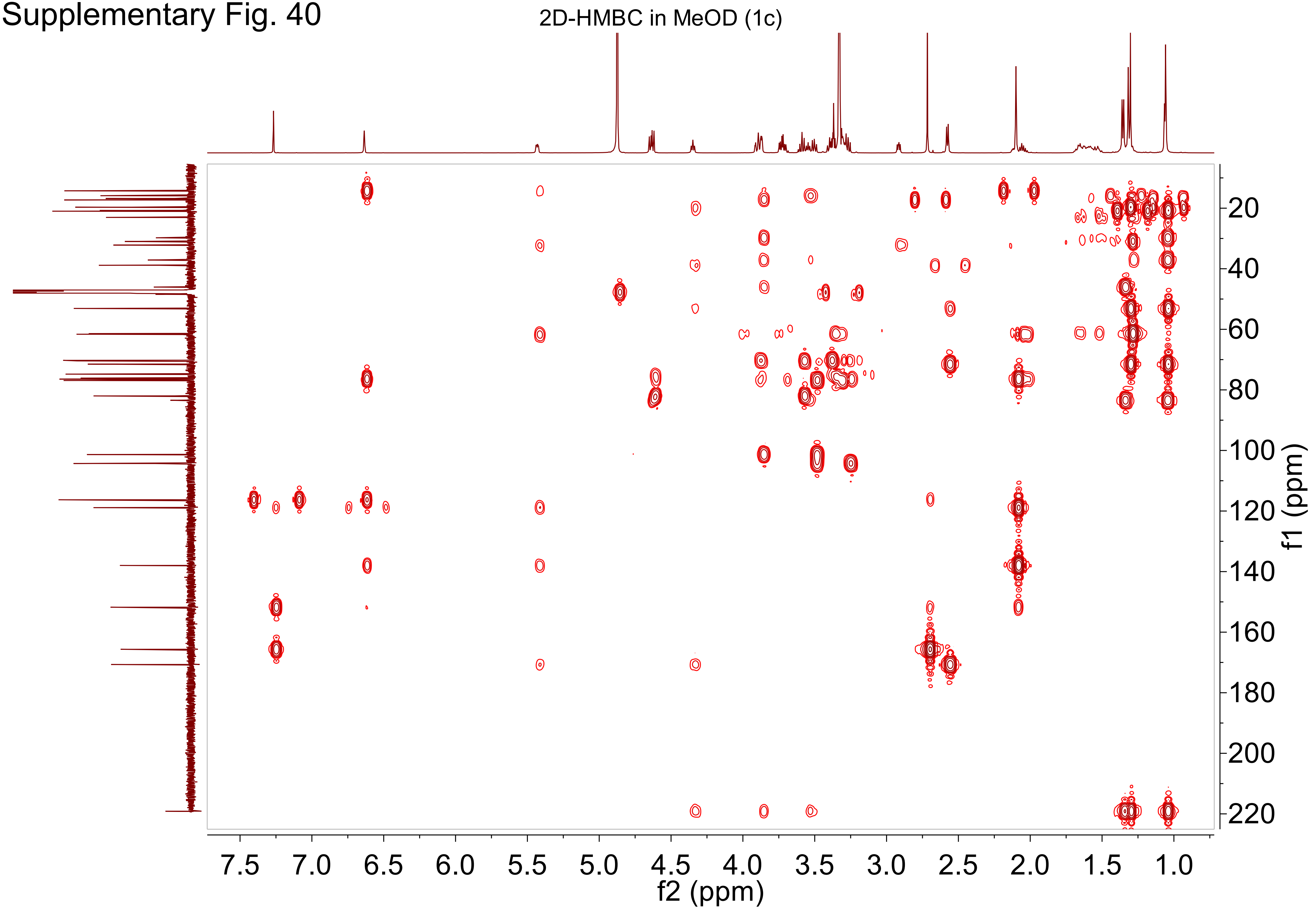

Supplementary Fig. 41

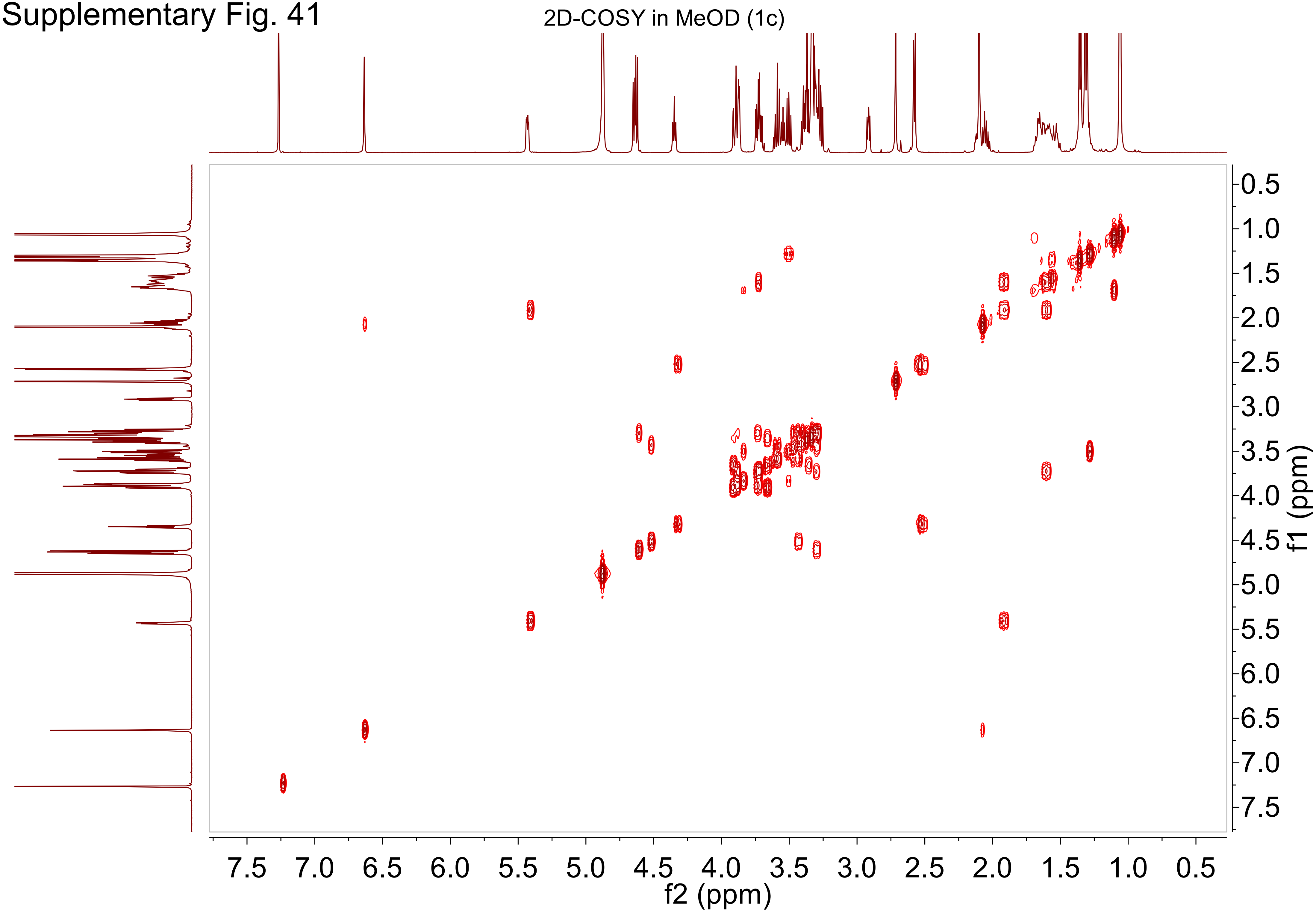

Supplementary Fig. 42

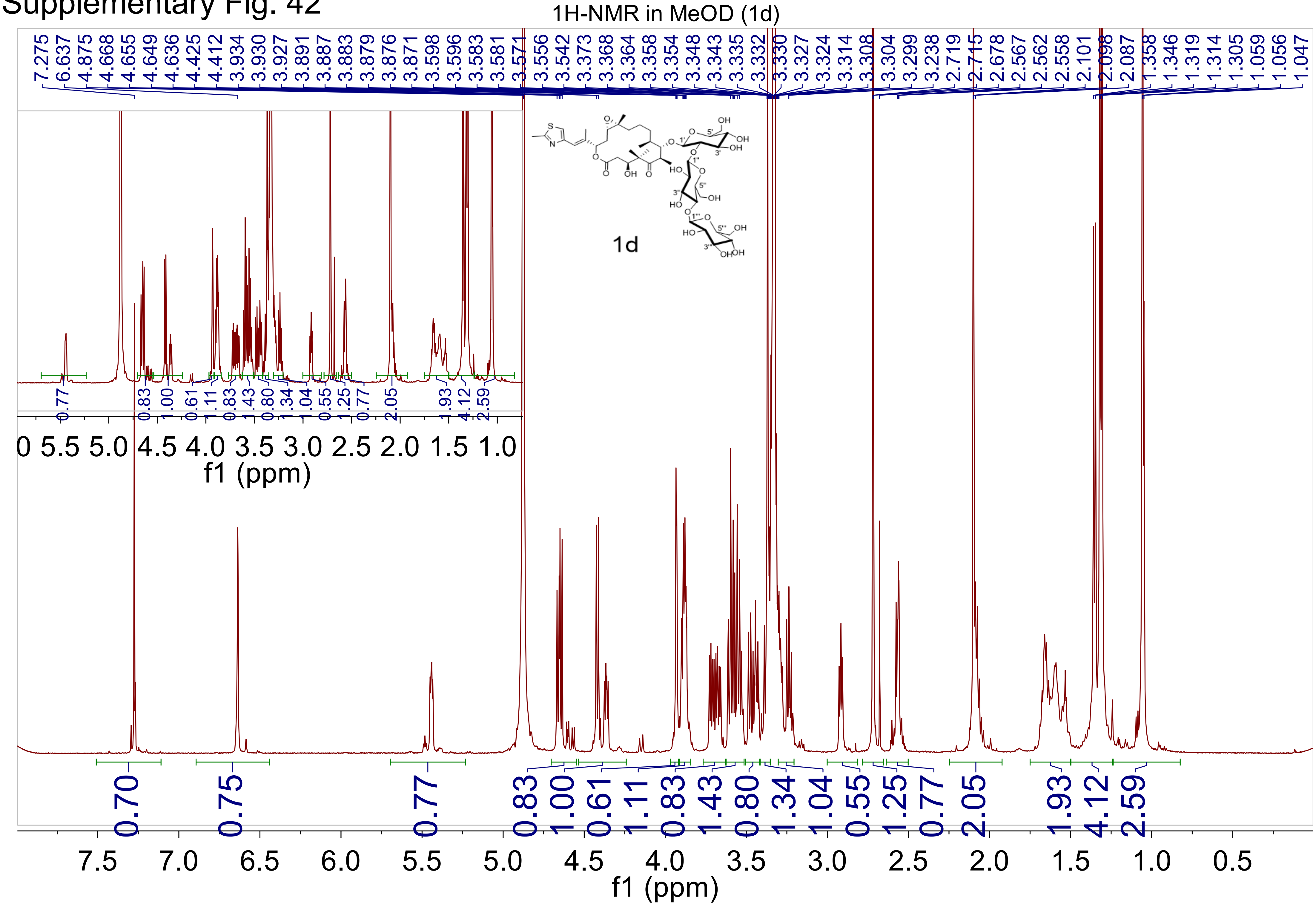

Supplementary Fig. 43

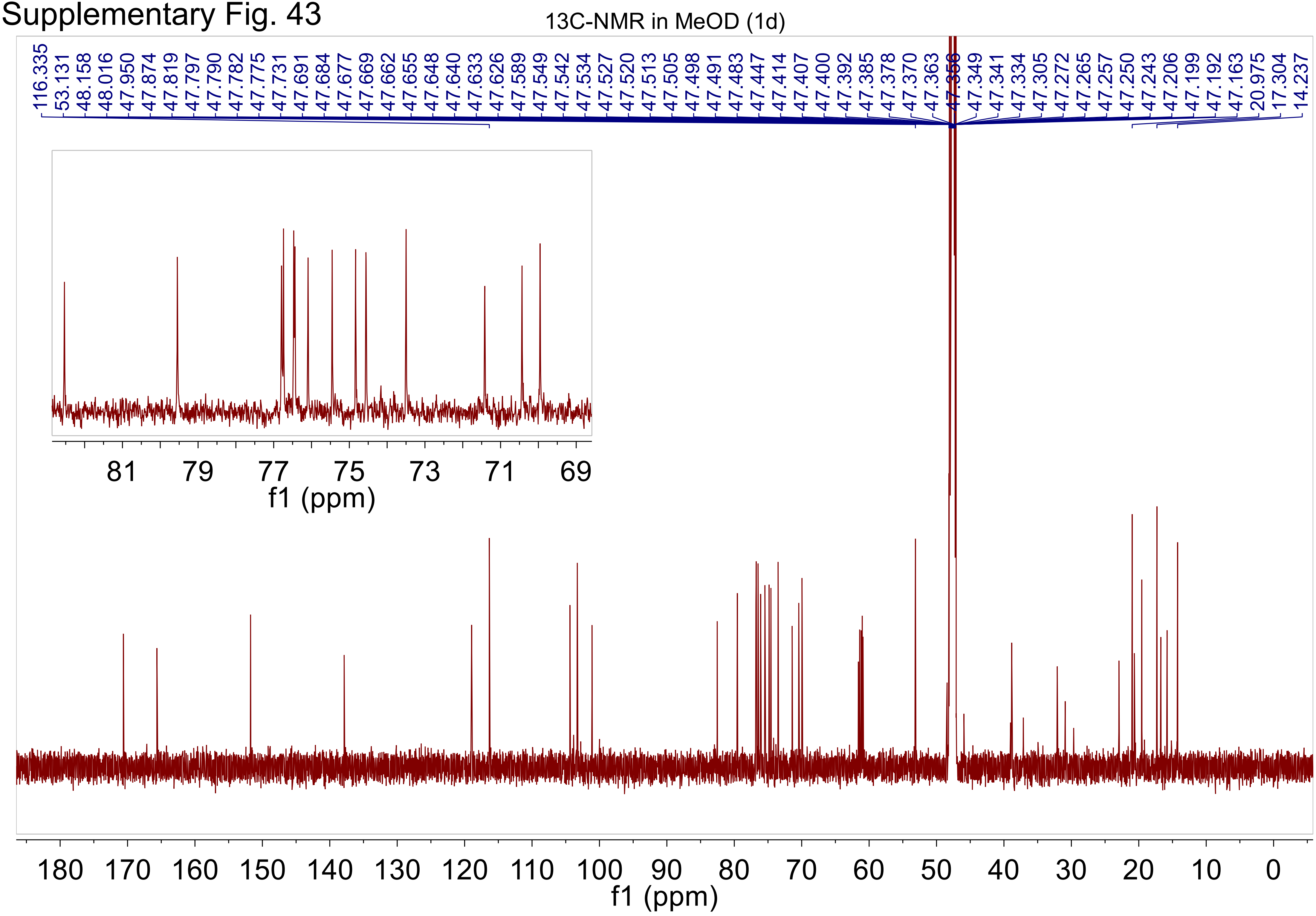

Supplementary Fig. 44

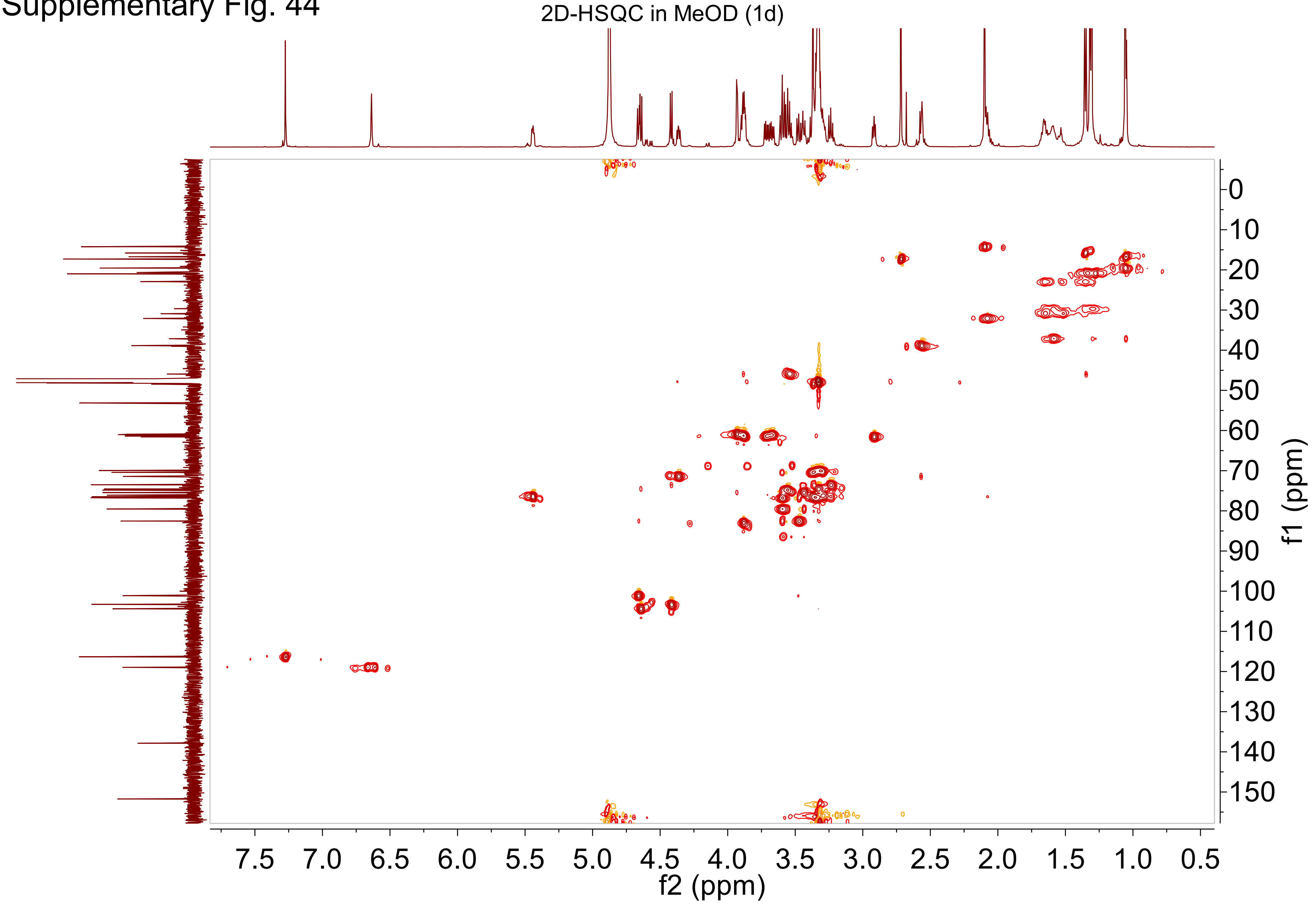

Supplementary Fig. 45

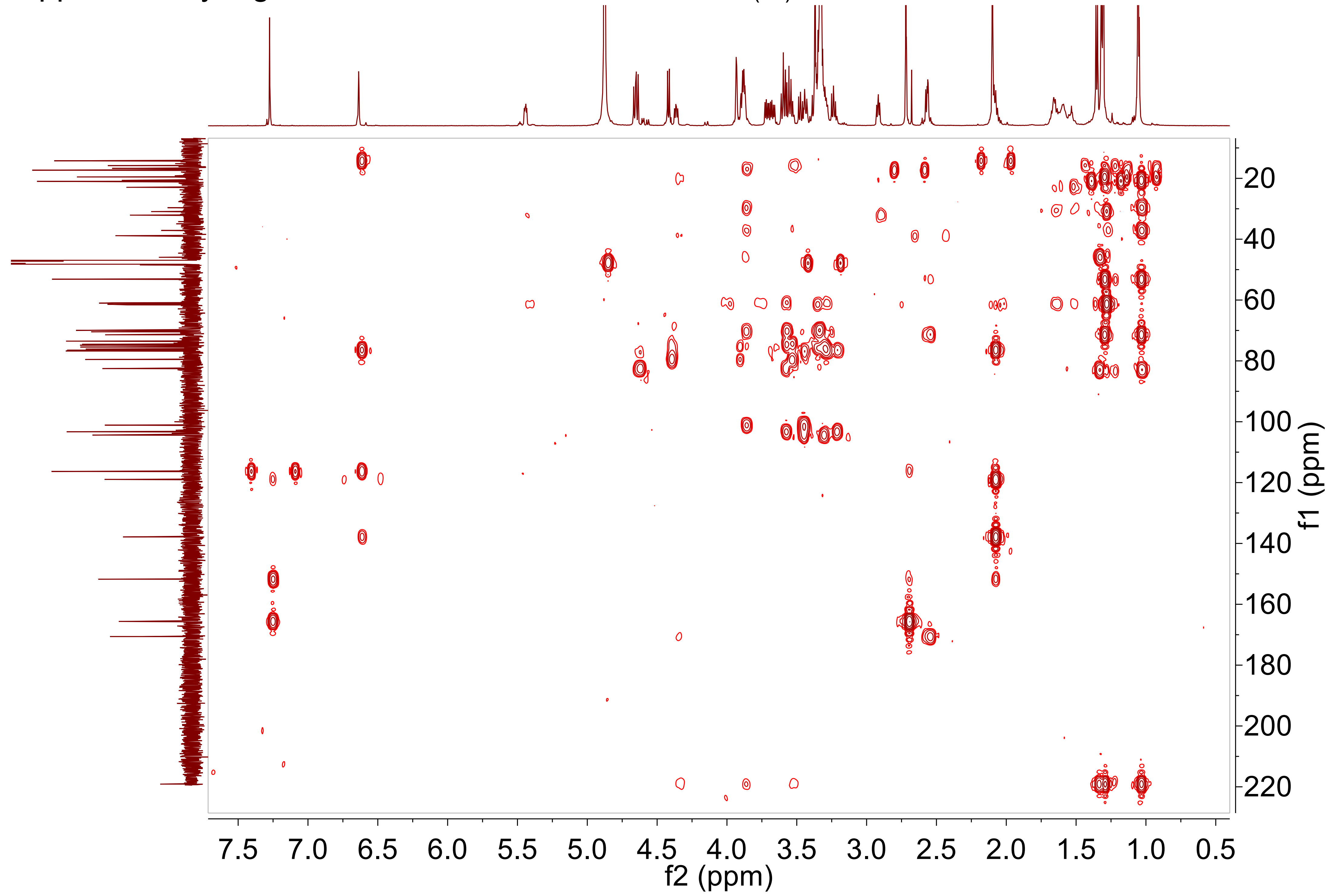

Supplementary Fig. 46

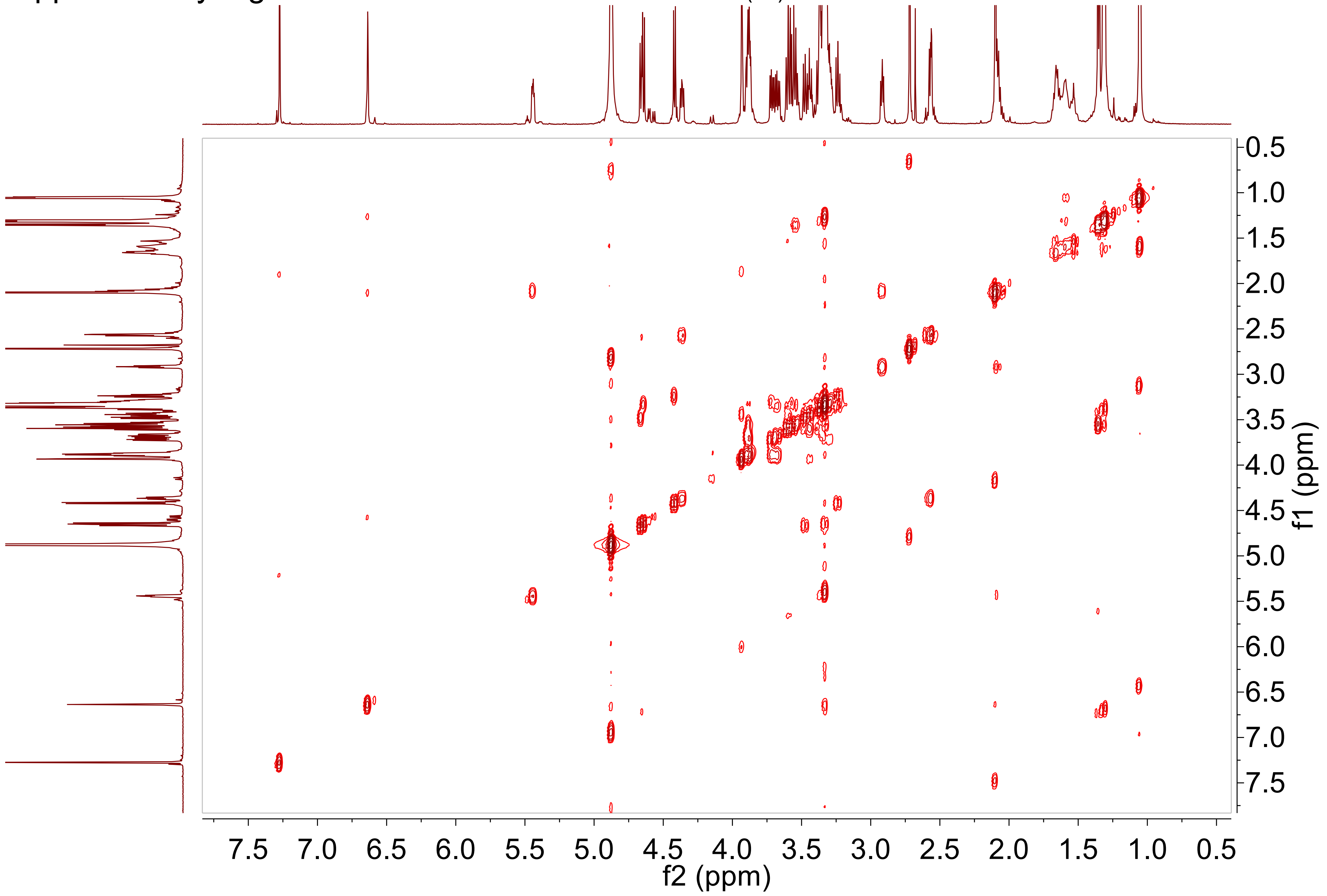

Supplementary Fig. 47

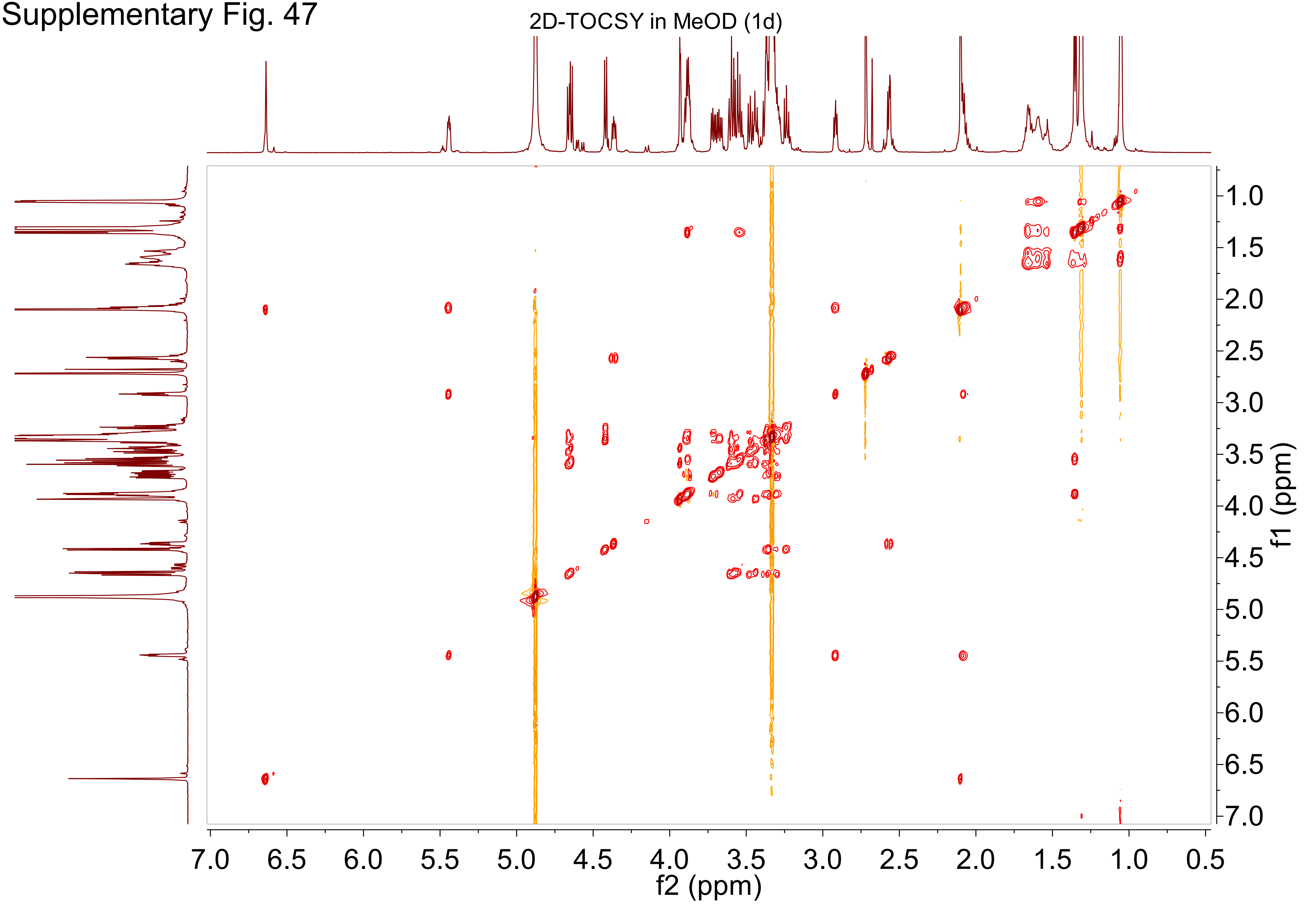

Supplementary Fig. 48

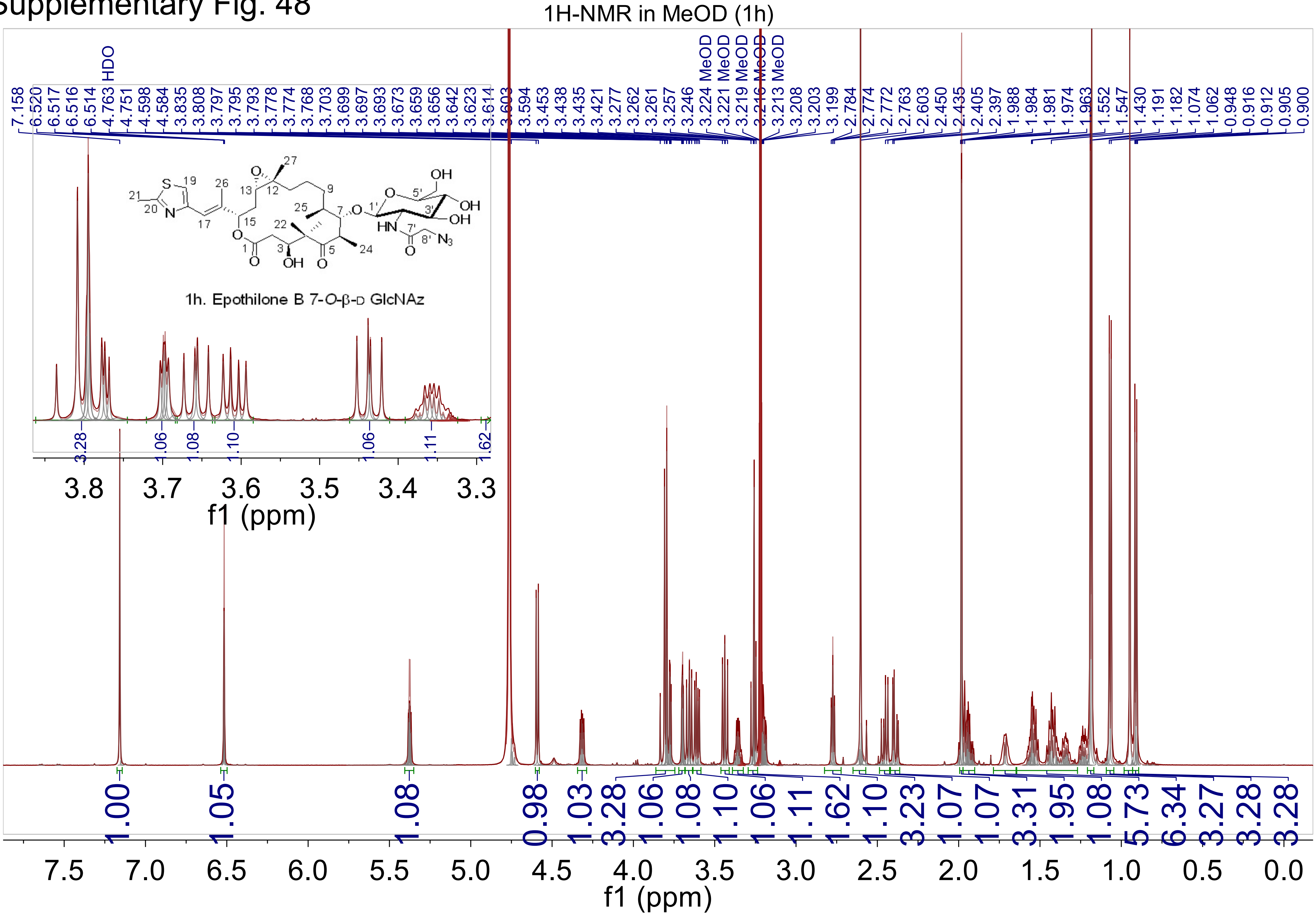

Supplementary Fig. 49

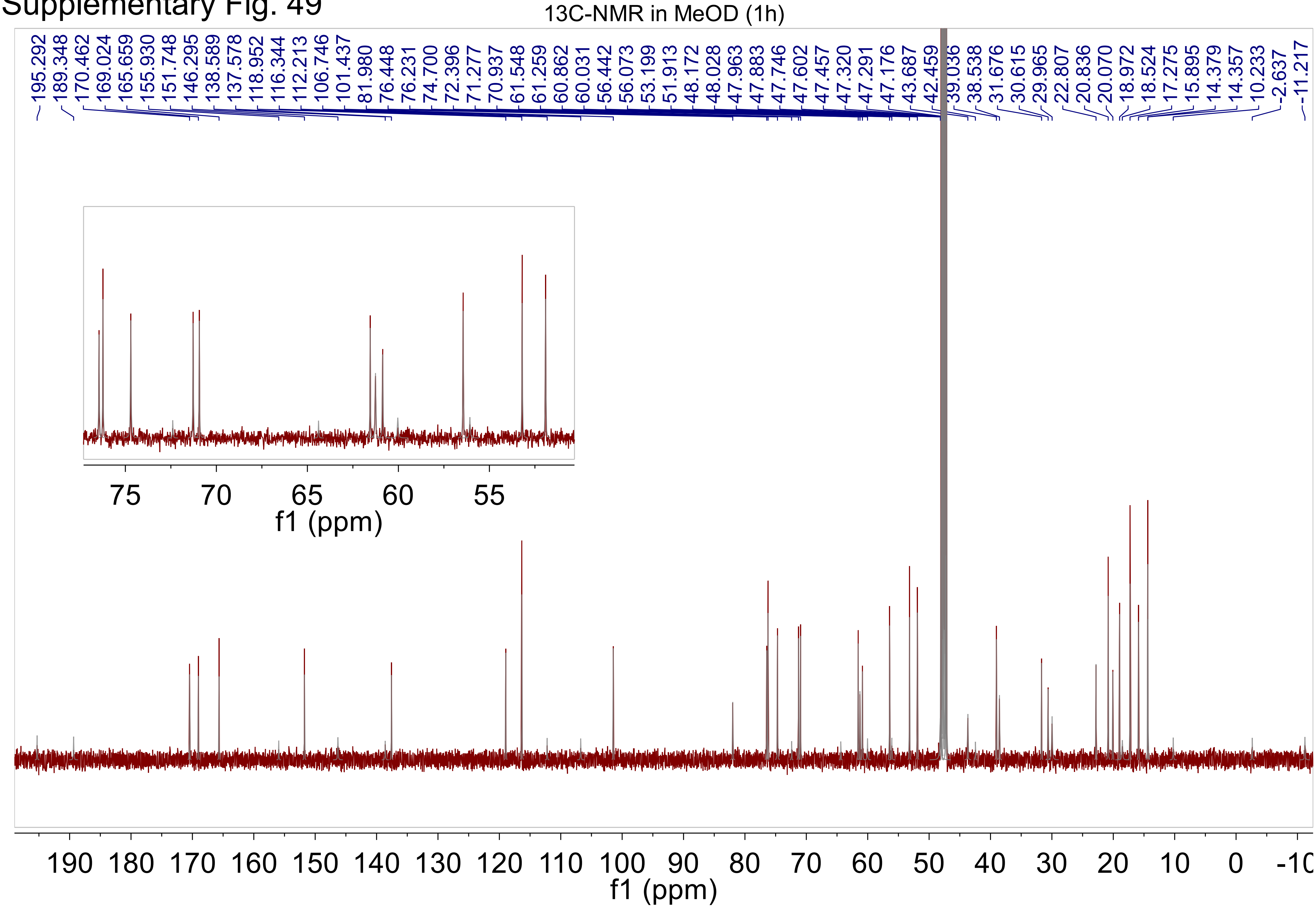

Supplementary Fig. 50

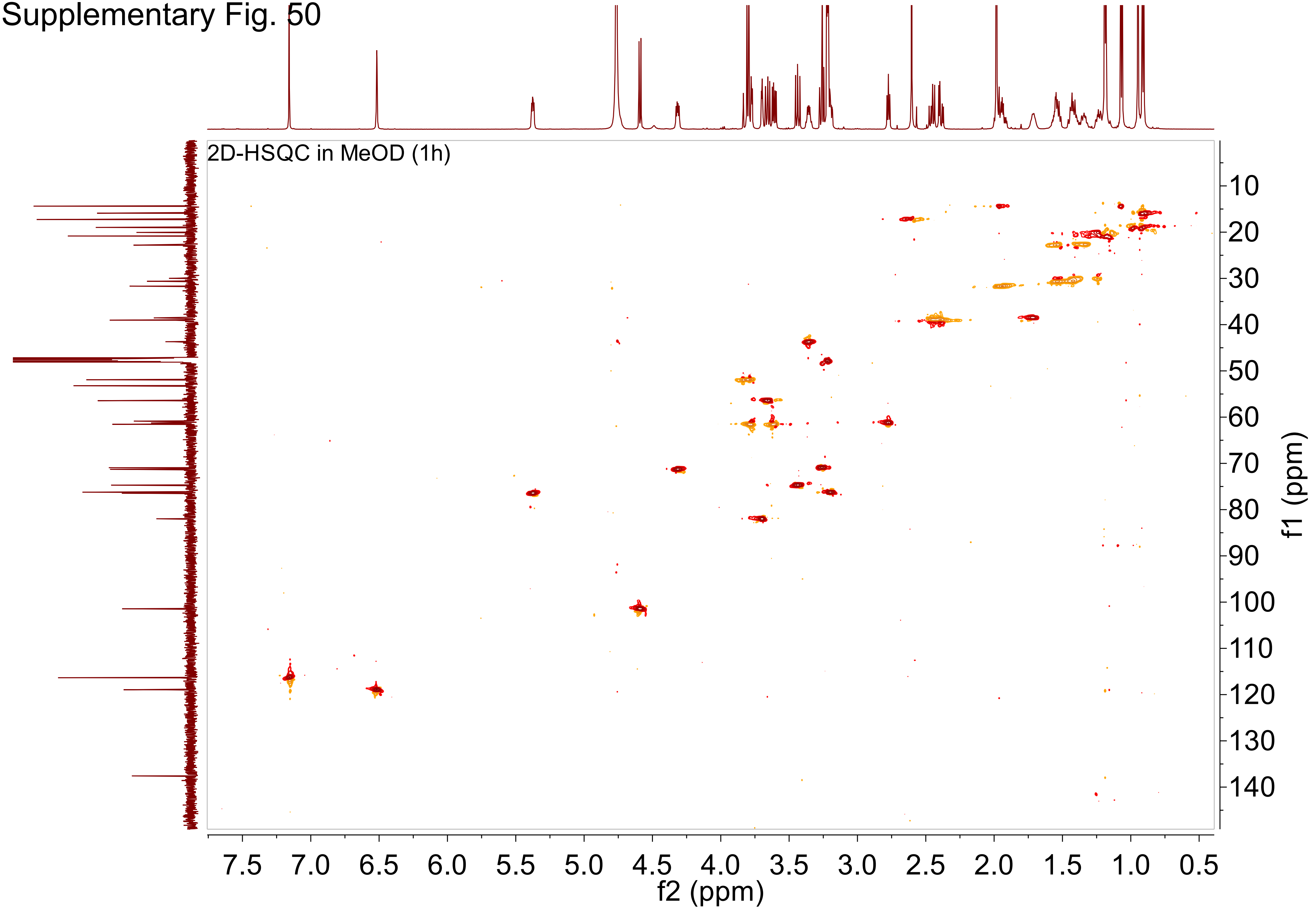

Supplementary Fig. 51

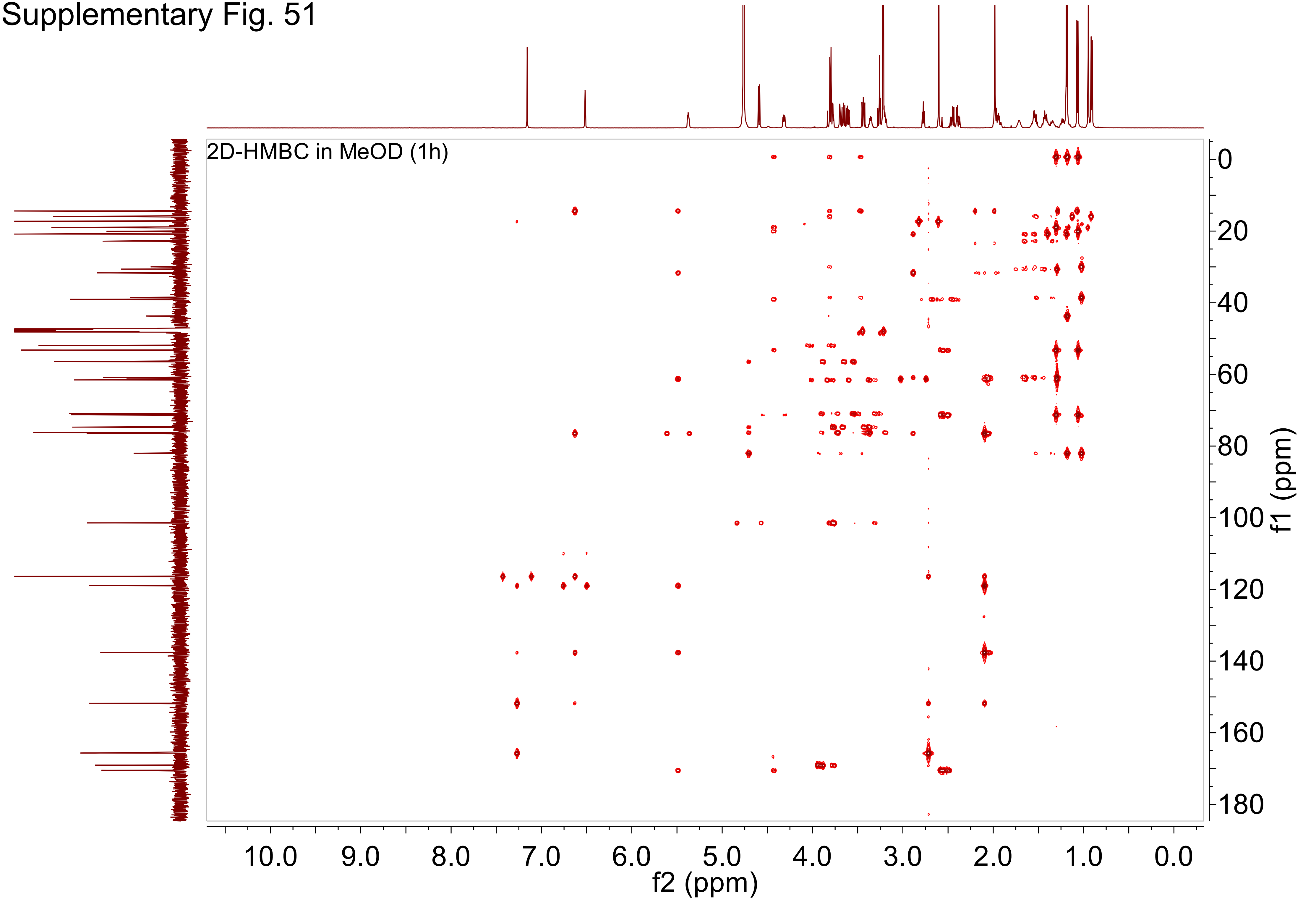

Supplementary Fig. 52

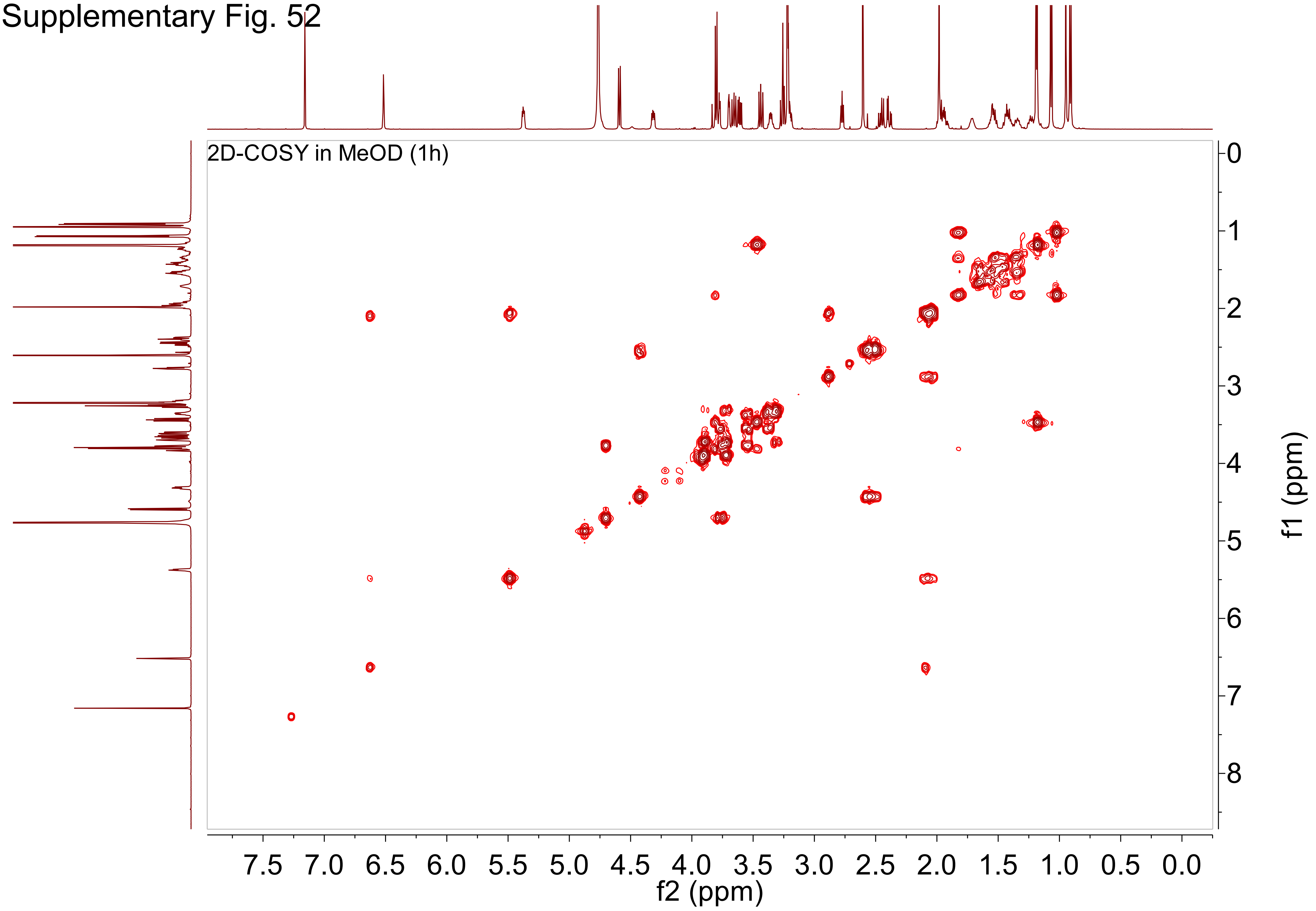

Supplementary Fig. 53

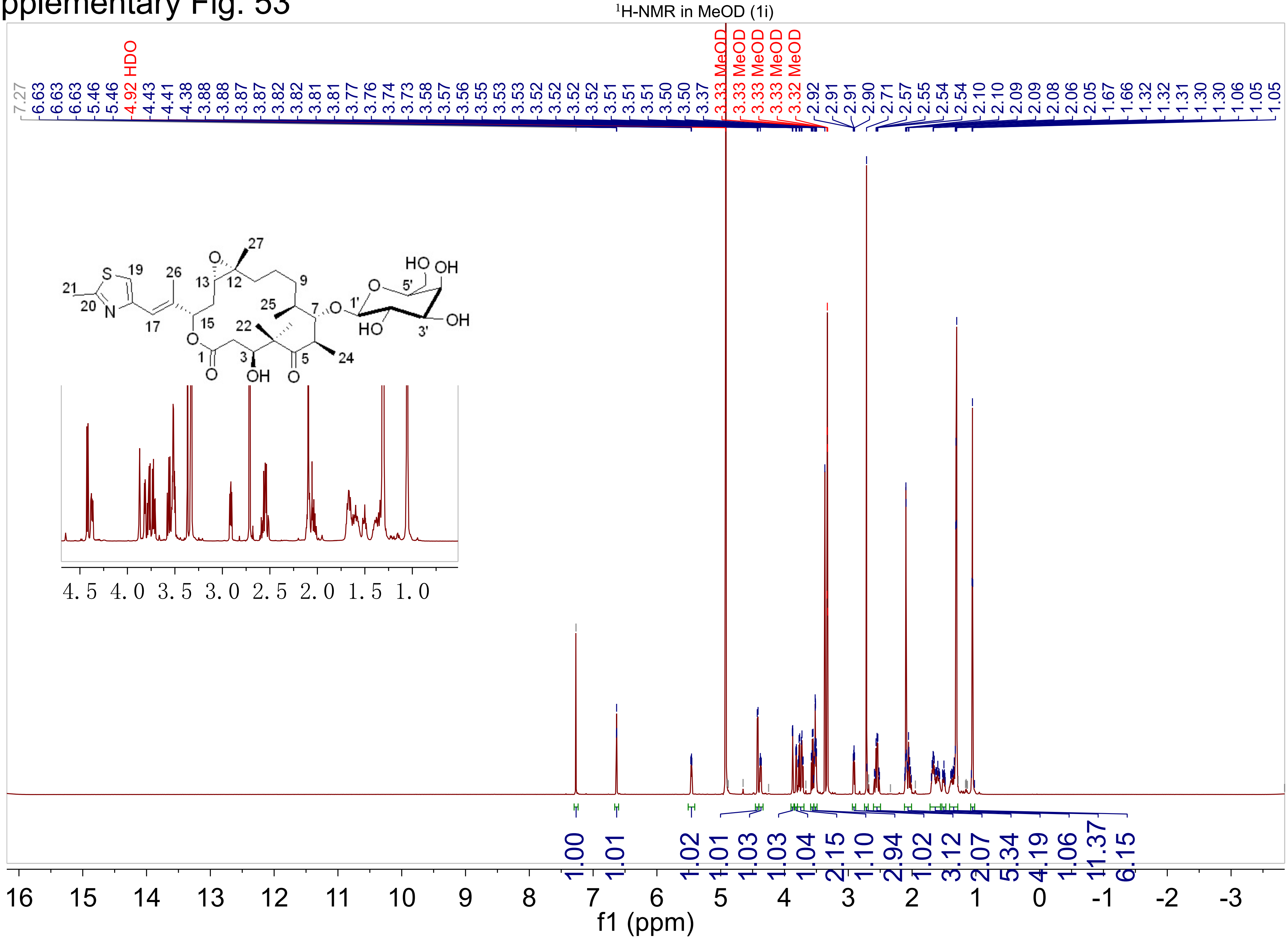

Supplementary Fig. 54

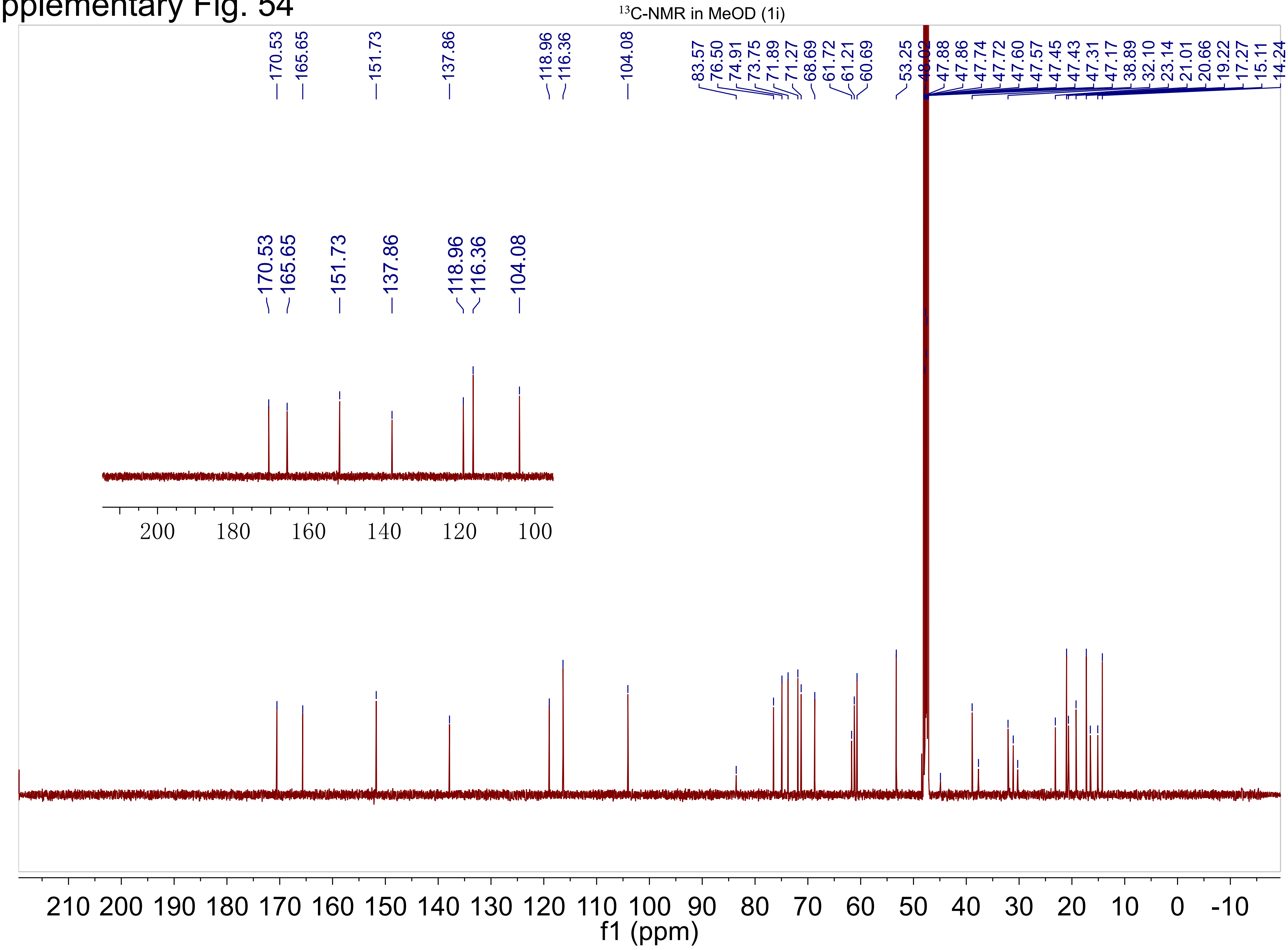

Supplementary Fig. 55

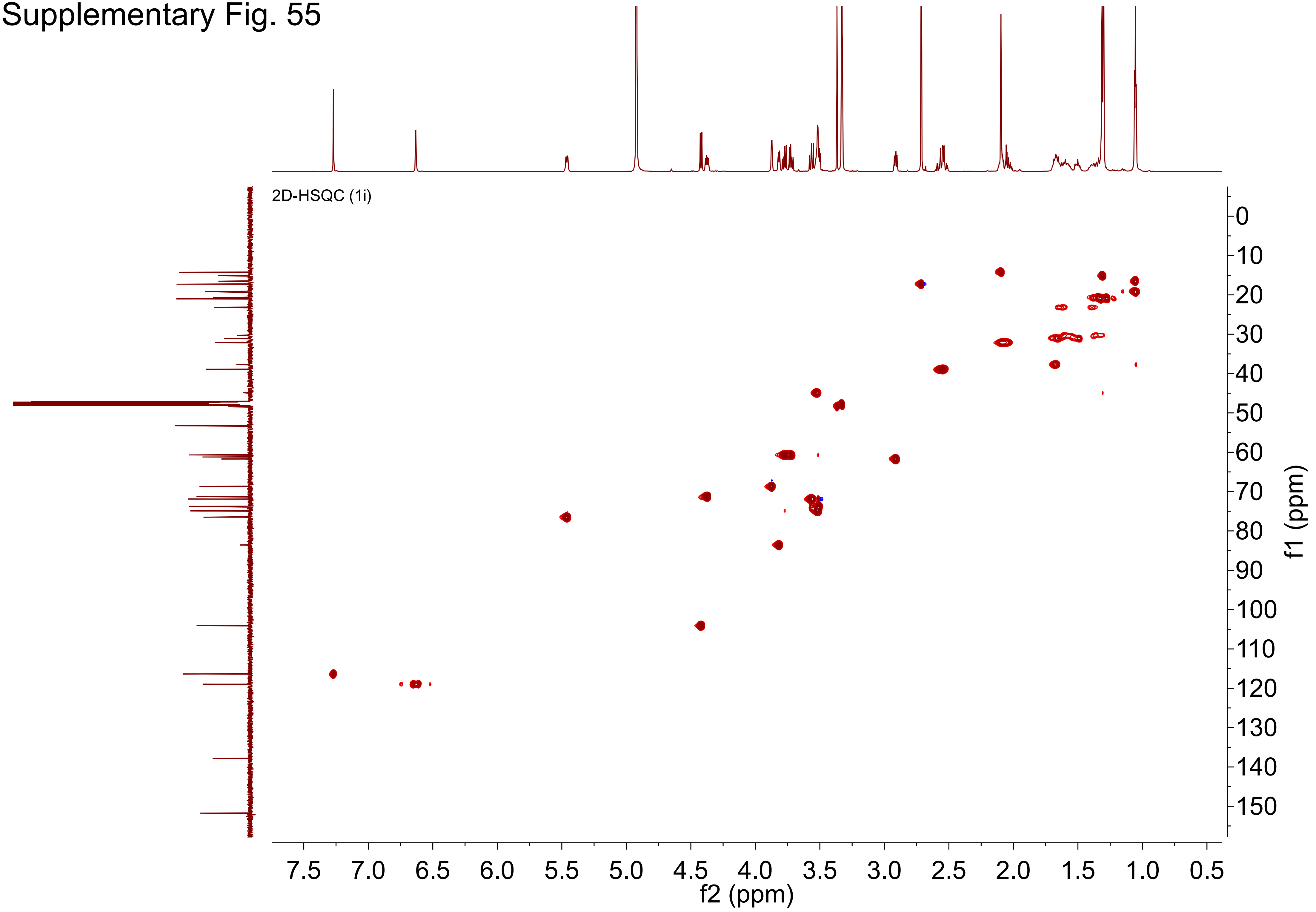

Supplementary Fig. 56

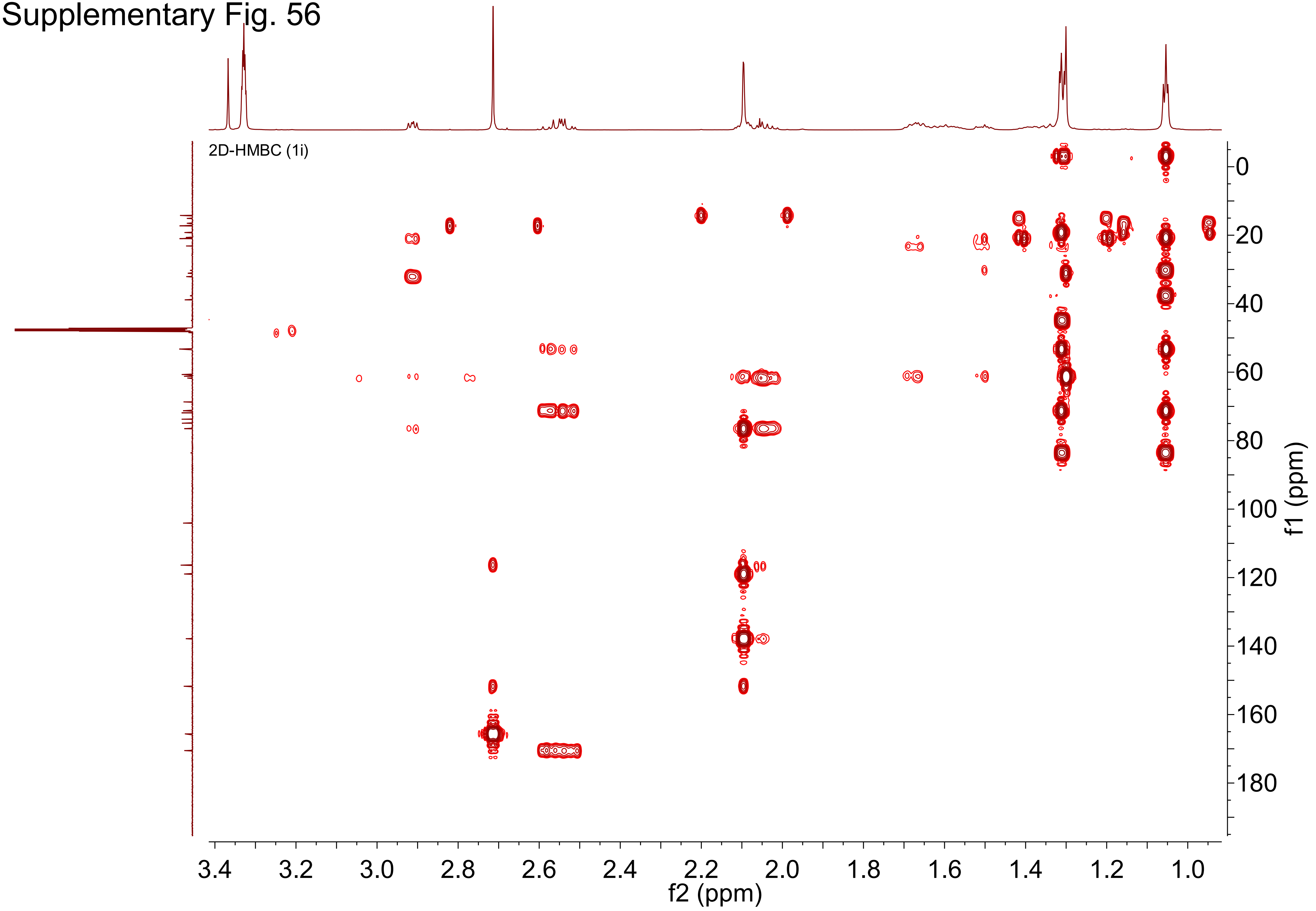

Supplementary Fig. 57

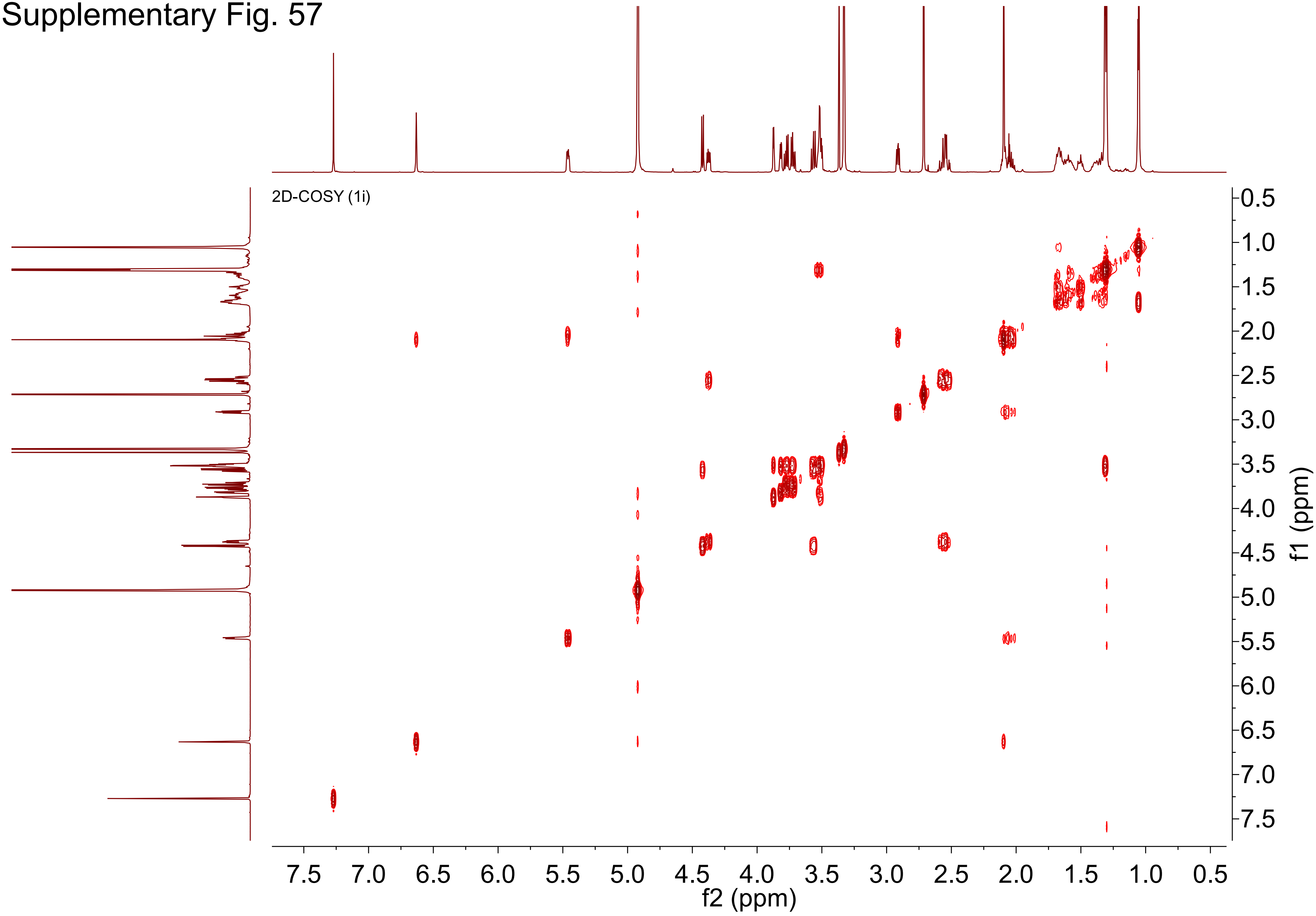

Supplementary Fig. 58

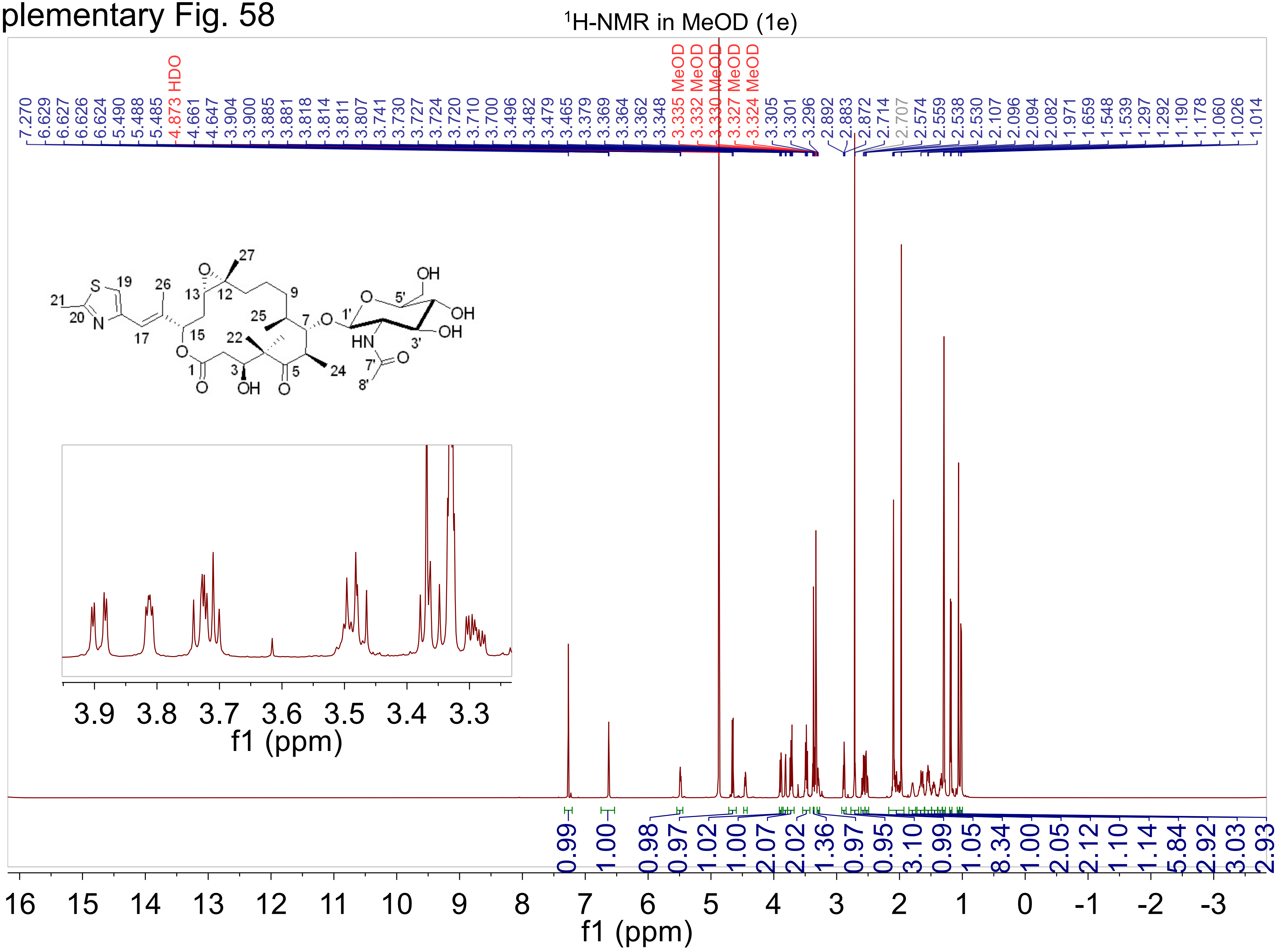

Supplementary Fig. 59

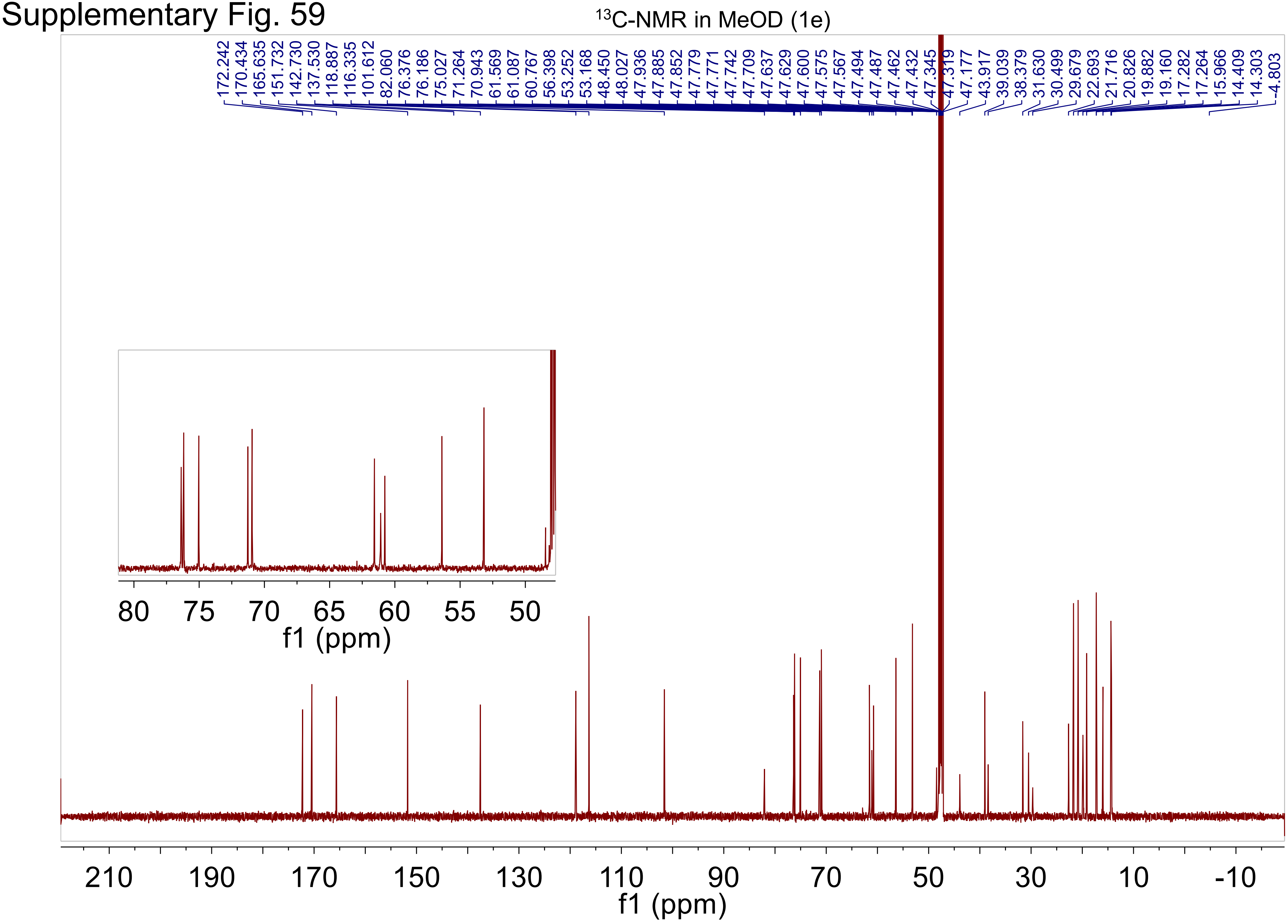

Supplementary Fig. 60

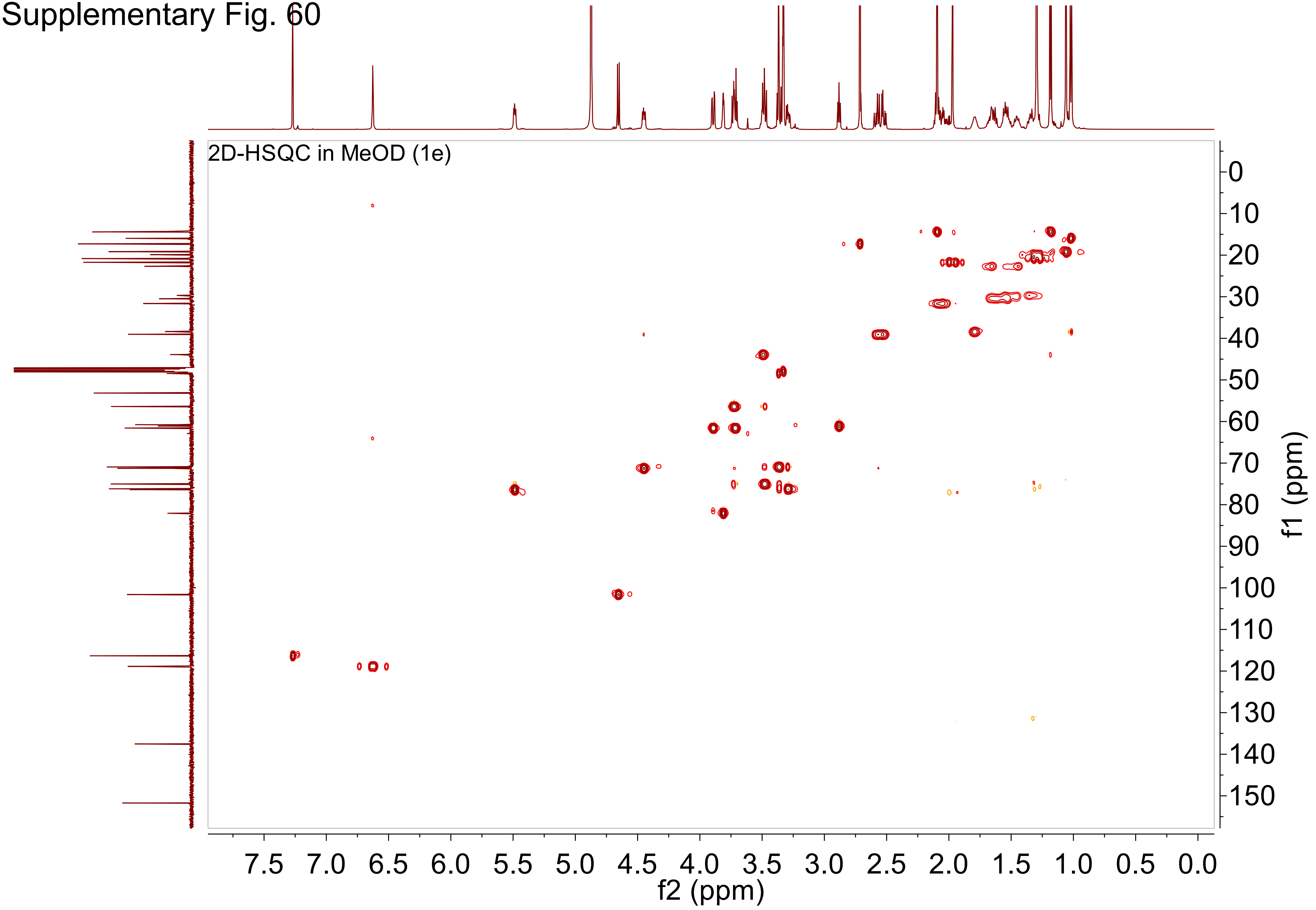

Supplementary Fig. 61

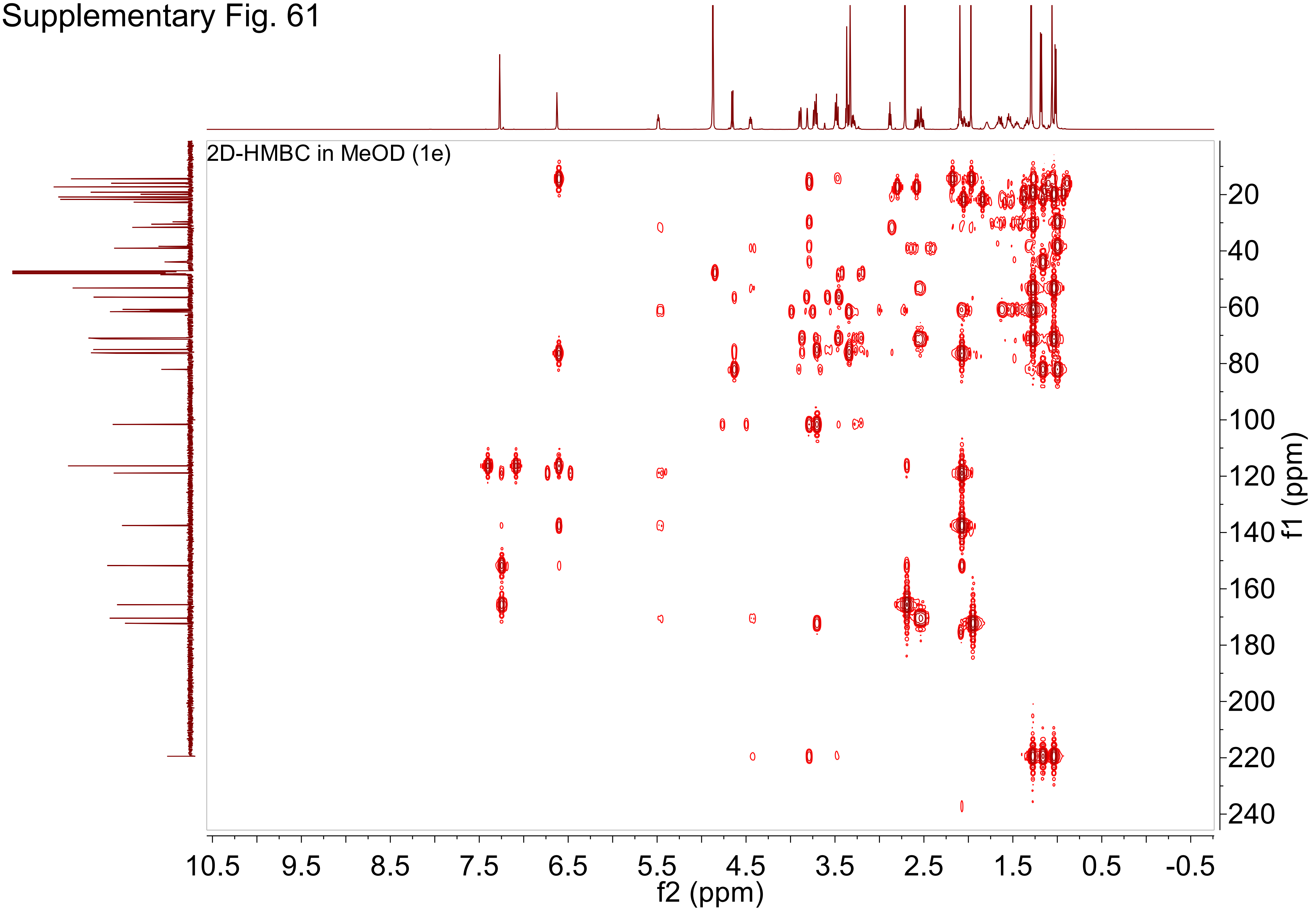

Supplementary Fig. 62

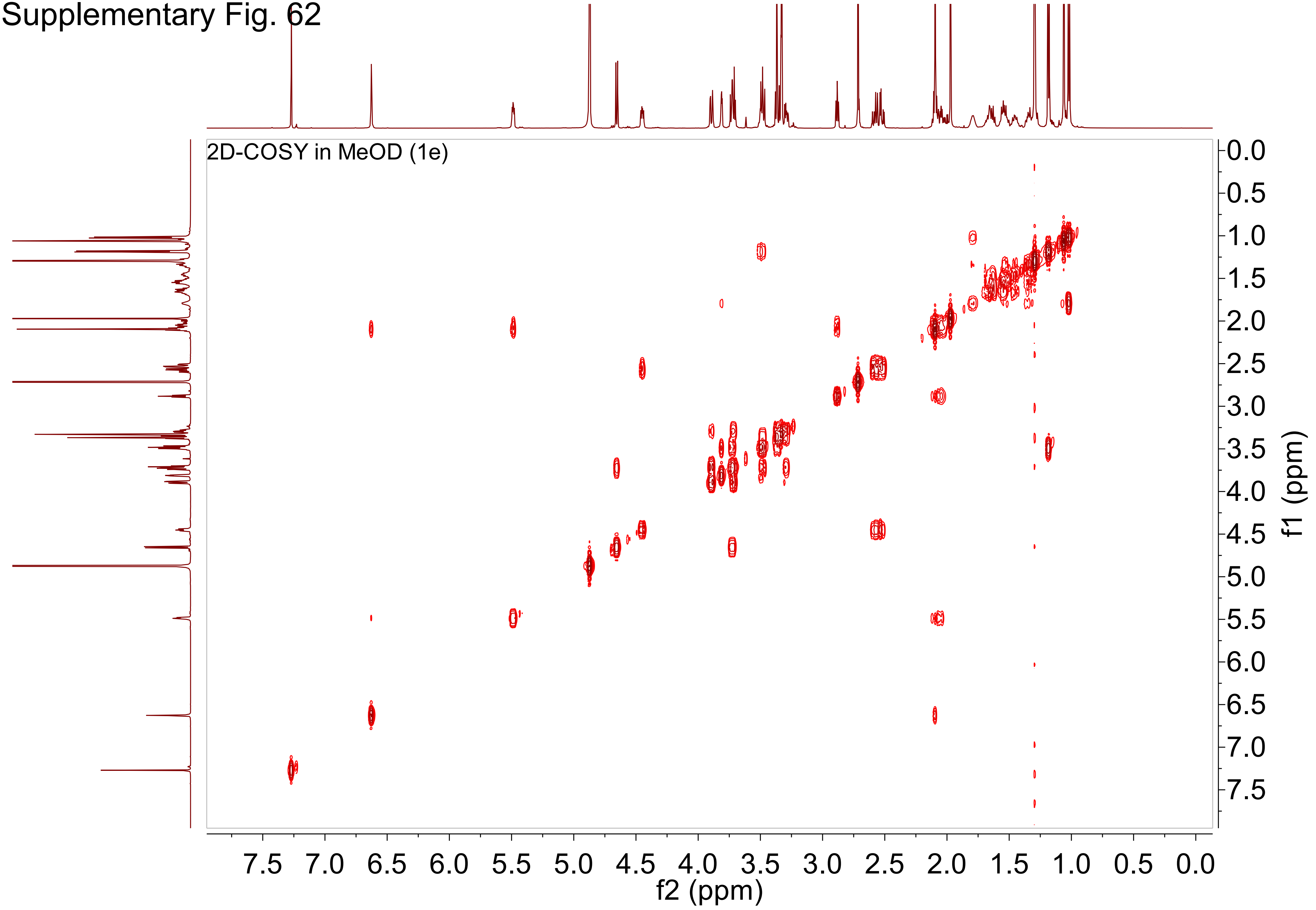

Supplement: Supplementary file 1 — Supplemental Material [file 42003_2022_3047_MOESM1_ESM.pdf]
