# Supplementary material for: Docking-guided rational engineering of a macrolide glycosyltransferase glycodiversifies epothilone B
Source: Commun Biol. 2022 Jan 27;5:100. doi: 10.1038/s42003-022-03047-y (PMC8795383; doi:10.1038/s42003-022-03047-y)
Supplement: Supplementary file 2 — Description of Additional Supplementary Files [file 42003_2022_3047_MOESM2_ESM.pdf]

Description of Additional Supplementary Files

File name: Supplementary Data 1

Description: Source data for the graphs in the main figures.
