# Supplementary material for: Docking-guided rational engineering of a macrolide glycosyltransferase glycodiversifies epothilone B
Source: Commun Biol. 2022 Jan 27;5:100. doi: 10.1038/s42003-022-03047-y (PMC8795383; doi:10.1038/s42003-022-03047-y)
Supplement: Supplementary file 4 — Reporting Summary [file 42003_2022_3047_MOESM4_ESM.pdf]

nature portfolio

Corresponding author(s): Yue-zhong Li and Changsheng Wu

Last updated by author(s): 2022/01/03

Reporting Summary

Nature Portfolio wishes to improve the reproducibility of the work that we publish. This form provides structure for consistency and transparency in reporting. For further information on Nature Portfolio policies, see our Editorial Policies and the Editorial Policy Checklist.

Statistics

For all statistical analyses, confirm that the following items are present in the figure legend, table legend, main text, or Methods section.

n/a	Confirmed	
☐	☒	The exact sample size (*n*) for each experimental group/condition, given as a discrete number and unit of measurement
☐	☒	A statement on whether measurements were taken from distinct samples or whether the same sample was measured repeatedly
☐	☒	The statistical test(s) used AND whether they are one- or two-sided<br>*Only common tests should be described solely by name; describe more complex techniques in the Methods section.*
☒	☐	A description of all covariates tested
☐	☒	A description of any assumptions or corrections, such as tests of normality and adjustment for multiple comparisons
☐	☒	A full description of the statistical parameters including central tendency (e.g. means) or other basic estimates (e.g. regression coefficient) AND variation (e.g. standard deviation) or associated estimates of uncertainty (e.g. confidence intervals)
☐	☒	For null hypothesis testing, the test statistic (e.g. *F*, *t*, *r*) with confidence intervals, effect sizes, degrees of freedom and *P* value noted<br>*Give P values as exact values whenever suitable.*
☒	☐	For Bayesian analysis, information on the choice of priors and Markov chain Monte Carlo settings
☐	☒	For hierarchical and complex designs, identification of the appropriate level for tests and full reporting of outcomes
☐	☒	Estimates of effect sizes (e.g. Cohen's *d*, Pearson's *r*), indicating how they were calculated

Our web collection on statistics for biologists contains articles on many of the points above.

Software and code

Policy information about availability of computer code

Data collection	no software was used
Data analysis	no software was used

For manuscripts utilizing custom algorithms or software that are central to the research but not yet described in published literature, software must be made available to editors and reviewers. We strongly encourage code deposition in a community repository (e.g. GitHub). See the Nature Portfolio guidelines for submitting code & software for further information.

Data

Policy information about availability of data

All manuscripts must include a data availability statement. This statement should provide the following information, where applicable:

- Accession codes, unique identifiers, or web links for publicly available datasets
- A description of any restrictions on data availability
- For clinical datasets or third party data, please ensure that the statement adheres to our policy

The protein sequences of glycosyltransferases are deposited in GenBank under the following accession numbers: YjiC (AAU40842), BsGT-1 (CUB50191), BaGT (ADP31706), BgGT (SCA85980), BpGT (ARA85718) Details are presented in the Supplementary Table 9. All source data underlying the graphs and charts presented in the main figures are available in the Supplementary Data 1. All other data that support the findings of this study are available from the corresponding authors upon reasonable request.

Field-specific reporting

Please select the one below that is the best fit for your research. If you are not sure, read the appropriate sections before making your selection.

☒ Life sciences ☐ Behavioural & social sciences ☐ Ecological, evolutionary & environmental sciences

For a reference copy of the document with all sections, see nature.com/documents/nr-reporting-summary-flat.pdf

Life sciences study design

All studies must disclose on these points even when the disclosure is negative.

Sample size	Sample sizes are based on the Glycosyltransferases, Acceptor (Epothlone B) and Sugar donors.
Data exclusions	so
Replication	We confirm that the successful attempts at replication
Randomization	Samples were allocated into experimental groups randomly
Blinding	The investigators were blinded to group allocation during data collection

Reporting for specific materials, systems and methods

We require information from authors about some types of materials, experimental systems and methods used in many studies. Here, indicate whether each material, system or method listed is relevant to your study. If you are not sure if a list item applies to your research, read the appropriate section before selecting a response.

Materials & experimental systems

n/a	Involved in the study	
☐	☒	Antibodies
☐	☒	Eukaryotic cell lines
☒	☐	Palaeontology and archaeology
☒	☐	Animals and other organisms
☒	☐	Human research participants
☒	☐	Clinical data
☒	☐	Dual use research of concern

Methods

n/a	Involved in the study	
☒	☐	ChIP-seq
☒	☐	Flow cytometry
☒	☐	MRI-based neuroimaging

Antibodies

Antibodies used

1. beta Tubulin Rabbit Monoclonal Antibody and 2. Alexa Fluor 488 goat anti-rabbit IgG (H+L)

Validation

1. Cat No. AF1216, Beyotime. 2. Cat No. A0428, Beyotime. https://www.beyotime.com/
Yang HB, Yang X, Cao J, Li S, Liu YN, Suo ZW, Cui HB, Guo Z, Hu XD. cAMP-dependent protein kinase activated Fyn in spinal dorsal horn to regulate NMDA receptor function during inflammatory pain. J Neurochem. 2011 Jan;116(1):93-104.
Peng H, Wu Y, Zhang Y. Efficient delivery of DNA and morpholinos into mouse preimplantation embryos by electroporation. PLoS One. 2012; 7(8):e43748.
Wang X, Bai J, Zhang L, Wang X, Li Y, Jiang P . Poly(A)-binding protein interacts with the nucleocapsid protein of porcine reproductive and respiratory syndromevirus and participates in viral replication. Antiviral Res. 2012 Dec;96(3):315-23.
Li X, Li Z, Li N, Qi J, Fan K, Yin P, Zhao C, Liu Y, Yao W, Cai X, Wang L, Zha X. MAGI2 enhances the sensitivity of BEL-7404 human hepatocellular carcinoma cells to staurosporine-induced apoptosis by increasing PTEN stability. Int J Mol Med. 2013 Aug;32(2):439-47.
Wang HQ, Meng S, Li ZR, Peng ZG, Han YX, Guo SS, Cui XL, Li YH, Jiang JD. The antiviral effect of 7-hydroxyisoflavone against Enterovirus 71 in vitro. J Asian Nat Prod Res. 2013 Apr;15(4):382-9.
Tu C, Li N, Zhu L, Zhou L, Su Y, Li P, Zhu X. Cationic long-chain hyperbranched poly(ethylene glycol)s with low charge density for gene delivery. Polym. Chem. 2013;4:393-401.

Eukaryotic cell lines

Policy information about cell lines

Cell line source(s)	The human liver carcinoma cell line HepG2 and human hepatocyte cell line HL-7702 were purchased from Gaining (Gaining Bio. Co., Ltd, Shanghai, China).
Authentication	The human liver carcinoma cell line HepG2 and human hepatocyte cell line HL-7702 were STR identified correctly, and the STR identification maps were matched.
Mycoplasma contamination	No Mycoplasma contamination
Commonly misidentified lines (See ICLAC register)	The cell lines in this study were all STR identified correctly, and there were no misidentified lines. All the STR identified cell lines were for the MTT and Immunofluorescence assays.
